# Foxe1 deficiency impairs thyroid fate while supporting lung differentiation

**DOI:** 10.1038/s44319-026-00841-1

**Published:** 2026-06-21

**Authors:** Bárbara F Fonseca, Cindy Barbée, Sema Elif Eski, Pierre Gillotay, Daniel Monteyne, David Perez Morga, Samuel Refetoff, Sumeet Pal Singh, Sabine Costagliola, Mírian Romitti

**Affiliations:** 1 Institut de Recherche Interdisciplinaire en Biologie Humaine et Moléculaire, Université Libre de Bruxelles, Brussels, Belgium; 2 Laboratory of Molecular Parasitology, IBMM & Center for Microscopy and Molecular Imaging, Université Libre de Bruxelles, Brussels, Belgium; 3 Departments of Medicine, Pediatrics and Committee on Genetics, The University of Chicago, Chicago, IL USA

**Keywords:** Chromatin, Transcription & Genomics, Development

## Abstract

Patterning of mammalian endoderm into lung and thyroid lineages depends upon a correct early expression of a homeobox domain-containing transcription factor, Nkx2-1. However, the gene networks distinguishing the differentiation of those lineages remain largely unknown. In this work, by using mouse stem cell lines, scRNA-seq, and transcriptomic and chromatin accessibility profiling, we show that Foxe1 knockout impairs Nkx2-1+ cell differentiation and maturation into thyroid follicular-like cells. Concomitantly, a subset of Foxe1 null/Nkx2-1+ cells follows a lung epithelial differentiation program and form lung-like organoids harboring cells transcriptionally similar to mouse fetal lung types. Chromatin analyses reveal that, while accessibility at the Pax8 locus is reduced, loci associated with lung programs are in an open configuration, indicating that lung fate can be adopted without additional chromatin remodeling. These findings demonstrate that Foxe1 loss destabilizes thyroid commitment but also creates a permissive state in which Nkx2-1+ foregut progenitors can adopt an alternative lung fate. Our study illustrates how the interplay between transcription factors and chromatin context governs lineage decisions in vitro and provides a platform to investigate mechanisms underlying organ specification and plasticity.

## Synopsis

Foxe1 loss disrupts thyroid differentiation and instead promotes lung-like cell specification from Nkx2-1+ foregut progenitors. These findings reinforce the key role of Foxe1 in thyroid lineage commitment and cell fate plasticity.

- Foxe1 deficiency impairs thyroid follicular cell differentiation and maturation.
- Foxe1-null Nkx2-1+ cells can adopt a lung-like epithelial fate and form lung-like organoids.
- Lung-associated chromatin regions remain accessible even in the presence of Foxe1, and upon Foxe1 loss this permissive chromatin state facilitates activation of the lung differentiation program.

## Abstract (web summary)

Foxe1 loss disrupts thyroid differentiation and instead promotes lung-like cell specification from Nkx2-1+ foregut progenitors. These findings reinforce the key role of Foxe1 in thyroid lineage commitment and cell fate plasticity.

## Graphical abstract

**Figure:**
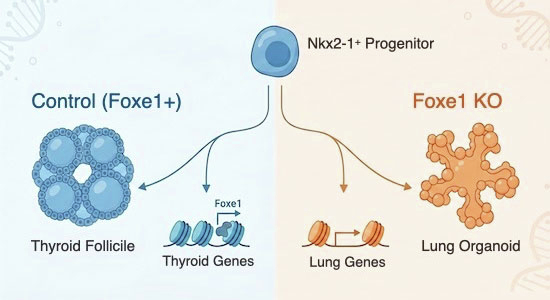

**Figure:**
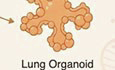

## Introduction

Embryonic development comprises a stepwise progression towards restriction of cell potentiality and acquisition of a differentiated cellular state. There are various molecular mechanisms controlling cell fate commitment, involving cellular response to extracellular cues, chromatin remodeling, and transcription factor activation of lineage-related cis-regulatory regions. Unveiling the molecular events triggering lineage decisions is not only crucial to understand the biology behind those phenomena but also to allow in vitro cell engineering, which is a potential avenue for disease modeling and regenerative applications in medicine.

During endoderm organogenesis, the space and time coordinated expression of transcription factors such as Sox2, Cdx, Foxa2, Hhex, Pdx1, among others, patterns the endoderm layer into more specific cell lineages (Kraus and Grapin-Botton, 2012; Zorn and Wells, 2009). For thyroid and lung derivation, for example, it is broadly known that the homeodomain-containing factor *Nkx2-1* (also called thyroid transcription factor 1; *Ttf-1*), is the first gene for which expression is detected in specific domains of the ventral anterior foregut endoderm, where the thyroid and lung primordia arise around embryonic days 8–9 during mouse embryogenesis (Cardoso and Lu, 2006; Lazzaro et al, 1991). In addition, *Nkx2-1* expression is observed throughout lung and thyroid embryonic and adult life. Therefore, it is not surprising that NKX2-1 gene mutations, in humans and mice, engender a variety of thyroid and pulmonary abnormalities, as well as neurological defects (Butt et al, 2008; Herriges and Morrisey, 2014; Willemsen et al, 2005).

Besides the requirement of Nkx2-1 signaling for both thyroid and lung lineages, the molecular pathways leading to their specification are still poorly understood. For this reason, in early directed differentiation protocols to derive thyroid/lung lineages from mouse embryonic stem cells (mESC), a dual generation of thyroid/lung Nkx2-1 progenitor cells was obtained (Longmire et al, 2012). A better understanding of the different requirements for Fgf and Wnt pathways activation was key to separately drive each lineage in mESCs, after an in vitro step of anterior foregut specification (Dame et al, 2017; Kurmann et al, 2015; Mou et al, 2012; Serra et al, 2017). More recent reports are helping to shed light on the early stages of thyroid and lung organogenesis (Haerlingen et al, 2019, 2023; Ikonomou et al, 2020; Rankin et al, 2021; Vandernoot et al, 2021). Overall, the data indicate an intricate relationship among thyroid/lung lineages at early stages of mammalian development.

At the time of thyroid specification, besides Nkx2-1, cells from thyroid anlage are identified by the restricted expression of three other transcription factors: Pax8, Hhex, and Foxe1 (López-Márquez et al, 2021). According to proposed mechanisms for thyroid specification (Parlato et al, 2004), the onset of Foxe1 expression is downstream to those cited players and Foxe1 (also called thyroid transcription factor 2; Ttf-2) is key to the induction of more thyroid-restricted markers, such as *Thyroglobulin* and *Thyroperoxidase* (*Tpo)* (Aza-Blanc et al, 1993; Francis-Lang et al, 1992; López-Márquez et al, 2019; Santisteban et al, 1992). Foxe1 is a member of the Forkhead family, a group of transcription factors classically involved in endoderm lineage decisions, acting directly to induce the transcription program of a particular cell lineage but also potentially repressing alternative cell fates (Golson and Kaestner, 2016; Li et al, 2016; Sekiya and Suzuki, 2011; Zaret and Carroll, 2011). Interestingly, at the stage of thyroid/lung lineage commitment in the anterior foregut endoderm, *Foxe1* is expressed throughout the anterior foregut domain, but it is specifically absent in the lung primordium (De Felice and Di Lauro, 2004; Kuwahara et al, 2020), suggesting that *Foxe1* expression in this region is not compatible with the correct lung lineage establishment.

In the present work, we investigated the effect of Foxe1 loss-of-function in regulating differentiation of thyroid lineage using mouse ESC-derived organoids (Antonica et al, 2012; Romitti et al, 2021). Here, we show that in the absence of Foxe1, thyroid commitment is severely impaired, and the few Nkx2-1/Pax8+ precursors that emerge fail to generate mature thyroid follicular structures. Unexpectedly, we find that a large subset of Nkx2-1+ cells instead diverges toward an alternative differentiation trajectory, giving rise to epithelial lung organoids.

## Results

### Foxe1 is required for thyroid development in vitro

**Figure 1:**
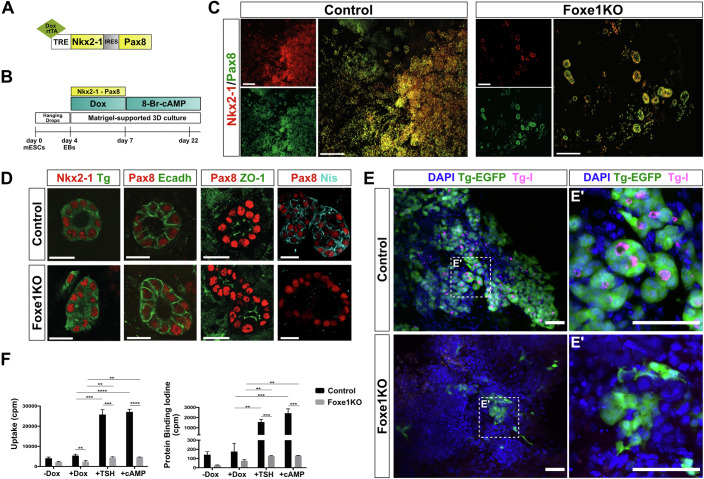
Foxe1 gene is required for efficient thyroid differentiation in vitro. (**A**) Schematic representation of the tetracycline-inducible cassette to drive co-expression of Nkx2-1 and Pax8. (**B**) Diagram of the 22-day differentiation protocol for mESCs. (**C**–**F**) Immunostaining and organification assays performed at day 22 of differentiation. (**C**) Immunostaining of double-positive Nkx2-1 and Pax8 thyrocytes in control and Foxe1KO cells. (**D**, **E**) Immunostaining of a panel of thyroid markers in control and Foxe1KO cells. (**F**) Iodide uptake and organification assays. Histograms represent the radioactivity (Uptake-cpm) of the cell iodine-125 uptake (left) and the radioactivity of protein-bound [^125^I] (PBI-cpm) measured using a γ-counter (right) (mean +/−SEM; *n* = 3 biological replicates). Scale bars: 150 µm (**C**); 20 µm (**D**); 50 µm (**E**). Tg thyroglobulin, Ecadh E-cadherin, Tg-I iodinated thyroglobulin. Source data are available online for this figure.

Seminal works have previously shown that Foxe1 depletion/dysfunction in the thyroid gland results in various thyroid defects, from the deregulation of key players involved in the thyroid hormone production pathway up to the agenesis of the gland (Clifton-Bligh et al, 1998; De Felice et al, 1998; Fernández et al, 2013). To further investigate the role of Foxe1 in thyroid follicular cells and to validate our mESC-derived thyroid organoid model to study genes causing hypothyroidism, we mutated Foxe1 loci in mESCs derived from our previously established transgenic line (A2lox-Nkx2-1-Pax8 line). In this line, transient overexpression of Nkx2-1 and Pax8, followed by 2-week treatment with hTSH or c-AMP analogs, yields self-organized thyroid follicles that secrete T4 hormone in vitro with high efficiency (Antonica et al, 2012) (Fig. 1A,B). In addition, differentiation can be visually assessed using an EGFP transgene inserted into the hypoxanthine phosphoribosyltransferase (HPRT) locus of mESC lines under the control of a bovine-responsive thyroglobulin (Tg) promoter (Romitti et al, 2021).

**Figure EV1:**
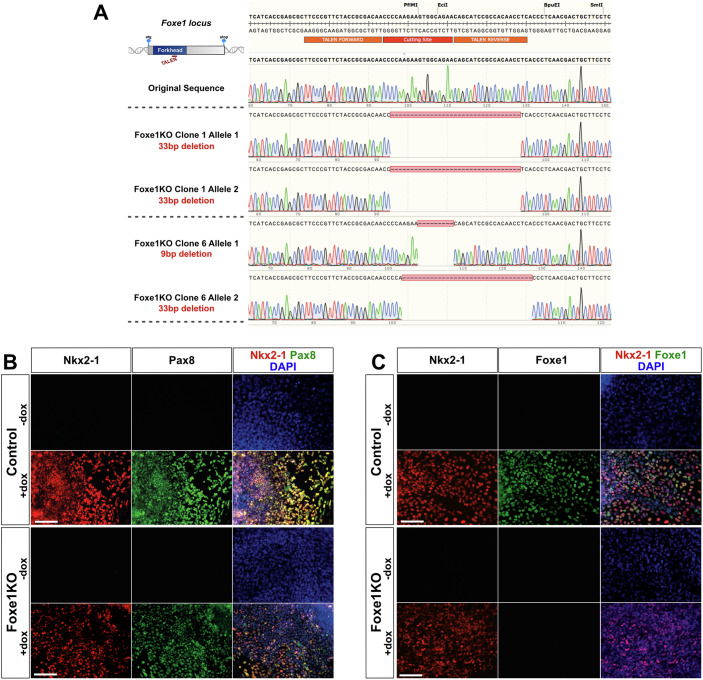
Generation and validation of Foxe1 KO mESC lines. (**A**) Genomic profiling of two Foxe1 KO mESC clones. One clone presents a homologous deletion of 33 bp, and the second one possesses a heterozygous deletion of 9 and 33 bp at the Foxe1 locus, respectively. (**B**, **C**) Immunostaining of control and Foxe1KO mESCs at day 7, after Dox-mediated induction of Nkx2.1-Pax8 during 3 days (day 4–day 7). (**B**) The Dox-inducible co-expression of NKX2.1 and PAX8 is not altered by genome-editing manipulations in mutated clones. (**C**) Foxe1KO mESCs express NKX2.1, while Foxe1 protein expression is abolished (day 7). Scale bars: 150 µm.

**Figure EV2:**
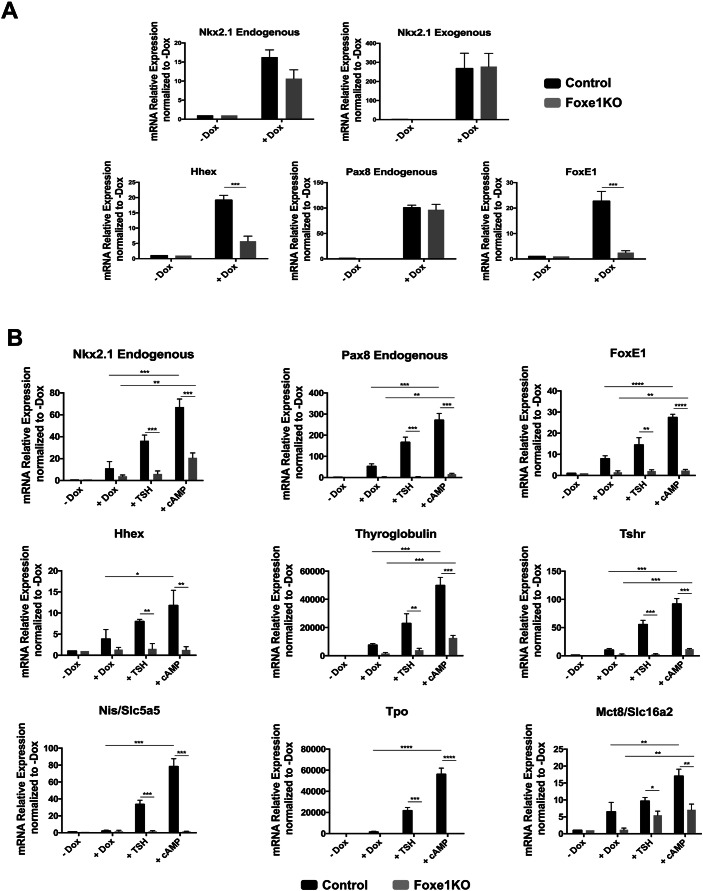
Foxe1 depletion in mESCs abolishes thyroid follicle differentiation. RT-qPCR analyses of thyroid-expressed genes in control and Foxe1KO cells at day 7 (**A**) and day 22 (**B**) after Dox-mediated induction (day 4–day 7) followed by TSH ( + TSH) or 8-br-cAMP ( + cAMP) treatment until day 22. (**A**) At day 7 of differentiation, downregulation of some thyroid genes such as Hhex and Foxe1 is already observed in Foxe1KO cells, whereas Nkx2-1 and Pax8 endogenous expression is not affected. Observe that Dox-mediated induction of Nkx2-1/Pax8 was successful since exogenous expression of Nkx2-1 is not affected in both lineages. (**B**) Expression of endogenous thyroid genes at day 22 is drastically reduced in Foxe1KO cells ( + TSH and +cAMP conditions) in comparison with the control line. Relative expression of each transcript is presented as fold change compared to untreated cells (−Dox) (mean +/−SEM; *n *= 3 biological replicates). Unpaired *t* test was used for statistical analysis. *P < 0.05, **P < 0.01, ***P < 0.001. Dox doxycycline.

To obtain Foxe1 knockout (KO) lines, Foxe1 loci were edited using TALEN technology, and clones selected contained mutations that resulted in a premature stop codon (Fig. EV1A). After validation of the Foxe1KO lines with respect to maintenance of mESC pluripotency and responsiveness to the Nkx2-1/Pax8 tetracycline-inducible cassette (Fig. EV1B,C), control and Foxe1KO lines were subjected to the differentiation protocol (Fig. 1A,B). Three days after doxycycline treatment (day 7 of differentiation protocol), control and Foxe1KO lines are similarly efficient to activate exogenous expression of the artificial Nkx2-1/Pax8 transgenes (Fig. EV2A). This exogenous transgene expression leads to the activation of endogenous Nkx2-1 and Pax8 loci (Antonica et al, 2012). All lines successfully achieved overexpression of the endogenous loci of Nkx2-1 and Pax8. In contrast, the expression of Hhex and Foxe1 was drastically decreased in Foxe1KO cells (Fig. EV2A).

At the end of the differentiation protocol (Fig. 1B), an inhibitory effect of Foxe1 depletion on thyroid formation in vitro was clearly observed. First, we detected, by RT-qPCR, a strong reduction in mRNA levels of many thyroid-related genes (Fig. EV2B). Moreover, the efficiency of thyroid follicular cell generation was markedly reduced in the absence of Foxe1 compared with the control line (Fig. 1C). Although the formation of scarce monolayer follicular structures could still be detected, the protein expression of sodium/iodide symporter Nis (Slc5a5), a transporter essential for thyroid function, was drastically reduced (Fig. 1D). Finally, while thyroid follicles from the control line displayed proper accumulation of iodinated Tg (Tg-I; Fig. 1E) in the follicular lumen, quantified by high uptake of radioactive iodide and iodide binding to Tg, these processes were significantly reduced in Foxe1-depleted cells (Fig. 1F). Because *Tshr* mRNA levels were drastically low in the absence of Foxe1 (Fig. EV2B), we replaced the 2-weeks recombinant hTSH treatment with a cyclic AMP (cAMP) analog, as TSH ligands signal through the cAMP pathway (Kimura, 2001). The cAMP treatment produced similar results regarding thyroid-related gene expression and iodide organification in vitro (Figs. 1F and EV2B). Overall, these results demonstrate the drastic impairing effect of Foxe1 knockout on thyroid lineage derivation in vitro and validate our mESC-model to investigate genes involved in thyroid dysgenesis.

### Absence of Foxe1 permits lung differentiation in vitro

**Figure 2:**
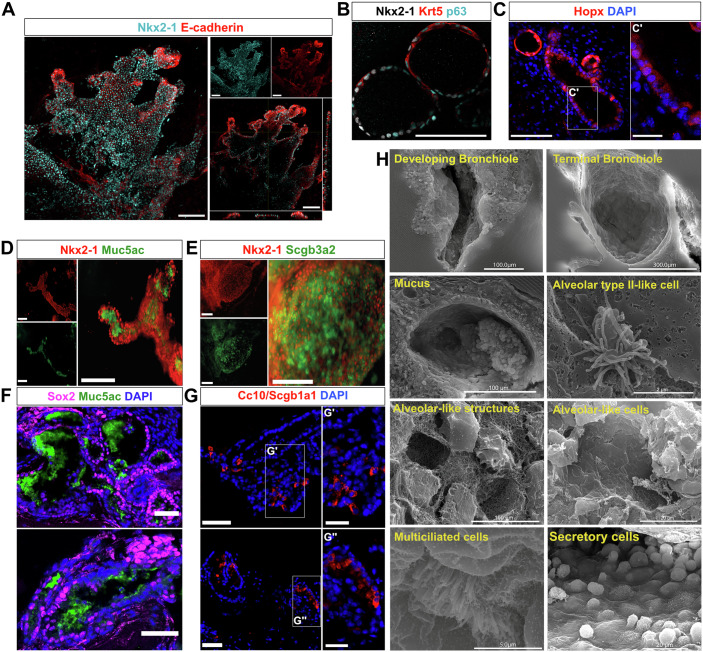
Generation of pulmonary structures in the absence of Foxe1. (**A**) Orthogonal view by confocal microscopy of in vitro lung-like structures identified by immunostaining of Nkx2-1 and E-cadherin at day 22 of the differentiation protocol. (**B**–**G**) Immunostaining of Foxe1KO mESC-derived organoids with a panel of lung-related markers: basal cells (Nkx2-1, Krt5, and P63) (**B**); alveolar cells (Hopx) (**C**); goblet cells (Nkx2-1, Sox2, and Muc5ac) (**D**, **F**) and secretory (club) cells (Nkx2-1 and Scgb3a2, Scgb1a1) (**E**, **G**). (**H**) Scanning electron microscopy at day 22 Foxe1KO mESC-derived organoids exhibiting the morphology of typical lung cell types, including airway and alveolar cells at fetal-like developmental stages. Scale bars: 100 µm (**A**); 25 µm (**B**); 100 µm (**C**, left); 25 µm (**C**, right); 150 µm (**D**, **E**), 50 µm (**F**, **G**), and 25 µm (**G’**). Source data are available online for this figure.

**Figure EV3:**
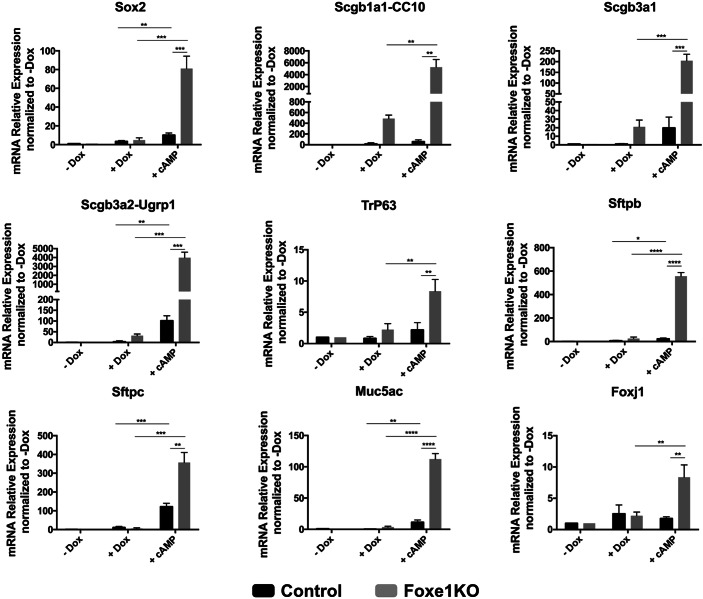
In vitro generation of pulmonary structures. RT-qPCR of in vitro-generated Foxe1KO structures after Dox-mediated induction of Nkx2.1-Pax8 followed by 8-br-cAMP treatment until day 22. mRNA expression of endogenous relevant pulmonary genes at day 22. Observe upregulation of lung-related markers in the +cAMP condition compared with untreated cells (-Dox) and dox-induced only condition ( + Dox). Relative expression of each transcript is presented as fold change compared to untreated cells (−Dox) (mean +/−SEM; *n* = 3 biological replicates). Unpaired *t* test was used for statistical analysis. *P < 0.05, **P < 0.01, ***P < 0.001.

Besides the impairment of thyroid generation in Foxe1KO mESCs, we observed an additional, albeit striking, phenotype: the self-assembly of Nkx2-1-expressing cells into epithelial structures with a morphology clearly different from thyroid follicles, in organoids derived from Foxe1KO cells (Fig. 2A). Because Nkx2-1 is involved in the specification of another ventral foregut derivative, the lung (Cardoso and Lu, 2006; Lazzaro et al, 1991), we wondered whether the appearance of these 3D structures might indicate unexpected generation of lung tissue in vitro. By immunostaining analyses at day 22, we observed that these Nkx2-1+ organoids indeed expressed typical lung-related markers, such as Trp63/Krt5 (basal cells), Hopx (alveolar-like cells), Muc5ac (goblet cells), Scgb1a1/CC10, and Scgb3a2 (club cells) (Fig. 2B–G). In addition, upregulation of lung-related transcripts was observed in Foxe1KO cells, as detected by RT-qPCR (Fig. EV3A). Ultrastructural microscopy of these Foxe1KO-derived organoids indicated the presence of bronchiole-like structures and cells exhibiting the morphology suggestive of lung cell types, such as multiciliated cells, mucus-secreting cells (goblet cells), and alveolar-like cells organized into developing alveolar sacs (Fig. 2H). Altogether, these results suggest that lung structures are formed from Foxe1-depleted mESCs.

### Characterization of thyroid and lung cell populations derived from Nkx2-1_mKO2 reporter control and Foxe1KO lines

**Figure EV4:**
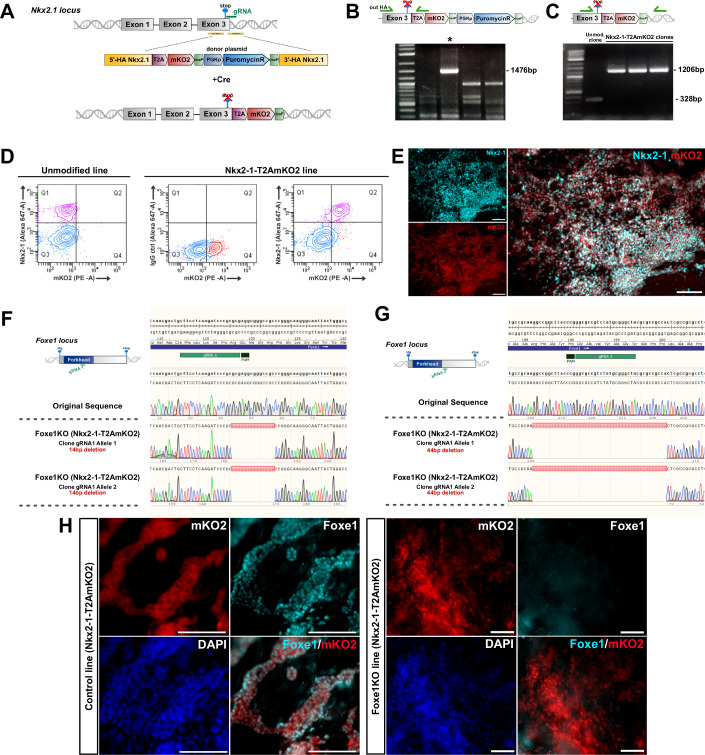
Generation of endogenous Nkx2-1 reporter lines. (**A**) Schematic representation of the CRISPR-mediated knock-in strategy to insert T2AmKO2 cassette in 3’-UTR region of Nkx2-1 loci. (**B**, **C**) PCR screening of knock-in generated clones before (**B**) and after (**C**) PuroR gene excision. Asterisk (*) on the image in (**B**) indicates a correctly integrated clone. (**D**, **E**) Validation of the Nkx2-1-T2AmKO2 line. (**D**) Cells from day 14 of the thyroid differentiation protocol were subjected to Nkx2-1 immunostaining followed by flow cytometry. Observe a double-positive stained population in Q2, only in the cAMP-treated cells derived from the Nkx2-1-T2AmKO2 line. (**E**) Images showing Nkx2-1 and mKO2 co-staining at day 10 2D culture. (**F**–**H**) Generation of Foxe1KO/Nkx2-1 reporter line. (**F**, **G**) Genomic profiling of a Foxe1KO/Nkx2-1 reporter clones obtained with guide RNAs targeting inside (**F**) or outside (**G**) the Forkhead domain. (**H**) Foxe1 immunostaining of control and Foxe1KO/Nkx2-1 reporter cells subjected to thyroid differentiation (day 22). Absence of Foxe1 protein expression is observed in Foxe1KO cells. Scale bars: 100 µm.

**Figure EV5:**
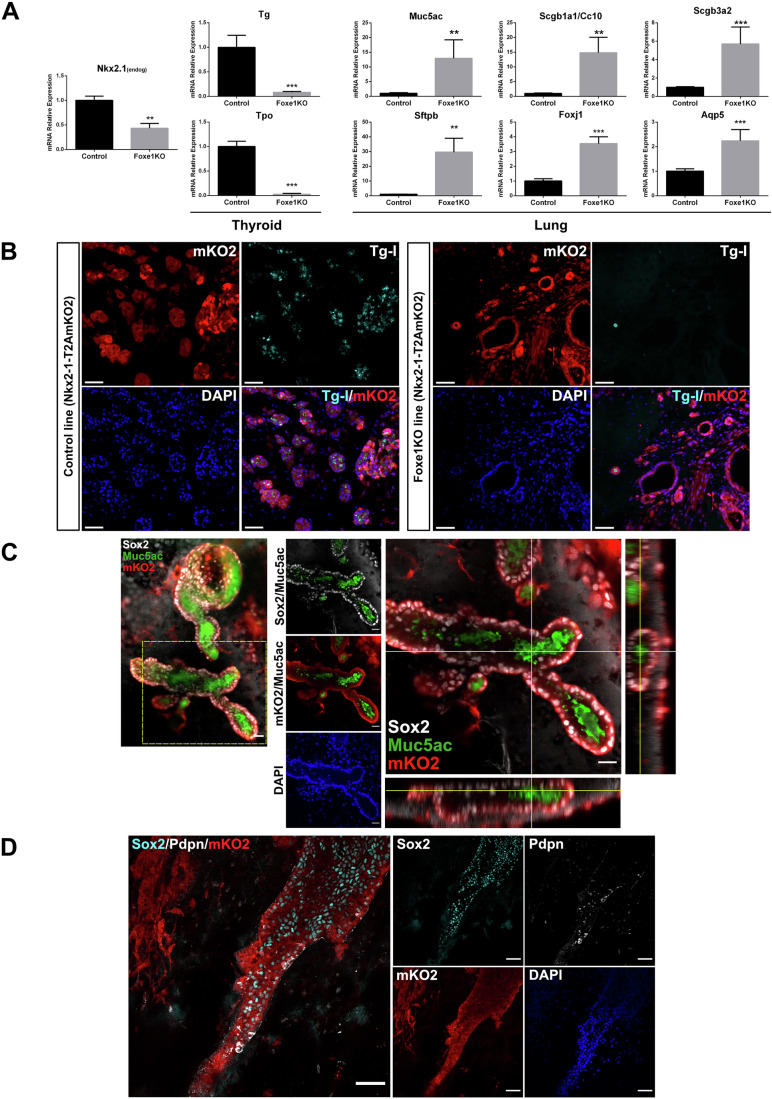
Thyroid and lung differentiation in control and Foxe1KO Nkx2-1 reporter lines. (**A**) RT-qPCR analyses of thyroid and lung expressed genes in control and Foxe1KO Nkx2-1 reporter lines. Observe the reduction of Tg and Tpo thyroid genes, whereas lung markers are upregulated in Foxe1KO cells. (**B**) Control Nkx2-1 reporter cells differentiated into thyroid follicles, containing iodinated-Tg (Tg-I) in the lumen. mKO2 immunostaining corresponds to the Nkx2-1 endogenous expression. (**C**, **D**) Foxe1KO Nkx2-1 reporter cells differentiate into airway organoids. (**C**) Immunostaining for Sox2, mKO2 and Muc5ac. Higher magnification and orthogonal views by confocal microscopy of the lung organoid depicted in the image on the right. (**D**) Immunostaining for Sox2, Pdpn, and mKO2. Scale bars: 50 µm (**B**, **D**), 25 µm (**C**).

**Figure EV6:**
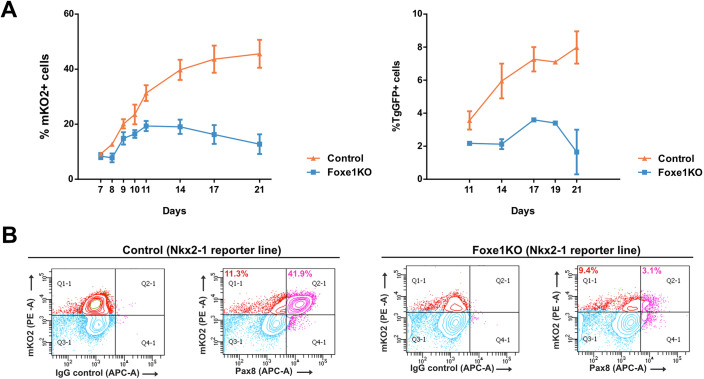
Quantification of Nkx2-1-expressing cells in control and Foxe1KO/Nkx2-1 reporter lines. (**A**) Flow cytometry quantification of cells expressing Nkx2-1 reporter (mKO2 + , left) and bovineTg reporter (TgGFP + , right) in control and Foxe1KO lines. (**B**) Pax8 immunostaining followed by flow cytometry analyses at day 22. Numbers shown in Q2-1 quadrant correspond to the percentage of double-positive Pax8 (APC) and mKO2 (PE) in control and Foxe1KO cells.

Foxe1 depletion in mESCs results in a decreased generation of thyroid cells and the appearance of lung-related cell types. This observation prompted us to better characterize the cell populations derived from Foxe1KO cells by scRNAseq. For this, we first designed Nkx2-1 reporter lines, allowing easier identification and isolation of thyroid and lung cell types. A mKO2 fluorescent tag was inserted into the 3’ end of the endogenous Nkx2-1 loci of the control A2lox-Nkx2-1-Pax8 line (Fig. EV4A-C). The reporter fluorescence allowed tracking of Nkx2-1+ cells by microscopy and quantification of the differentiation efficiency by flow cytometry (Figs. EV5B–D and EV6A,B). Foxe1KO/Nkx2-1 reporter lines were created and differentiated using our differentiation protocol. The same thyroid Foxe1KO phenotype (i.e., disruption of thyroid generation efficiency and functionality) was observed in these new lines. Notably, the introduction of the mKO2 tag enabled quantification of the proportions of thyroid and non-thyroid Nkx2-1–expressing cells generated during differentiation (Fig. EV6). At early stages, up to day 10, the proportion of Nkx2-1⁺ (mKO2⁺) cells was similar in both control and Foxe1KO lines (Fig. EV6A). However, from day 11 onward, the proportion of Nkx2-1⁺ (mKO2⁺) cells in the control line increased steadily, resulting by day 22 in an approximately 3.5-fold difference compared with the Foxe1KO line (46% in control versus 13% in Foxe1KO) (Fig. EV6A). In addition, co-staining with Pax8 revealed that around 42% of the cells generated from the control line show a thyroid identity with ~80% of Nkx2-1⁺ cells co-expressing Pax8. In contrast, Nkx2-1⁺/Pax8⁺ thyrocytes represented only ~3% of the total cell population in the Foxe1KO line and ~25% of the total Nkx2-1⁺ cells (Fig. EV6B). Similarly, a lower proportion of bvTg_prom/EGFP+ cells, which tags a subset of mature thyrocytes (Romitti et al, 2021), is observed in the Foxe1KO line (Fig. EV6A).

### Single-cell RNA-seq analysis reveals that Foxe1KO cells lack functional thyroid transcripts and evidence of aberrant Nkx2-1+ cells

**Figure 3:**
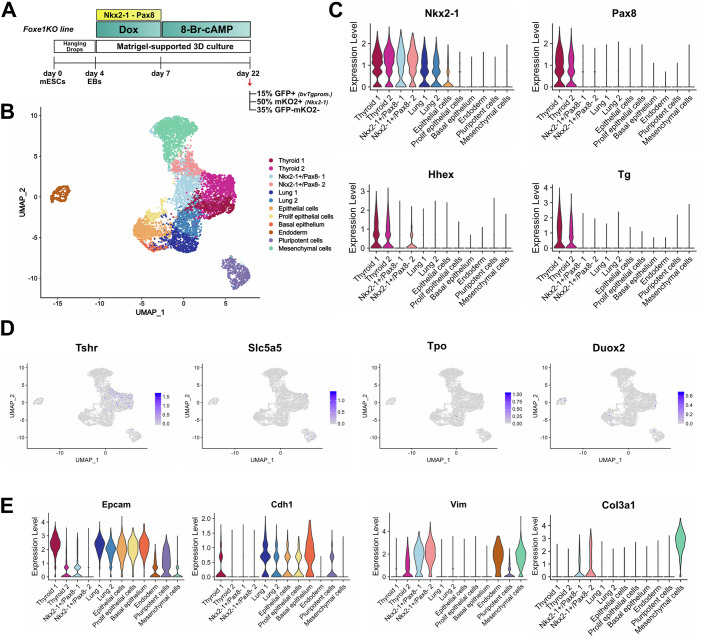
Single-cell RNA-seq analysis of Foxe1KO-derived thyroid cells. (**A**) Schematic diagram of the differentiation protocol and purification of distinct cell populations at day 22 for scRNA-seq experiment. (**B**) Unsupervised clustering of 7523 single-cell profiles, colored by cluster assignment. (**C**) Violin plots featuring the average expression per cluster of early thyroid markers. (**D**) UMAP expression plots of markers for thyroid maturation. (**E**) Violin plots featuring average expression per cluster of selected epithelial and mesenchymal markers.

**Figure EV7:**
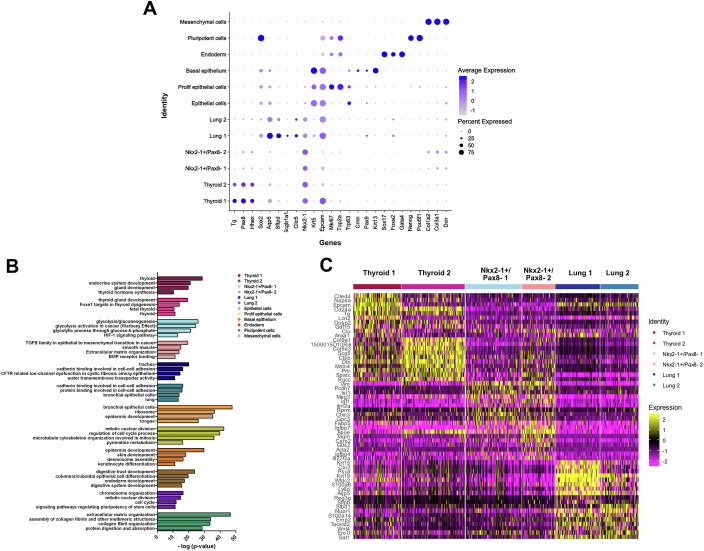
scRNAseq profiling of Foxe1KO cells. (**A**) Average expression per cluster of selected markers used to define cluster identity. (**B**) Gene Ontology (GO) and pathway analysis performed with the top 50 differentially expressed genes in each cluster. (**C**) Heatmap of the top 50 differentially expressed genes in each cluster expressing Nkx2-1.

For the single-cell profiling of Foxe1KO cells, we enriched by FACS three cell populations: Nkx2-1_mKO2 + /bvTg_prom_EGFP+ (15%), Nkx2-1_mKO2 + /bvTg_prom_EGFP- (50%), and Nkx2-1_mKO2- (35%) (Fig. 3A). A total of 12,000 cells were profiled for scRNA-seq using the droplet-based assay from the 10X Genomics system. After quality control, we obtained 7523 cells. The profiled cells could be divided into 12 clusters composed of Nkx2-1 positive cells (i.e., Thyroid 1, Thyroid 2, Lung 1, Lung 2, Nkx2-1+Pax8- 1, Nkx2-1+Pax8- 2) and Nkx2-1-negative cells (i.e., epithelial cells, prolif. epithelial cells, basal epithelium, pluripotent cells, mesenchymal cells) (Fig. 3B). Gene Ontology and literature mining were used to characterize the clusters based on top differentially expressed genes (Fig. EV7A–C). Among Nkx2-1 negative cells, epithelial-like cells are found in three clusters (702 broad epithelial cells, 244 prolif. epithelial cells, and 123 basal epithelial cells). In total, 1411 pan-mesenchymal cells (mesenchymal cells cluster), 280 cells expressing endoderm markers (endoderm cluster), and a remaining pluripotent stem cell population were also found (pluripotent cells cluster, 520 cells).

Six clusters are composed of cells expressing Nkx2-1. Thyroid 1 (722 cells) and Thyroid 2 (947 cells) clusters exhibit a typical thyroid signature characterized by the expression of *Pax8*, *Hhex*, and *Tg* (Figs. 3B,C and EV7A–C). Note that expression of mature markers such as *Tpo*, *Slc5a5*, and *Duox2* is undetectable, while *Tshr* levels are low, supporting our previous results (Fig. 3D). Among those thyroid clusters, the normalized average expression of *Pax8*, *Hhex*, and *Tg* appears to be lower in Thyroid 2 than in Thyroid 1 group. In addition, Thyroid 2 cell group lacks expression of *Epcam* and *Cdh1* (Figs. 3E and EV7A). Since E-cadherin expression is maintained throughout thyroid morphogenesis in vivo (Fagman et al, 2003), this finding suggests that Thyroid 2 cells are abnormal in vitro-derived thyrocytes not organized into follicular units.

We also identified two additional cell clusters characterized by strong *Nkx2-1* but absent *Pax8* expression (Nkx2-1 + /Pax8- 1 and Nkx2-1 + /Pax8- 2 clusters, with 843 and 489 cells, respectively). Their transcriptomic signature suggests that they are distinctly different from the thyroid and lung clusters. Those clusters lack *Epcam* and *Cdh1* expression but are instead enriched in mesenchymal-like markers such as *Vim*, *Acta2*, and *Col3a1* (Fig. 3E). Gene Ontology analysis shows enrichment in the “extracellular matrix/epithelial to mesenchymal transition” and “glycolysis/hypoxia pathways”, respectively (Fig. EV7B). As mesenchymal-like cells expressing Nkx2-1+ are not described during development, these cells likely reflect part of an in vitro Foxe1KO phenotype, along with the appearance of lung tissue in vitro. Interestingly, these cells do not express neural markers such as *Ascl1*, *Tubb3*, *Pax6*, and *Six3*, making it unlikely that they possess a Nkx2-1+ neural signature. Finally, thyroid C cells also express *Nkx2-1* (Nilsson and Williams, 2016), but no co-expression of *Calca*, *Foxa1*, and *Foxa2* were observed. Together, these observations suggest that these cells may represent an intermediate or aberrant differentiation state arising in vitro, potentially reflecting the combined effects of Foxe1 loss and culture conditions that do not support progression toward a fully specified lineage.

### Foxe1KO mESCs differentiate into multiple lung cell types harboring transcriptomic signatures encountered in E17.5 embryonic mouse lung tissue

**Figure 4:**
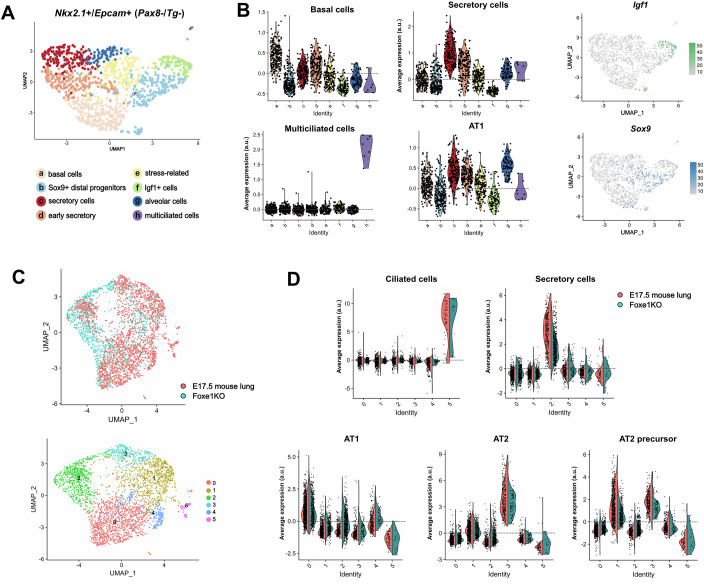
Single-cell RNA-seq analysis of Foxe1KO-derived lung cells. (**A**) 1155 cells co-expressing Nkx2-1 and Epcam (but not expressing Pax8 and Tg) were computationally isolated, re-clustered, and cellular transcriptome heterogeneity visualized using UMAP. (**B**) Average expression levels per cluster of indicated lung cell type signatures and UMAP expression plot of Igf1 and Sox9 normalized expression, defining two clusters of early lung progenitors. (**C**) UMAP plot of Foxe1KO cells (1155) integrated with E17.5 mouse lung Nkx2-1+Epcam+ cells (2000 cells) (upper graph). Six identified clusters contain both in vitro and in vivo-derived lung cells (bottom graph). (**D**) Average expression levels of indicated lung cell type signatures identified in both mouse lung (pink) and Foxe1KO cells (blue).

Lung-like cells originated from Foxe1KO cells are present as Lung 1 and Lung 2 clusters (668 and 574 cells, respectively). To better define specific lung cell types and avoid contamination with thyrocytes, we computationally selected cells expressing *Nkx2-1* and *Epcam*, but lacking *Tg* and *Pax8*, from the Foxe1KO dataset. After re-clustering and assigning the cell populations, we identified eight different lung subsets (Fig. 4A). Cell types were identified using a signature score index based on the top 20 marker genes expressed by several lung epithelial cell types found in the scRNAseq dataset of E17.5 mouse lung tissue (Frank et al, 2019). As this dataset includes lung epithelial cells derived from murine alveolar and terminal bronchioles, a literature mining was performed to define the signature score of basal cells found mainly in the upper airways (Dataset EV1). As a result, we identified cell populations with a strong signature of secretory cells (cluster c), basal cells (cluster a), multiciliated cells (cluster h), and a small group with a slightly enriched signature for alveolar type 1 (AT1) cells (cluster g). In addition, we identified a cluster enriched in both basal and secretory markers (cluster d), suggesting to refer to cells transitioning from basal to secretory cell fate (Fig. 4A,B). Finally, we detected two clusters, b and f, that were enriched in Sox9 and Igf1 transcripts, respectively (Fig. 4B). These factors are highly expressed in early lung development, with Sox9 specifically present in distal bud cells (Nikolić et al, 2018).

With the purpose of better characterizing Foxe1KO-derived lung cell types and to test the degree of similarity with in vivo encountered lung cells, we performed scRNAseq comparative analyses between the above cited E17.5 mouse lung dataset and Foxe1KO-derived lung cells (Frank et al, 2019; Stuart et al, 2019) (Fig. 4C,D). To do this, we integrated *Nkx2-1* + /*Epcam* + /*Tg*-/*Pax8*- cells from the Foxe1KO dataset with *Nkx2-1* + /*Epcam*+ cells from the in vivo data. The analysis revealed a high overlap among cells from the two datasets (Fig. 4C). Furthermore, when we tested for enriched signatures of multiple lung cell types based on the normalized average expression of marker genes, we detected the presence of alveolar cells (AT1, AT2, and AT2 precursors) in groups 0, 3, and 1, respectively, in both datasets (Fig. 4D). Ciliated and secretory cells were also identified, as shown in Fig. 4B. Of note, because the mouse fetal lung dataset is devoid of basal cells (Frank et al, 2019), this particular lung cell type could not be addressed with this integrated analysis.

In summary, Foxe1 depletion abrogates the proper differentiation of thyrocytes and instead allows the development of different lung cell types. In addition, the Foxe1KO-derived lung cells are similar to the cells encountered in vivo regarding the transcriptomic signature of the major lung cell type markers.

### Chromatin accessibility analysis of Nkx2-1-expressing cells

**Figure 5:**
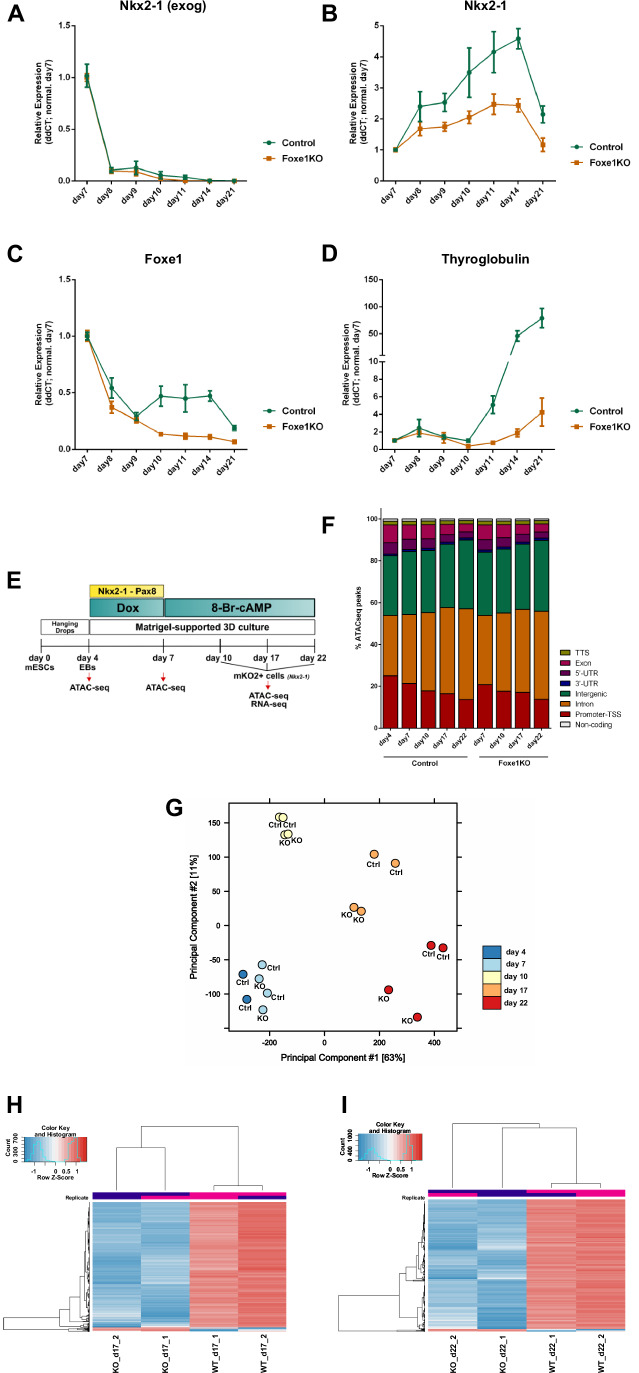
ATAC-seq profiling of control and Foxe1KO Nkx2-1 expressing cells at different stages of the differentiation protocol. (**A**–**D**) Relative mRNA expression of thyroid marker genes through different time points of culture. Relative expression of each transcript is presented as fold change compared to cells from the first time point (day 7) as mean +/−SEM (*n* = 6). Exog: exogenous. (**E**) Schematic representation of the differentiation protocol and time points of cell sorting of Nkx2-1+ (mKO2 + ) cells for ATAC-seq and RNA-seq analysis. (**F**) Total ATAC-seq peaks distribution in diverse genomic regions. (**G**) Principal component analysis (PCA) to compare total ATAC-seq peaks among time points. (**H**, **I**) Heatmaps of differentially accessible peaks between control and Foxe1KO at day 17 (**H**) and day 22 (**I**). Source data are available online for this figure.

Our results show that depletion of Foxe1 impairs proper differentiation of Nkx2-1 cells into thyrocytes in vitro while allowing the appearance of lung cell types. To identify potential molecular players involved in Foxe1 action, we performed a temporal analysis of the global chromatin accessibility of Nkx2-1-expressing cells, using bulk ATAC-seq. To develop an optimal experimental procedure, we first examined the kinetics of thyroid-related gene expression (e.g., *Nkx2-1*, *Foxe1*, and *Tg*) at key points of the protocol in control and Foxe1KO cells (Fig. 5A–D). After induction of the artificial Nkx2-1/Pax8 cassette between days 4 and 7, exogenous *Nkx2-1* mRNA levels are dramatically reduced on day 8 and it is virtually abolished around day 11 (Fig. 5A). Conversely, endogenous expression of Nkx2-1 increases rapidly as of day 7 and reaches a maximum at day 14 (Fig. 5B). Foxe1 is firstly induced by the direct action of exogenous Nkx2-1 and Pax8 at day 7, following a downregulation on days 7 and 9 (Fig. 5C). A second wave of induction occurs from day 9 onwards, likely due to the endogenous expression of Nkx2-1/Pax8, in conjunction with treatment with c-AMP (Antonica et al, 2012; Ortiz et al, 1997). As expected, *Tg* mRNA levels are very low in early stages of culture, but increase steadily after day 11, reaching the highest level at the end of the protocol (Fig. 5D). The temporal expression of these thyroid markers is consistent with the expected pattern of thyroid differentiation genes (Fernández et al, 2015; Romitti et al, 2021). These results suggest that events important for thyroid commitment in vitro occur before day 10, as high *Tg* levels are observed after this time point. Coincidentally, the increase in *Foxe1* expression occurs approximately one day before *Tg* expression onset.

Based on the kinetics of expression of lineage-specific genes, we decided to isolate Nkx2-1+ cells in the control and Foxe1KO samples by FACS at day 10 to identify early progenitors of the thyroid and lung lineages, and more differentiated cells at days 17 and 22. In addition, cells from day 4 and day 7, time points before and after doxycycline treatment, respectively, were also profiled (Fig. 5E). In total, ~90,000 ATAC-seq peaks were obtained per sample, which were distributed in different open genomic regions (Fig. 5F). PCA analysis suggests a gradual change in global chromatin accessibility that correlates with the timing of the differentiation protocol (Fig. 5G). Day 4 and day 7 samples are more similar to each other than the other late time points. In addition, no clear difference between the control and Foxe1KO is observed at day 7. Since the time point at day 7 corresponds to the end of doxycycline treatment, this implies that exogenous induction of Nkx2-1 and Pax8 results in a similar open chromatin landscape in both lines. ATAC-seq peaks originating from Nkx2-1+ cells (day 10, day 17, and day 22) show a gradual chromatin remodeling after day 10, which becomes more evident at days 17 and 22 (Fig. 5G–I).

### Enrichment in thyroid maturation markers in control Nkx2-1+ cells and the identification of predicted Foxe1 target genes

**Figure 6:**
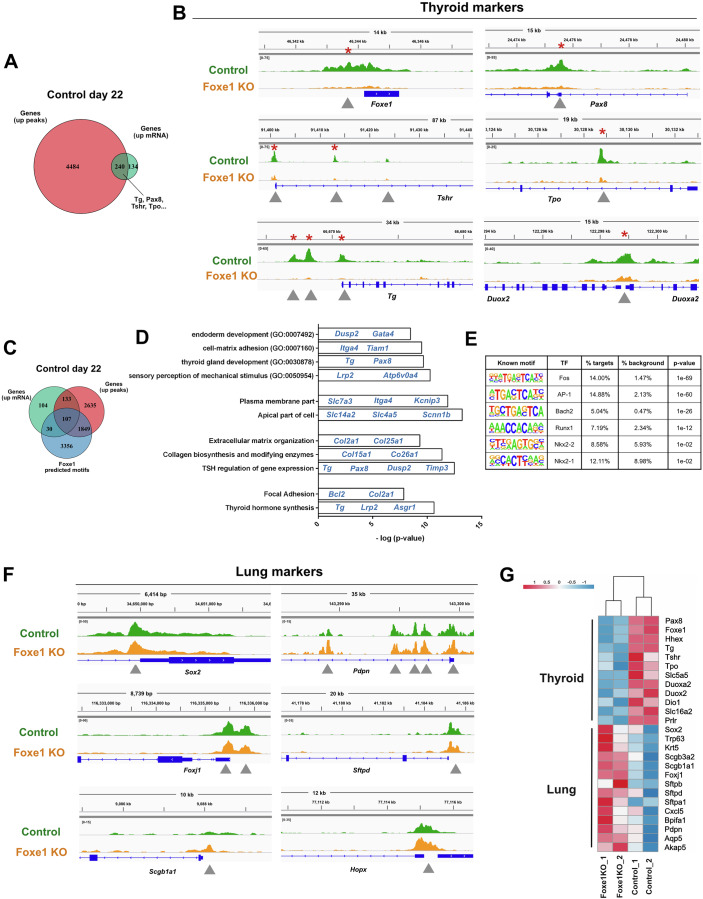
ATAC-seq profiling of Nkx2-1 expressing cells in control and Foxe1KO at day 22. (**A**) Venn diagram between the genes in which the chromatin is significantly more open and the transcripts are upregulated in control versus Foxe1KO at day 22. (**B**) Illustration of open chromatin regions at day 22 in thyroid marker genes. (**C**) Venn diagram showing 107 genes containing Foxe1 motif also being more significantly open in ATAC-seq and with transcripts upregulated in control cells at day 22. (**D**) Gene Ontology (GO) and pathway functional analysis performed with 107 genes shown in (**D**). Biological Process GO, Jensen compartments, Bioplanet, and KEGG significant terms are shown in the image. (**E**) Known motifs enriched in 793 peaks more open in Foxe1KO versus control at day 22; log2foldchange ≥ 0.58, FDR ≤ 0.05. (**F**) Illustration of open chromatin regions at day 22 in lung marker genes. (**G**) Heatmap of log-2 transformed normalized counts from bulk RNA-seq of sorted Nkx2-1+ cells at day 22. Relevant markers for thyroid and lung lineage are shown in control and Foxe1KO samples.

Our results suggest that the most striking differences in chromatin accessibility between control and Foxe1KO are seen toward the end of the protocol (Fig. 5G–I). Using the Diffbind package for differential binding analyses between control and Foxe1KO conditions, we identified more than 23547 genomic regions that are differentially accessible at day 17 (19112 are upregulated in control *vs*. Foxe1KO and 4435 up in Foxe1KO *vs*. control) (Fig. 5H) and 14293 peaks at day 22 (13,500 upregulated in control *vs*. Foxe1KO and 793 up in Foxe1KO *vs*. control) (Fig. 5I). The number of opened chromatin regions in control cells is 17-fold greater than in Foxe1KO cells at day 22, indicating an important role for Foxe1 in opening specific chromatin domains that are otherwise silenced. Of note, more than 60% of the upregulated transcripts found in bulk RNAseq analyses of the control line were associated with significant chromatin opening compared with Foxe1KO (Fig. 6A). Among them are most of the genes associated with the thyroid gland, including essential genes for gland maturation and function, such as *Tshr*, *Tpo*, and *Duox2* (Fig. 6B).

To reveal putative chromatin regions regulated by Foxe1, we applied a HOMER custom motif search based on the human Foxe1 binding motif, from the Jaspar database, on total peaks of day 22 control ATAC-seq datasets (Fig. 6C,D). We identified 107 genes, containing predicted Foxe1 binding motif, which are highly transcribed and located in the neighborhood of significantly upregulated ATAC-seq peaks in the control condition (Fig. 6C; Dataset EV1). These included genes related to thyroid development, hormone synthesis, and Tsh regulation, such as *Pax8*, *Tg*, *Dusp2*, *Timp3*, and *Asgr1*, as well as various molecular players involved in endoderm development, thyroid cell polarization, and follicle organization (Fig. 6D; Dataset EV1). Overall, our ATAC-seq results once again demonstrate the essential role of Foxe1 during thyroid morphogenesis and function and, additionally, provided the identification of a number of potential Foxe1 targets. In addition, the identification of a predicted Foxe1 binding site within regulatory regions of the Pax8 locus, together with the reduced chromatin accessibility observed upon Foxe1 loss, suggests a novel regulatory layer linking these thyroid transcription factors.

### Cis-regulatory regions around lung-related genes are equally accessible in control and Foxe1KO Nkx2-1+ cells

To investigate the gene regulatory networks enriched in Foxe1KO cells that may drive lung fate, we performed motif enrichment analysis for peaks enriched in Foxe1KO compared to control samples at day 22. We identified 793 peaks that were upregulated in Foxe1KO compared with control samples. Analysis of the HOMER motif search revealed enrichment with Fos, AP-1, and Bach2 motifs, factors involved in apoptotic cell death, hypoxia, and oxidative stress (Fig. 6E) (Machado et al, 2021; Shaulian and Karin, 2002; Zhou et al, 2016). These findings suggest that, in addition to regulating thyroid differentiation genes, Foxe1 may also contribute to the survival and stability of thyroid progenitors.

A notable finding, however, was that none of the Foxe1KO upregulated peaks found on ATAC-seq analysis were directly associated with classical markers of differentiated lung cells. Inspection of IGV tracks further revealed that many cis-regulatory regions associated with lung lineage genes display similar levels of chromatin accessibility in both control and Foxe1KO cells (Fig. 6F). Despite this comparable chromatin accessibility, RNA-seq analysis of sorted Nkx2-1⁺ cells showed increased expression of several lung-associated genes in Foxe1KO conditions (Fig. 6G). Together, these results suggest that lung-related regulatory regions are already in a permissive chromatin state in Nkx2-1⁺ progenitors, and that in the absence of Foxe1, combined with impaired thyroid commitment, these cells can activate a lung differentiation program and redirect their differentiation toward a lung epithelial fate.

## Discussion

In the present study, we used our stem cell-based thyroid organoid model to investigate the role of Foxe1 during early thyroid lineage specification and maturation. Our results confirm that Foxe1 is required for efficient thyroid cell commitment and plays a critical role in the maturation of thyroid follicular cells in vitro. In the presence of Foxe1, chromatin accessibility analyses revealed multiple open regulatory regions containing predicted Foxe1 binding motifs associated with key thyroid genes, including *Pax8* and *Tg*, highlighting potential regulatory elements involved in normal thyroid development and function. In contrast, Foxe1 loss led to a profound disruption of the thyroid transcriptional program. Although Nkx2-1 expression was still detected, the number of Nkx2-1⁺/Pax8⁺ double-positive cells was markedly reduced, indicating a collapse of thyroid lineage commitment. Under these conditions, a larger fraction of Nkx2-1⁺ cells remained transcriptionally permissive and capable of engaging alternative Nkx2-1-dependent programs. Consistent with this idea, we observed that cis-regulatory regions associated with lung genes were already accessible in Nkx2-1⁺ progenitors, allowing Nkx2-1 to cooperate with lung-associated cofactors and promote activation of a lung epithelial differentiation program, ultimately leading to the formation of lung-like organoids in vitro.

### Foxe1 role in thyroid generation in vitro

Early thyroid development depends on the correct expression of the four major transcription factors, Nkx2-1, Pax8, Hhex, and Foxe1, in the thyroid primordium. Their expression is responsible for the activation of the gene regulatory network leading to thyroid differentiation and maintenance of the differentiation status in adulthood (De Felice and Di Lauro, 2004; Lim et al, 2022; López-Márquez et al, 2021). Regarding Foxe1, Di lauro and coworkers have shown that although thyroid anlage is specified in the absence of Foxe1, Foxe1-null embryos exhibit severe defects in thyroid morphogenesis as early as E9.5, leading to complete disappearance of thyroid tissue around E11.5 or persistence of a small, misplaced gland (De Felice et al, 1998). In humans, homozygous mutations in Foxe1 loci lead to congenital hypothyroidism, due to severe hypoplasia or complete agenesis of the thyroid gland (Clifton-Bligh et al, 1998). Our results support the intrinsic role of Foxe1 in early thyroid development, since in the absence of Foxe1, the efficiency in deriving thyroid cells in vitro is drastically impaired. Moreover, the few thyroid follicular cells that differentiate in the absence of Foxe1 are abnormal and lack signs of proper thyroid maturation, as evidenced by the lack of *Nis* expression and Tg iodination. Foxe1 is thought to act downstream of Nkx2-1, Pax8, and Hhex during thyroid development, as the onset of Foxe1 expression occurs through the direct action of Pax8, suggesting a hierarchy among thyroid transcription factors (López-Márquez et al, 2021; Parlato et al, 2004). Indeed, we have shown that transient induction of mESCs with Nkx2-1/Pax8 is sufficient to induce Foxe1 expression in vitro (Antonica et al, 2012). The fact that Foxe1 acts downstream of Nkx2-1, Pax8, and Hhex during thyroid development may explain why a small number of follicle-like cells can still form in its absence, albeit scarce and non-functional. Interestingly, our results reveal an additional layer of transcriptional co-regulation: while Pax8 initiates Foxe1 expression during early thyroid specification, Foxe1 in turn becomes necessary later to maintain chromatin accessibility at the Pax8 locus, thereby reinforcing thyroid lineage commitment. This finding suggests that Foxe1 not only acts as an effector of the thyroid transcriptional hierarchy but also contributes to stabilizing the regulatory network, ensuring sustained Pax8 activity and proper thyroid differentiation.

One of the proposed mechanisms of Foxe1 action on thyroid morphogenesis is the direct regulation of genes critical for thyroid maturation and function. In differentiated thyroid follicular cells, Foxe1 is involved in TSH-mediated regulation of *Tg* and *Tpo* expression, and Foxe1 binding sites have been described in the promoters of these genes (Aza-Blanc et al, 1993; Francis-Lang et al, 1992; López-Márquez et al, 2019; Santisteban et al, 1992). Foxe1 can also bind directly to cis-regulatory regions of Nis/Slc5a5 and Duox2 (Fernández et al, 2013). In the present work, we show that the expression of many thyroid functional genes is impaired in the absence of Foxe1, confirming these previous reports. No cells expressing *Nis*, *Tpo*, and *Duox2* were identified by scRNAseq analyses of Foxe1KO cells. Integrated bulk ATAC/RNA-seq analyses showed that a reduction in the expression of genes involved in thyroid maturation might be associated with silencing of many cis-regulatory regions, including at their promoters. Interestingly, there are 17-fold more open chromatin regions in Nkx2-1+ control cells compared with Foxe1KO/Nkx2-1+ cells, indirectly supporting the view that Foxe1 has an impact on chromatin remodeling in thyrocytes. Although our experiments were not designed to explore this aspect, the function of Foxe1 as a pioneer factor, well described for other members of the Forkhead family of transcription factors (Zaret and Carroll, 2011), has previously been suggested due to its ability to bind the compacted chromatin around the inactive *Tpo* promoter (Cuesta et al, 2007).

In addition, we identified novel genomic targets of Foxe1 by direct comparison analyses between Foxe1-depleted and control Nkx2-1-expressing cells. In silico prediction of Foxe1 binding motifs in upregulated genes at the level of RNA expression and chromatin accessibility revealed 107 potential targets of Foxe1, including *Pax8* and *Tg* (Francis-Lang et al, 1992). Other targets identified included genes involved in proper thyroid function and TSH receptor activity or deregulated in thyroid cancer, such as *Lrp2/Megalin* (Marinò and McCluskey, 2000), *DREAM/Kcnip3* (Andrea et al, 2005), *Timp3* (Zarkesh et al, 2018), *Slc26a7* (Dom et al, 2021) and *Bcl-2* (Fagman et al, 2011; Porreca et al, 2012). Moreover, Foxe1 binding motifs were also found in genes related to the maintenance of follicle structure, the expression of various membrane-bound transporters, and the regulation of cell-matrix adhesion. In summary, the present study sheds light on novel molecular players essential for thyroid development and homeostasis. Ultimately, our thyroid stem cell-derived organoid system proved to be a powerful model for such analyses because, it is an in vitro model, so obtaining an adequate amount of cells is not as limited as in vivo; growing differentiated thyrocytes in 3D, to generate polarized follicular structures, allows the correct localization and signaling of factors involved in the thyroid hormone synthesis machinery, and therefore the screening of new modulators of thyroid function may be more representative.

### Lung generation in vitro, in the absence of Foxe1

Besides the disruption of thyroid lineage formation in Foxe1KO cells, we additionally observed the appearance of Nkx2-1+ organoids that lack Pax8 expression and are morphologically distinct from our stem cell-derived thyroid follicles. Considering that Nkx2-1, in endoderm-derived tissues, is also present in embryonic and adult lung cells (Herriges and Morrisey, 2014; Lazzaro et al, 1991; Nikolić et al, 2018), we hypothesize that permissive signals for lung differentiation occurred in the context of Foxe1 depletion. Indeed, these Nkx2-1+ organoids possess numerous markers of airway cell types. For example, basal lung cells can be identified in Nkx2-1⁺/Krt5⁺/Sox2⁺/Trp63⁺ cystic organoids, while other airway-like organoids form branched structures composed of a Sox2⁺ single-layered epithelium containing goblet-like cells that secrete mucus into the lumen. Single-cell RNA sequencing further revealed the presence of alveolar-like cells; however, our findings suggest that these cells remain largely immature, consistent with the developing alveolar sacs observed by ultrastructural analyses. Overall, in the absence of Foxe1, a large subset of mESCs can divert to a lung differentiation program, generating a mixture of lung-derived cell types, with airway fates predominating. Despite the clear lung phenotype in Foxe1KO conditions, the resulting organoids do not fully recapitulate in vivo lung development, containing only a subset of cell types with incomplete maturation and proportions, likely reflecting limitations of the current culture conditions for full lung development.

Nkx2-1 is the earliest known marker of respiratory fate. As thyroid, future lung progenitors are derived from the ventral anterior foregut endoderm, posterior to the thyroid field, and the onset of lung differentiation can be detected by the expression of Nkx2-1 (Cardoso and Lu, 2006; Lazzaro et al, 1991). We speculate that in our current model, direct overexpression of Nkx2-1 (and Pax8) without stepwise lineage restriction allows a subset of mESCs to activate a latent lung differentiation program that is normally quiescent under control conditions. The absence of Foxe1, a key factor for thyroid specification, likely creates a permissive state that enables these cells to commit to and expand along a lung fate. Consistently, our previous scRNA-seq analysis of mouse thyroid organoids revealed a small population of Nkx2-1⁺/Krt5⁺ epithelial cells, suggesting that even in Foxe1-expressing organoids, a minor subset of cells may adopt a lung-like identity (Romitti et al, 2021). Our study supports the hypothesis of a certain level of diversity of early Nkx2-1 progenitors generated in vitro. Interestingly, an elegant study has recently demonstrated that a subset of NKX2-1+ progenitors, derived from human pluripotent stem cells, can generate alternative, non-lung endodermal cell fates (Hurley et al, 2020).

It is important to point out that our bulk ATAC-seq analyses suggest that at the level of chromatin accessibility, the main differences between Foxe1KO and control Nkx2-1+ cells are mainly due to an enrichment of a thyroid differentiation program towards the end of the differentiation protocol: cis-regulatory regions of genes such as *Tg*, *Tshr*, *Tpo*, *Duox2*, and *Duoxa2* are significantly more open in control versus Foxe1KO-Nkx2-1+ cells. On the other hand, the chromatin state around genomic locations of lung-typical genes, including promoter regions, appears to be equally open in both conditions, even if these genes are more expressed in Foxe1KO Nkx2-1+ cells. Two hypotheses can be inferred from these results: (1) Many known lung markers, such as *Sftpb*, *Scgb1a1*, *Napsa*, *Ager*, *Aqp5*, and *Sox9*, are also expressed to some extent in thyroid, so it makes sense that chromatin accessibility does not differ substantially between these cells for those genes (Dame et al, 2017; Silberschmidt et al, 2011; and our scRNAseq). Further differential analyses across mouse development are needed to better understand the role of these genes in a thyroid context (Ikonomou et al, 2020; Mou et al, 2012). (2) By default, mESC-derived Nkx2-1 cells may have some potential to give rise to lung cells. Depletion in Foxe1 expression would allow this potential to be unleashed. It is interesting to see that, in our results, the global chromatin accessibility of in vitro Nkx2-1 progenitor cells is very similar at early stages (day 10), suggesting a direct role of transient overexpression of Nkx2-1 and Pax8 in triggering both thyroid/lung programs. Such an event of concomitant generation of two endodermal lineages has been demonstrated earlier in the direct lineage conversion of fibroblasts to liver and large intestine cells, following overexpression of Foxa1 (Morris et al, 2014).

**Figure EV8:**
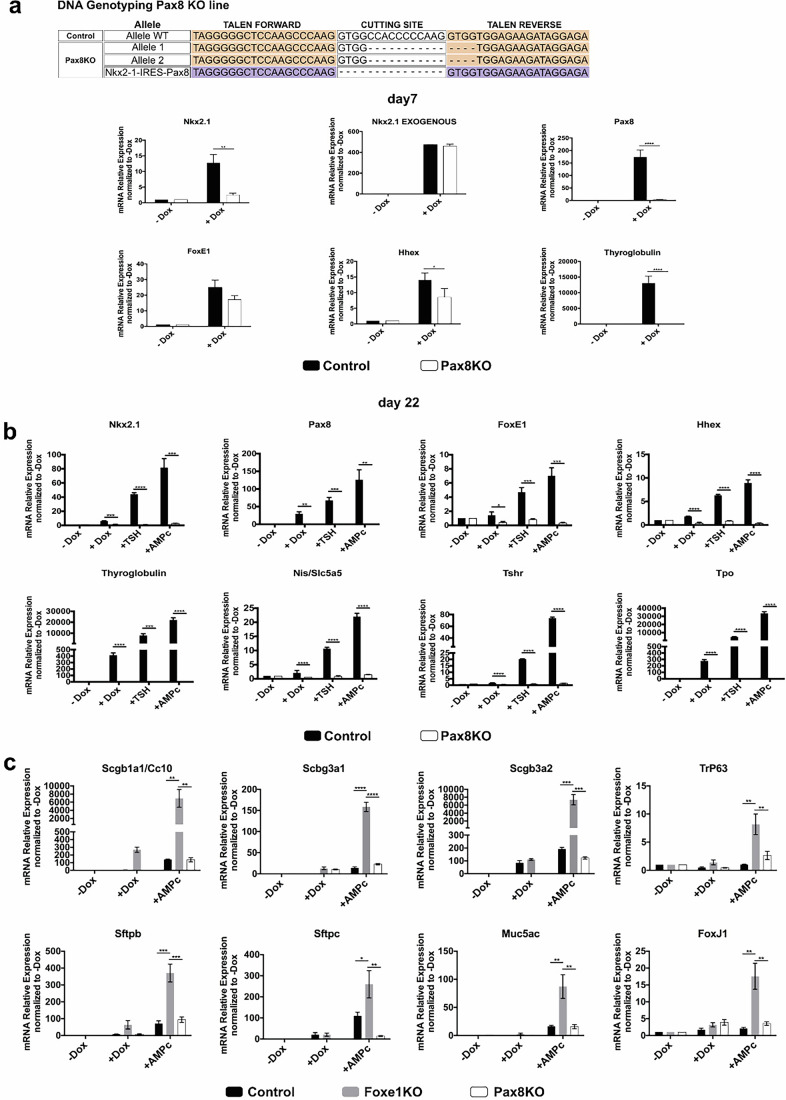
Invalidation of Pax8 impairs thyroid formation but is not sufficient to drive lung differentiation. (**A**) Genomic profiling of Pax8KO mESCs obtained by TALEN technology. (**B**) Expression of endogenous thyroid markers for control and Pax8KO differentiated cells after Dox-mediated induction of Nkx2.1-Pax8 during 3 days (day 4–day 7) followed by 8-br-cAMP treatment until day 22. A robust downregulation of all thyroid genes is observed in Pax8KO differentiated cells compared to controls. (**C**) Expression of endogenous lung markers in control, Foxe1KO, and Pax8KO differentiated cells following the same differentiation protocol. Expression of lung-related genes are not induced in cells of Pax8KO line. Relative expression of each transcript is presented as fold change compared to untreated cells (-Dox) at day 7 (mean +/−SEM; *n* = 3 biological replicates). Unpaired *t* test was used for statistical analysis. *P < 0.05, **P < 0.01, ***P < 0.001.

Finally, it is important to note that, although Pax8 is also forcibly expressed via doxycycline in our model, we do not consider it to inhibit lung fate induction in our model, even though Pax8 is not expressed in the lung during mouse embryogenesis (Kurmann et al, 2015; Mou et al, 2012). Pax8 is rapidly downregulated in Foxe1KO cells, while Nkx2-1 expression, albeit less pronounced than under control conditions, is maintained throughout the protocol. Moreover, no derivation of thyroid or lung is achieved in Pax8KO mESC lines (Fig. EV8A–C). In other words, the simple removal of another key transcription factor for thyroid differentiation did not result in the appearance of lung instead of thyroid, as is the case in Foxe1. Nevertheless, we speculate that in the case of Pax8 loss-of-function in mESCs, activation of high endogenous Nkx2-1 levels is less successful, suggesting that Pax8, in our model (Antonica et al, 2012), helps to regulate Nkx2-1 expression in the first days, and thus a later Pax8 downregulation might create a permissive environment for the acquisition of a lung program in vitro. Interestingly, in our recent publication, which describes the first protocol to obtain functional thyroid follicles derived from human ES cells by forward programming (Romitti et al, 2022), a small cell population expressing markers of airway cells could be identified, supporting the view that overexpressing Nkx2-1 in ES cells may lead to the in vitro specification of both lineages, in both mouse and human ES cells.

In conclusion, the present work advances our understanding of the critical role of Foxe1 in initiating and sustaining proper thyroid tissue formation and function, while also highlighting novel molecular players for future investigation in thyroid biology. Beyond the thyroid, our findings underscore the intricate relationships among endodermal lineages during differentiation, particularly between thyroid and lung. Supporting this concept, in vivo studies by Fagman et al (2004) showed that loss of Shh signaling during early organogenesis leads to thyroid dysgenesis and the appearance of aberrant thyrocytes expressing Nkx2-1, Foxe1, and Tg in the presumptive trachea, emphasizing the need to repress inappropriate thyroid programs in non-thyroid anterior foregut endoderm (Fagman et al, 2004). Building on this, it is intriguing to speculate that transient thyroid/lung bipotent progenitors may exist in vivo, analogous to the transient bipotent progenitors described during liver and pancreas development (Deutsch et al, 2001; Xu et al, 2011). Future studies using lineage tracing approaches could directly test the existence and fate of such progenitors, providing a deeper understanding of early endodermal plasticity and the mechanisms that safeguard lineage fidelity.

## Methods

**Table:** Reagents and tools table

Reagent/resource	Reference or source	Identifier or catalog number
**Experimental models**		
A2Lox.Cre_TRE-Nkx2-1/Pax8_Tg-EGFP mESC line	Antonica et al, 2012	n/a
Foxe1KO/A2Lox.Cre_TRE-Nkx2-1/Pax8_Tg-EGFP mESC line	This study	n/a
A2Lox.Cre_TRE-Nkx2-1/Pax8_Tg-EGFP_Nkx2-1-T2A-mKO2 mESCs line	This study	n/a
Foxe1KO/A2Lox.Cre_TRE-Nkx2-1/Pax8_Tg-EGFP_Nkx2-1-T2A-mKO2 mESCs line	This study	n/a
**Recombinant DNA**		
pU-BbsI-T2A-Cas9-BFP	Addgene	64323
pUC57-mNkx2-1-T2A-mKO2-pGK-Puro	This study	n/a
pSalk-Cre plasmid	Gift from Dr Kyba	n/a
pTP53-TALEN-GFP reporter	This study	n/a
**Antibodies**		
Nkx2-1	Abcam	ab76013
Nkx2-1	Invitrogen	MA5-13961
Pax8	Cell Signaling	59019S
Tg	DAKO	A0251
Ecadh	BD	610181
ZO-1	Invitrogen	339100
Nis	Gift from N. Carrasco	n/a
Tg-I	Gift from C. Ris-Stalpers	n/a
Sox2	R&D	2018
Krt5	Covance	905901
P63	Abcam	ab735
Muc5ac	Abcam	ab3649
Scgb3a2	Gift from S. Kimura	
Foxe1	Biopat	PA0200
Kusabira Orange 2	MBL Life Science	PM051M
Kusabira Orange 2	MBL Life Science	M168-3M
Pdpn	Proteintech	11629-1-AP
Hopx	Proteintech	11419-1-AP
CC10/Scgb1a1	Santa Cruz Biotechnology	sc9772
Donkey anti-mouse IgG Cy3-conjugated	Jackson Immunoresearch	715-165-150, RRID:AB_2340813
Donkey anti-rabbit IgG Cy3-conjugated	Jackson Immunoresearch	711-165-152, RRID:AB_2307443
Donkey anti-goat IgG Cy3-conjugated	Jackson Immunoresearch	705-165-147, RRID:AB_2307351
Donkey anti-mouse IgG Alexa Fluor 488-conjugated	Jackson Immunoresearch	715-545-150, RRID:AB_2340846
Donkey anti-mouse IgG Alexa Fluor 647-conjugated	Jackson Immunoresearch	715-605-150, RRID:AB_2340862
Donkey anti-rabbit IgG Alexa Fluor 647-conjugated	Jackson Immunoresearch	711-605-152, RRID:AB_2492288
**Oligonucleotides and other sequence-based reagents**		
**Primer sequences**		
β2m-Globulin	Eurogentec	F: GCTTCAGTCGTCAGCATGG R: CAGTTCAGTATGTTCGGCTTCC
Nkx2-1 (exogenous)	Eurogentec	F: GGCGCCATGTCTTGTTCT R: ACACCGGCCTTATTCCAAG
Nkx2-1	Eurogentec	F: GGCGCCATGTCTTGTTCT R: GGGCTCAAGCGCATCTCA
Pax8	Eurogentec	F: CAGCCTGCTGAGTTCTCCAT R: CTGTCTCAGGCCAAGTCCTC
Foxe1	Eurogentec	F: GGCGGCATCTACAAGTTCAT R: GGATCTTGAGGAAGCAGTCG
Hhex	Eurogentec	F: AAGTGAGGTTCTCCAACGACC R: CATTTAGCTCGGCGATTCTGAA
Tg	Eurogentec	F: GTCCAATGCCAAAATGATGGTC R: GAGAGCATCGGTGCTGTTAAT
Nis/Slc5a5	Eurogentec	F: AGCTGCCAACACTTCCAGAG R: GATGAGAGCACCACAAAGCA
Tshr	Eurogentec	F: GTCTGCCCAATATTTCCAGGATCTA R: GATGAGAGCACCACAAAGCA
Tpo	Eurogentec	F: ACAGTCACAGTTCTCCACGGATG R: ATCTCTATTGTTGCACGCCCC
Mct8/Slc16a2	Eurogentec	F: GAGTTCCAAGCAGCATGGGT R: ATAGGTGAAGTAGCGCAGGC
Sox2	Eurogentec	F: CACAACTCGGAGATCAGCAA R: CTCCGGGAAGCGTGTACTTA
Scgb1a1	Eurogentec	F: ATCTGCTGCAGCTCAGCTTCTT R: AAAGGCTTCAGGGATGCCACAT
Scgb3a1	Eurogentec	F: GATGGCCAAGTGGCTTAATG R: TCTGTGTGGCTCTGCTCAGT
Scgb3a2	Eurogentec	F: ACAGGGAGACGGTTGATGAG R: AGTCCCGGAAAACATCACAG
Trp63	Eurogentec	F: AAACCAGAGATGGGCAAGTCCT R: TTTGCGCTGTCCGATACTTGCT
Sftpb	Eurogentec	F: GAACTCTGATCAAGCGGGTT R: TGCGTCTAGCAGGAGAACTG
Sftpc	Eurogentec	F: GAGAAACCTTACAAAATGGACA R: AGCAGAGCCCCTACAAT
Muc5ac	Eurogentec	F: TGCCGCGTCAATGGAAAGTTGT R: TACAGACACAGGCACCAGCATT
Foxj1	Eurogentec	F: ACAACTTCTGCTACTTCCGCCA R: TTCTCCCGAGGCACTTTGATGA
Aqp5	Eurogentec	F: TGCGCTCAGCAACAACACAACA R: TTCATGGAACAGCCGGTGAAGT
**TALEN sequences**		
Foxe1	Eurogentec	F: TTCCCGTTCTACCGCGACAA R: TGAGGTTGTGGCGGATGCTG
Pax8	Eurogentec	F: TAGGGGGCTCCAAGCCCAAG R: GTGGTGGAGAAGATAGGAGA
**Single-guide RNA sequences**		
Nkx2-1 3’-UTR guide	Eurogentec	GGAAGCGTTGAGGTCGCGCG
Foxe1 sgRNA 1	Eurogentec	CTTCCTCAAGATCCCGCGCG
Foxe1 sgRNA 2	Eurogentec	TAGCCCGCATAGACGGCGCC
**Chemicals, enzymes, and other reagents**		
Matrigel	BD	354230
DPBS, no calcium, no magnesium	Gibco	14190144
DMEM	Gibco	11966025
MEM-Non-Essential Amino Acids (MEM-NEAA) (100 X)	Gibco	11140035
Sodium pyruvate (100 mM)	Gibco	11360070
Penicillin-Streptomycin (10,000 U/mL)	Gibco	15140163
2-Mercaptoethanol	Sigma-Aldrich	M6250
L-Ascorbic acid	Sigma-Aldrich	A4403
Calcein Violet	Thermo Fisher Scientific	65-0854-39
hrTSH (Thyrogen)	Genzyme	NDC58468-1849-2
8-br-cAMP	Biolog	B007
Lipofectamine 3000	Thermo Fisher Scientific	L3000008
Fetal bovine serum (FBS)	Gibco	10270106
ES-qualified FBS	Millipore	ES-009-B
Puromycin	Sigma-Aldrich	P4512
Q5 High-Fidelity Taq Polymerase	New England Biolabs	M0491S
Zero Blunt® PCR Cloning Kit	Thermo Fisher Scientific	K2700-20
RNeasy micro kit	Qiagen	Cat# 74004
SuperScript™ II Reverse Transcriptase	Thermo Fisher Scientific	Cat# 18064014
Takyon™ No ROX SYBR 2X MasterMix blue dTTP	Eurogentec	Cat# UF-NSMT-B0701
Glutaraldehyde	Sigma-Aldrich	G5882
Methimazole	Sigma-Aldrich	M8506
^125^I	PerkinElmer	NEZ033001MC
γ-globulins	Sigma-Aldrich	G5009
Formaldehyde	Sigma-Aldrich	F8775
Bovine serum albumin	Sigma-Aldrich	Cat# A3294
Triton™ X-100	Sigma-Aldrich	T8787
Tween20	Sigma-Aldrich	P1379
Horse serum	Sigma-Aldrich	H1270
DAPI	Thermo Fisher Scientific	62248
Glycergel	Dako	C0563
Qiazol lysis reagent	Qiagen	Cat# 79306
Collagenase type IV	Sigma-Aldrich	Cat# C9891
Dispase II	Roche	Cat# 4942078001
TripLE Express	Gibco	Cat# 12605010
HBSS, calcium, magnesium, no phenol red	Gibco	Cat# 14025050
Ovation Solo RNA-seq	NuGEN Technologies	0500
RNA 6000 Nano Kit	Agilent	3822190
DNA 1000 kit.	Agilent	5067-1504
Quant-iT PicoGreen kit	Thermo Fisher Scientific	P11495
Chromium Next GEM Cell 3’ GEM, Library & Gel Bead Kit v3.1	10X Genomics	PN-1000121
Chromium Next GEM Chip G Single Cell kit	10X Genomics	PN-1000127
Single Index Kit T Seat A	10X Genomics	Cat # PN-1000213
Nextera DNA Library Prep kit	Illumina	FC-121-1030
MinElute Reaction Cleanup	Qiagen	28204
**Software**		
FACSDiva software	BD Biosciences	http://www.bdbiosciences.com/instruments/software/facsdiva/index.jsp RRID:SCR_001456
CFX Manager	Bio-Rad	http://www.bio-rad.com/en-eh/product/cfx-manager-software RRID:SCR_017251
Black Zen software	Zeiss	http://stmichaelshospitalresearch.ca/wp-content/uploads/2015/09/ZEN-Black-Quick-Guide.pdf RRID:SCR_018163
Leica Application Suite X	Leica	https://www.leica-microsystems.com/products/microscope-software/details/product/leica-las-x-ls/ RRID:SCR_013673
GraphPad Prism 9	GraphPad	http://www.graphpad.com/ RRID:SCR_002798
CRISPOR webtool	Concordet and Haeussler, 2018	http://crispor.tefor.net/
Trimmomatic software	Bolger et al, 2014	http://www.usadellab.org/cms/index.php?page=trimmomatic RRID:SCR_011848
Hisat2 software	Kim et al, 2015	http://ccb.jhu.edu/software/hisat2/index.shtml RRID:SCR_015530
HTSeq software	Anders et al, 2015	http://htseq.readthedocs.io/en/release_0.9.1/ RRID:SCR_005514
iDEP version 0.92	Ge et al, 2018	http://bioinformatics.sdstate.edu/idep92/
Enrichr	Kuleshov et al, 2016	n/a
Cell Ranger Software	10x genomics	https://support.10xgenomics.com/single-cell-gene-expression/software/pipelines/latest/what-is-cell-ranger RRID:SCR_017344
R Seurat package (version 3.2.0)	Stuart et al, 2019	http://seurat.r-forge.r-project.org/ RRID:SCR_007322
Galaxy	Afgan et al, 2018	use.galaxu.eu
Trim Galore		https://www.bioinformatics.babraham.ac.uk/projects/trim_galore/
Bowtie 2 v.2.3.4.3	Langmead et al, 2012	n/a
MACS2 v.2.1.1.20160309.6	Zhang et al, 2008	n/a
DiffBind R package version 2.16.2		https://www.bioconductor.org/packages//2.11/bioc/html/DiffBind.html
HOMER	Heinz et al, 2010	n/a
Jasper database	Castro-Mondragon et al, 2021	n/a
Affinity Designer v2.6.5	Serif Europe	https://www.affinity.studio/fr_fr/graphic-design-software
Fiji software	Schindelin et al, 2012	https://imagej.net/software/fiji/
Inkscape v1.4.2		https://inkscape.org/release/inkscape-1.4.2/windows/64-bit/msi/dl/
**Other**		
E17.5 mouse lung scRNAseq dataset	Frank et al, 2019	n/a

### mESCs culture and differentiation

All mESC lines used in the present paper are derived from the A2Lox.Cre_TRE-Nkx2-1/Pax8_Tg-EGFP mESCs line previously generated by our group (Romitti et al, 2021). Maintenance and differentiation of mESCs lines were performed as previously described (Antonica et al, 2012, 2017).

#### Thyroid organoids differentiation

1. Culture mESCs on a feeder-layer of irradiated mouse embryonic fibroblasts, in maintenance medium.
2. For the differentiation protocol, isolate mESCs from feeder-layer cells, count and culture in hanging drops (1000 cells per drop) for the generation of embryoid bodies (day 0). Four days later, collect the embryoid bodies, embed in growth factor-reduced Matrigel, and re-plate 50 µl Matrigel drops into 12-well plates.
3. Culture MTG-embedded embryoid bodies using the differentiation medium supplemented with 1 mg/ml Doxycycline (for induction of exogenous Nkx2-1 and Pax8 transgenes) for 3 days (day 4–day 7), followed by 2 weeks of treatment (day 7–day 22) with 1 mU/ml hrTSH or 300 µM 8-br-cAMP, as indicated.

### Generation of mESC lines by TALEN technology

TALEN technology was used to edit the A2Lox.Cre_TRE-Nkx2-1/Pax8_Tg-EGFP mESC line to generate FoxE1KO cells. TALENs synthesis was performed by using Golden Gate Technology (Weber et al, 2011). mESCs were seeded into 10 cm petri dishes (150.000 cells/petri) and, in the next day, transfected with 7 µg of each TALEN-encoding plasmid (designed to target the Forkhead domain) and 7 µg of a fluorescent surrogate reporter plasmid allowing enrichment of cells with nuclease-induced mutations (GFP reporter Assay) (Ma et al, 2013). Cell transfection was performed with Lipofectamine 3000 according to the manufacturer’s instructions. 48 h later, cells were trypsinized and resuspended in PBS containing 2% of embryonic-stem-certified fetal bovine serum for cell sorting (FACS Aria and FACSDiva Software). GFP+ cells were seeded in 96-well plates in order to have 1 GFP+ cell per well. Individual colonies were expanded in 24-well, 12-well, or six-well plates with maintenance medium for 1 week. A second cell sorting procedure was performed in individual clones to obtain a fully pure population of mESCs, devoid of feeder-layer cells, for genomic PCR analysis. Sequences of TALEN constructs are depicted in the Reagents and Tools table.

### Generation of Nkx2-1 endogenous reporter mESC line by CRISPR/Cas9

For production of single-guide RNAs (sgRNAs) targeting the 3’-UTR region of Nkx2-1 loci, a fragment of the desired genomic mouse sequence was obtained from NCBI and used as input in the CRISPOR webtool (http://crispor.tefor.net/ (Concordet and Haeussler, 2018)). The most suitable sgRNA was chosen and checked for possible off-targets (Reagents and Tools table). Forward and reverse oligonucleotides, corresponding to the sgRNA sequence, were designed for insertion into the pU-BbsI-T2A-Cas9-BFP plasmid (Chu et al, 2015). To generate the pUC57-mNkx2-1-T2A-mKO2-pGK-Puro targeting vector, ~1000 bp of each left and right Nkx2-1 homology arms, around the predicted sgRNA cutting-site, were PCR amplified and inserted into a template plasmid, containing the T2A-mKO2 and loxP-flanked pGK-Puro selection cassette. Lipofectamine 3000 was used to transfect mESCs, according to the manufacturer’s instructions. 48 h later, cells were sorted based on BFP+ expression and seeded at the clonal level in 96-well plates, as described above. Positive selection of puromycin-resistant clones was performed by treatment with 1 µg/ml puromycin for 72 h. Integration of the desired cassette was confirmed by PCR of genomic DNA. Successful targeting was achieved in 7 among 125 screened clones. The selected clones integrated the donor template in both alleles. Excision of the loxP-flanked pGK-Puro fragment was achieved later by transfection with pSalk-Cre plasmid, followed by a series of cell cloning, expansion, and negative selection based on susceptibility to puromycin treatment. Genomic removal of the PuroR sequence was confirmed by PCR. Finally, mKO2 reporter activation and compatibility with Nkx2-1 protein expression were verified. For clarity, in the present paper, the novel line generated (i.e., A2Lox.Cre_TRE-Nkx2-1/Pax8_Tg-EGFP_Nkx2-1-T2A-mKO2 mESCs line) is named as “Nkx2-1 reporter line”.

### Generation of Foxe1KO/Nkx2-1 reporter mESC line

SgRNAs targeting mouse Foxe1 coding sequence were chosen based on suitable sgRNAs prediction from the CRISPOR website, and insertion of sgRNAs sequences into the pU-BbsI-T2A-Cas9-BFP plasmid was performed as cited above. Two sgRNAs were chosen (one targeting the Forkhead domain and the other outside), and two Foxe1KO lines, targeting distinct genomic domains of Foxe1, were produced. Nkx2-1 reporter mESC cells were transfected, BFP sorted, clonally seeded, and selected based on PCR and Sanger sequencing to detect clones containing frameshift mutations in Foxe1 loci. A second cell sorting procedure was performed in individual clones to obtain a fully pure population of mESCs, devoid of feeder-layer cells, for genomic PCR analysis.

All cell lines generated here were validated, in at least two different clones, by maintenance of pluripotency cell markers, ability to spontaneously differentiate into the three germ layers, activation of tetracycline-inducible Nkx2-1-Pax8 transgene, and reproducibility regarding in vitro differentiation. Regarding Foxe1KO/Nkx2-1 reporter lines, similar results were obtained in Foxe1KO lines derived from distinct designs. Only one is shown in the present paper.

### PCR detection of mutated clones (TALEN/CRISPR-Cas9)

Genomic DNA was extracted from individual mESCs clones, using a genomic DNA lysis buffer and procedure as described (Verma et al, 2017). PCR was performed using Q5 High-Fidelity Taq Polymerase according to the manufacturer’s instructions. 100 ng of genomic DNA samples were used in all reactions. Primer sets were used to amplify the targeting and cutting site of the designed TALENs/CRISPR-Cas9 guide RNAs. PCR products were then directly cloned into a TOPO-Blunt plasmid by using the Zero Blunt® PCR Cloning Kit.

### RNA extraction and RT–qPCR

For RNA preparation, cells were lysed in RNeasy Lysis buffer + 1% β-mercaptoethanol, and total RNA was isolated using RNeasy microRNA preparation kit according to the manufacturer’s instructions. Reverse transcription was done using Superscript II kit. RT-qPCR was performed in technical triplicate using Takyon™ No ROX SYBR 2X MasterMix and a CFX Connect Real-Time PCR System. Results are presented as linearized values normalized to the housekeeping gene β2m-Globulin and the indicated reference value (2-ΔΔCt). Moreover, relative expression of each target gene is presented as fold change compared to untreated cells (−dox condition). The gene-expression profile was confirmed in at least two different clones of each cell line. Primers used are listed in the Reagents and Tools table.

### Scanning electron microscopy

Organoids embedded in Matrigel were fixed in glutaraldehyde 2.5% overnight at 4 °C, rinsed, and embedded in agarose 4%. Sections of 300 µm were produced in a Vibratome (Leica) and post-fixed in OsO4 (2%) for 1 h. All treatments were done in 0.1 M cacodylate buffer (pH 7.2). After serial dehydration in ethanol, samples were dried at the critical point and coated with platinum by standard procedures. Observations were done in a Tecnai FEG ESEM QUANTA 200 at 30 kV, and images were acquired and processed by SIS iTEM software.

### Iodide organification assay

The iodide organification assay was performed as previously optimized for mouse thyroid organoids (Antonica et al, 2012).

1. Wash organoids with HBSS.
2. Incubate with 1 × 10⁶ cpm ^125^I/ml and 100 nM sodium iodide for 2 h at 37 °C.
3. Add 4 mM methimazole to stop TPO activity.
4. Dissociate cells with 0.1% Trypsin/1 mM EDTA for 15 min.
5. Assess ^125^I uptake by measuring total radioactivity (γ-counter).
6. Precipitate proteins with 1 mg γ-globulin + 20% TCA.
7. Centrifuge (2000 rpm, 10 min).
8. Quantity the radioactivity of protein-bound ^125^I (PBI; γ-counter);
9. Calculate iodide organification: iodide uptake/protein-bound iodine ratio.

### Immunofluorescence and immunohistochemistry

Primary and secondary antibodies information is provided in the Reagents and Tools table.

1. Fix cells in 4% formaldehyde for 30 min.
2. Wash samples with PBS (3 × 5 min).
3. Block samples for 30 min at room temperature using blocking buffer: 3% BSA, 1% horse serum, and 0.3% Triton X-100.
4. Incubate primary antibodies diluted in buffer containing: 3% BSA, 5% horse serum, and 0.1% Triton X-100 overnight at 4 °C.
5. Wash with PBS under gentle agitation (3 × 10 min).
6. Incubate secondary antibodies and DAPI diluted in buffer containing: 3% BSA, 5% horse serum, and 0.1% Triton X-100 for 1 h 30 min at RT.
7. Wash with PBS under gentle agitation (3 × 10 min).
8. Mount samples using Glycergel mounting medium.

### Flow cytometry intracellular immunostaining

Nkx2-1 reporter and unmodified mESCs were differentiated up to day 14 of the thyroid differentiation protocol and processed for intracellular flow cytometry staining as follows:

1. Digest the matrigel drops with a HBSS solution containing 10 U/ml dispase II and 125 U/ml of collagenase type 1A for 30 min at 37 °C. Centrifuge at 1400 rpm for 3 min.
2. To obtain a single-cell suspension, dissociate cells with TripLE Express for ≤15 min at 37 °C. Centrifuge at 1400 rpm for 3 min.
3. Rinse the samples with PBS and fix in 1.6% formaldehyde for 15 min at room temperature. Centrifuge at 1400 rpm for 3 min.
4. Permeabilization: Incubate the samples in 0.1% Triton in PBS for 1 min at 4 °C. Centrifuge at 1400 rpm for 3 min.
5. Blocking: Incubate the samples for 10 min in a PBS solution containing 4% horse serum + 0.5% Tween-20. Centrifuge at 1400 rpm for 3 min.
6. Primary antibodies staining: Incubate antibodies diluted 1:100 in PBS + 0.5% Tween-20 for 30 min at 4 °C. Centrifuge at 1400 rpm for 3 min.
7. Rinse the sample three times with antibody solution. Centrifuge (1400 rpm, 3 min)
8. Secondary antibodies staining: Incubate antibodies diluted 1:300 in PBS + 0.5% Tween-20 for 30 min at 4 °C. Centrifuge at 1400 rpm for 3 min.
9. Resuspend cells in 300 µl PBS + 2% FBS. Filter through a 30–40 µm cell strainer.
10. Acquire data using the LSRFortessa X-20 flow cytometer using FACSDiva software.
11. Controls: unstained cells, isotype controls, and negative controls (undifferentiated cells: “-dox control”) should be included in all experiments.

### RNA isolation and RNA-seq analysis

For preparation of bulk RNA-seq samples, Foxe1KO and control cells from Nkx2-1 reporter line were cultured following the differentiation protocol, and cell suspension was obtained as described above (“Flow cytometry intracellular immunostaining” section). Nkx2-1+ (mKO2 + ) cells were sorted (FACS Aria; BD Bioscience) at day 10 and day 22. In total, 10,000 mKO2+ cells per condition were collected directly into 700 µl of Qiazol lysis reagent, and RNA isolation was performed with miRNeasy micro kit following the manufacturer’s instructions. The quality and quantity of the resulting RNA were then tested using Bioanalyser 2100 (Agilent) and RNA 6000 Nano Kit. RNA integrity was preserved (RIN = 8.5), and no genomic DNA contamination was detected. Ovarion Solo RNA-seq Systems was employed, as indicated by the manufacturer, to produce high-quality indexed cDNA libraries, which were quantified using Quant-iT PicoGreen kit and Infinite F200 Pro plate reader (Tecan); DNA fragment size distribution was examined on 2100 Bioanalyser (Agilent) using DNA 1000 kit. Normalized and pooled indexed libraries (10 ρM) were loaded on flow cells and sequenced on the HiSeq 1500 system (Illumina) in a high-output mode using HiSeq Cluster kit v4. Approximately 10 million of 125 nt-long paired-end reads were obtained for each library. After removal of low-quality bases and Illumina adapter sequences using Trimmomatic software (Bolger et al, 2014), sequence reads were aligned against the mouse reference genome (Grcm38/mm10) using Hisat2 software with default parameters (Kim et al, 2015). Raw counts were obtained using HTSeq software (Anders et al, 2015) using Ensembl genome annotation GRCm38.87. Normalization, differential expression, and Gene Ontology analyses were performed with at least two biological replicates per sample, using the website iDEP version 0.92 (Ge et al, 2018). Additional Gene Ontology analysis and identification of statistically significant terms (*P* < 0.05) were performed with Enrichr (Kuleshov et al, 2016).

### Single-cell RNA-seq preparation and sequencing

At day 22 of the differentiation protocol, cell populations derived from Foxe1KO/Nkx2-1 reporter mESC lines were isolated for scRNAseq profiling. Culturing and preparation of cell suspension for FACS-sorting were performed as mentioned above for bulk RNA-seq. Different proportions of EGFP + , mKO2+, and mKO2- cells were sorted to guarantee representation of various cell types in the profiled sample (15%, 50%, 35%, respectively). Sorted cells were collected in PBS at a density of 800 cells/µl and diluted according to the kit’s instructions (10x Genomics Chromium Single Cell 3’ v3). In total, 12,000 cells were loaded onto a channel of the Chromium Single Cell 3′ microfluidic chip and barcoded with a 10X Chromium controller. Subsequently, RNA was reverse transcribed and amplified according to the manufacturer’s recommendations. Library preparation (e.g., fragmentation, dA tailing, adapter ligation, and indexing PCR) was performed based on 10x Genomics guidelines. Libraries were sequenced on an Illumina NovaSeq 6000 system.

### Single-cell transcriptomic data analysis

Raw sequencing data were aligned and annotated against the Grcm38/mm10 mouse reference genome, in which mKO2 and EGFP sequences were added. Cell Ranger Software (v.2.1.0), provided by 10x Genomics, was used for demultiplexing with default parameters. The raw counts generated from 10x Chromium pipeline were clustered using R Seurat package (version 3.2.0) (Stuart et al, 2019). Briefly, quality control pre-processing was performed to keep cells passing the following criteria: had between 1500 and 58,000 UMI counts, showed expression of at least 750 unique genes, and had less than 10% of UMI counts corresponding to mitochondrial genes. The remaining data was log-normalized, regressed out to remove effects of library size and enrichment of mitochondrial and cell cycle-related genes, and scaled, using *SCTransform* function. Principal component analysis (PCA) was calculated using the expression data of the most variable genes, and the first 10 principal components were used to graph-based clustering and UMAP plot visualization. Different values in the resolution variable (*FindClusters* function) were tested, and a resolution of 0.7 was used for clustering. Differentially expressed genes were computed with the *FindAllMarkers* function and used for heatmap visualizations. To better identify specifically lung cells, cells expressing *Nkx2-1* and *Epcam*, but devoid of *Thyroglobulin* and *Pax8* expression, were extracted from the original dataset, re-clustered based on variable genes, and a new UMAP visualization was obtained. Signature scores based on the expression of selected genes were calculated using *AddModuleScore* function. Gene lists can be found on Dataset EV1.

### Integrative single-cell RNAseq analysis

To identify shared cell populations among in vivo mouse lung and Foxe1KO-derived lung organoids, *Nkx2-1* + /*Epcam* + /*Tg*-/*Pax8*− cells from the Foxe1KO dataset were compared to E17.5 mouse lung (Frank et al, 2019). For this, the Seurat object of E17.5 mouse lung scRNAseq dataset was updated and subjected to a normalization and scaling process using the *SCTransform* function. For better visualization purposes, the original dataset was downsampled to 2000 cells. After downsampling, we combined both datasets in an unique object and calculated pairwise correspondences between individual cells using Integration features from R Seurat toolkit (Stuart et al, 2019). After integration, downstream analyses such as graph-based clustering and UMAP dimensionality reduction were performed as described above.

### ATAC sequencing

Biological replicates were obtained from control and Foxe1KO cells (Nkx2-1 reporter line) at different points of our differentiation protocol. 50000 Nkx2-1(mKO2 + ) cells were sorted from both lines at day 10 and immediately proceeded to sample preparation, based on the Omni-ATAC protocol (Corces et al, 2017). Cells derived from embryoid bodies before (day 4) and after doxycycline treatment (day 7) were also collected to distinguish open chromatin regions related to tetracycline-induced exogenous transgene activation. After centrifugation, cell pellets were resuspended in 50 µl of an ice-cold cell lysis buffer (0.1% Igepal, 0.1% Tween20, and 0.01% Digitonin in Omni-ATAC Resuspension buffer). After 3 min, samples were centrifuged for 15 min at 800 g and subsequently, nuclei were resuspended in 50 µl of reaction buffer (2.5 μl Tn5 transposase, 22.5 μl TD buffer, both from Nextera DNA sample preparation kit; 16.5 μl PBS, 0.5 μl 1%Digitonin, 0.5 μl 10% Tween20 and 5 μl H_2_0). Tagmentation reaction was performed for 30 min at 37 °C in a rocking plate (1000 rpm). DNA was purified using the MiniElute purification kit following the manufacturer’s instructions. DNA libraries were PCR amplified, DNA quality verified on 2100 Bioanalyser (Agilent) using DNA 1000 kit and size selected from 200 to 800 bp, following the manufacturer’s recommendations.

### ATAC-seq analysis

For the main steps of pre-processing and mapping of ATAC-seq data, a local installation of the Galaxy platform was used (use.galaxu.eu; (Afgan et al, 2018)). Briefly, adapter sequences were removed with Trim Galore, using default parameters. ATAC-seq paired-end reads were aligned to the mouse genome Grcm38/mm10 with Bowtie 2, modifying default parameters to include fragments of up 1000 bp, allowing dovetailing and using “very sensitive option” preset. Subsequently, mitochondrial genes, bad quality mapped sequences, and PCR duplicates were removed. Peak calling was performed for each sample using MACS2, with parameters setting of −*q* 0.05 and -- shift 0. Peaks from all samples were merged for downstream analysis. Scaling of bam files generated by MACS2 were performed and used for visualization of data tracks with Integrative Genomics Viewer (IGV). Moreover, files derived from MACS2 peak calling were used as input for Differential Binding Analysis using the DiffBind package in R (https://www.bioconductor.org/packages//2.11/bioc/html/DiffBind.html). Annotation of nearest genes associated with differentially regulated genomic regions was performed using HOMER (Heinz et al, 2010). Two biological replicates were used for the sample, and significant differential peaks were filtered according to these criteria: log2 fold change ≥0.58 and false discovery rate (FDR) ≤ 0.05. De novo motif search was performed using findMotifs.pl from the HOMER package with default parameters. To obtain Foxe1 enriched motifs in total ATAC-seq peaks, the human Foxe1 motif (MA1487.1) obtained on Jasper database (Castro-Mondragon et al, 2021), was used as a query in findMotifs.pl command.

### Statistical analysis

For most techniques (RT-qPCR, iodide organification, immunofluorescence, and flow cytometry), at least two different wells per condition were used in each differentiation experiment. Furthermore, at least three independent experiments were performed. Statistical significance was tested as follows: two-group comparison by unpaired *t* test and multiple-group comparison by the one-way analysis of variance test with a post-hoc Tukey’s comparison test. *P < 0.05, **P < 0.01, ***P < 0.001. Bar plots show mean ± SEM, unless otherwise indicated. GraphPad Prism version 6 was used for most analyses.

### Imaging

Fluorescence imaging was performed on a Leica DMI6000 with DFC365FX camera and a ZeissLSM510 META confocal microscope. Affinity Designer and ImageJ software (Schindelin et al, 2012) were used to adjust brightness, contrast, and picture size.

## Supplementary information

Peer Review File

Dataset EV1

Source data Fig. 1

Source data Fig. 2

Source data Fig. 5

Expanded View Figures

## Data Availability

Original data associated with this study are deposited in the NCBI Gene Expression Omnibus under accession numbers GSE182337; GSE182480; and GSE182676. scRNAseq data and gene expression profile (interactive tool) can be accessed at https://barbaraffonseca.shinyapps.io/Foxe1KO/. The source data of this paper are collected in the following database record: biostudies:S-SCDT-10_1038-S44319-026-00841-1.
