# Supplementary material for: Foxe1 deficiency impairs thyroid fate while supporting lung differentiation
Source: EMBO Rep. 2026 Jun 21;27(15):4462–92. doi: 10.1038/s44319-026-00841-1 (PMC13458771; doi:10.1038/s44319-026-00841-1)
Supplement: Supplementary file 1 [file 44319_2026_841_MOESM1_ESM.pdf]

EMBO reports

# Foxe1 deficiency impairs thyroid fate while supporting a lung differentiation

Bárbara F. Fonseca, Cindy Barbée, Sema Elif Eski, Pierre Gillotay, Daniel Monteyne, David Perez Morga, Samuel Refetoff, Sumeet Pal Singh, Sabine Costagliola, and Mírian Romitti

*Corresponding author(s): Sabine Costagliola (sabine.costagliola@ulb.be)*

**Review Timeline:**

| | |
|---|---|
| Transfer Date: | 23rd Mar 26 |
| Editorial Decision: | 12th May 26 |
| Revision Received: | 27th May 26 |
| Accepted: | 9th Jun 26 |

*Editor: Esther Schnapp*

## Transaction Report: This manuscript was transferred to EMBO Reports following peer review at Review Commons.

(Note: With the exception of the correction of typographical or spelling errors that could be a source of ambiguity, letters and reports are not edited. Depending on transfer agreements, referee reports obtained elsewhere may or may not be included in this compilation. Referee reports are anonymous unless the Referee chooses to sign their reports.)

**Review #1**

**1. Evidence, reproducibility and clarity:**

**Evidence, reproducibility and clarity (Required)**

**Summary**

The authors investigate the effect of Foxe1KO primarily on thyroid differentiation of mouse ES cells following a previously established protocol based on sequential endoderm induction, Nkx2-1/Pax8 overexpression and stimulation of the TSHR/cyclicAMP pathway. Silencing of Foxe1 expression significantly suppresses the generation of functional thyroid follicles. By single cell profiling a great number of Foxe1 targeted genes are identified, some confirmed from previous studies and some are new candidates. Embryonic bodies lacking Foxe1 instead accumulate various lung lineage cells characterized by known cell type markers, which appear to organize in lung tissue-like structures. Based on these findings, it is suggested that Foxe1 might be involved in endoderm cell fate decisions.

**Major comments**

The title and abstract hold promise that Foxe1 is also a regulator of lung development, and that Foxe1 transcriptional activity might be decisive for thyroid versus lung fate decisions. However, there are no experimental support suggesting that one and the same ES cells at a certain critical time point may switch fate from thyroid to lung (or vice versa). Since lung markers are induced in Nkx2-1/Pax8/cAMP+ ESC it is likely that "control" organoids with maintained Foxe1 expression already contain lung lineage cells, which might expand simply by clonal selection as the thyroid lineage is suppressed by subsequent Foxe1 deletion. Although authors discuss some in this direction, it is not obvious to readers without very careful reading that this possibility and explanation is feasible and should be considered and problematized. Observations that Foxe1KO did not at all influence gene expression in expanding lung-like cells are consistent with the idea that lung and thyroid specification in the model are independent phenomena, and argue against the existence of a common bipotent progenitor. If authors disagree, this issue and question should be more thoroughly discussed and argued for with more supporting experimental data than found in the current manuscript version.

**Minor comments**

What is the fraction of. Nkx2-1+ cells that organize into follicles vs lung structures? Based

on provided overview images (e.g. Figs. S1, S4) the general impression is that most cells do not form 3D-structures (i.e. do not differentiate). Please explain this and provide information in paper.

Fig. 1C: Supposed follicles are not shown in this graph. Why does not thyroglobulin accumulate in lumen (which if present would be a good means for quantification by counting follicles)? Indeed, follicles should be quantified to estimate induction success. Please also explain rounded structures in Foxe1KO image (are they distal lung buds?). Or are Control and Foxe1KO images confused in this panel?!?

Fig. 1E: text on Fig. legend is erroneously given under (F), whereas a dedicated and relevant text for (F) is missing.

Fig. 1F. Immunostaining of iodinated thyroglobulin (Tg-I) is very poor. Is it due to a bad antibody (does it work well in in vivo thyroid stainings?) or is organification simply inefficient? Again, poor content of Tg in lumen (as also suggested by Fig. S5A), it is puzzling. Or are in vitro-generated follicles leaky (i.e. do not behave as natural thyroid follicles)?

Figs. 2A-E: Comments on lung cell markers. A: E-cad is unspecific, Sox9 would better label branching morphogenesis C: co-staining for E-cad would help differentiate cell types. D: Goblet cells seem Nkx2-1 negative, please explain. E: Diffuse pattern. Are assumed club cells really Nkx2-1 pos? CC10 immunostaining might help. F: I doubt that SEM is conclusive for identification of specific (lung) cell types unless tissue architecture (e.g. proximal-distal positions) is considered for comparison to the natural branching process of the developing lung.

Line 161: Is it really "spontaneous" generation? Please rephrase.

Fig. S3A. According to Major Comment above, please explain in more detail why and how lung marker expression is evident in induced "Controls" (i.e. organoids without Foxe1KO). Is it due to parallel/independent lung and thyroid differentiation? Is phenotype of Foxa1KO rather a matter of clonal selection?

Figs. S3B-M. Scanning electron micrographs. Are these from one single (lung-like) structure imaged at different angles and magnitude or selected from multiple/different structures? If the latter, there a bias of selection that raises concern about cell identity. See similar SEM comment above.

Line 181: Text states that cells additionally were visualized by microscopy, but this is not shown in Fig. 4.

Fig. 4. Data based/biased on computationally Pax8-negative selected Foxe1KO cells. Are Pax8 negative cells present in "Control" (Foxe1+) organoids and a potential source of enrichment independent of the thyroid lineage?

The paper by Fagman et al. (Am J. Pathol, 2004), which shows aberrant/ectopic thyroid differentiation in airway respiratory epithelium in ShhKO mouse embryos, may by cited and discussed with reference to the possible existence of bipotent lung/thyroid progenitors/stem-like cells in vivo.

**2. Significance:**

**Significance (Required)**

The results are indeed of great value mainly for developmental biologist interested in regenerative medicine and specifically concerning in vitro systems for lung and thyroid differentiation. The provided single cell data sets of thyroid progenitors undergoing differentiation and the impact of Foxe1KO are a major achievement and resource.

This reviewer´s expertise is mainly in vivo thyroid development.

**3. How much time do you estimate the authors will need to complete the suggested revisions:**

**Estimated time to Complete Revisions (Required)**

**(Decision Recommendation)**

Cannot tell / Not applicable

**4. *Review Commons* values the work of reviewers and encourages them to get credit for their work. Select 'Yes' below to register your reviewing activity at [Web of Science Reviewer Recognition Service] (formerly Publons); note that the content of your review will not be visible on Web of Science.**

**Web of Science Reviewer Recognition**

No

**Review #2**

**1. Evidence, reproducibility and clarity:**

**Evidence, reproducibility and clarity (Required)**

**Summary:**

This study by Fonseca et al investigated how the specification of mouse ESCs towards thyroid lineage was regulated by the presence or absence of Foxe1, a thyroid specific transcriptional factor. Compromised thyroid induction was observed when Foxe1 was knocked out. Interestingly, the author found increased induction of lung cells in the absence of Foxe1, suggesting its role in regulating the balance of thyroid-versus-lung specification. While interesting, the main issue with this study is the lack of quantitative analysis of cellular specification, and the lack of comprehensiveness regarding the markers used to characterize each cell lineage, especially for the lung lineages.

**Major points:**

For analyzing the outcome of lineage specification in the comparison of with and without Dox or in the comparison of control versus Foxe1 KO, the only quantitative readout is qPCR. The author should perform additional characterization using flow cytometry for NKX2.1, Pax8, Tg, Tg-I, Ecad, and ZO-1 to reveal more clear mechanism: reduced number/percentage of cellular specification into thyroid lineage, or immature phenotypes in specified thyroid cells.

The authors claimed that in the absence of Foxe1, lung organoid can be observed. Quantitative analysis, such as organoid count or flow cytometry, should be provided to assess this comparing organoid identities in the presence and absence of Foxe1.

In Figure 2, the claim of lung cell identities is not well supported. (1) SEM data on alveolar and goblet cells is not conclusive; (2) Alveolar type 1 cells should be characterized by AGER and AQP5 besides HOPX; (3) Alveolar types 2 cells should be characterized by NKX2.1 and SFTPC co-staining; (4) For showing proximal lung identities, it would be helpful if the authors can co-stain more than one lineage, such as basal cell together with goblet cell/ciliated cells to reveal potential pseudostratified epithelium.

**Minor points:**

All characterization of in vitro induced thyroid cells should be accompanied by parallel analysis of native thyroid cells (from in vivo mice) that serve as a benchmark for the maturity of the induced cells. Some staining, such as Fig 1F on Tg-I remains quite different from what is reported from in vivo findings.

The labeling of panel E and F in Figure.1 should be switched.

**2. Significance:**

**Significance (Required)**

This study provided direct in vitro evidence regarding the critical role of Foxe1 for thyroid lineage induction, and suggested its role in balancing thyroid versus lung fate determination. It is thus important to the field of both thyroid and lung developmental and stem cell biology. However, the significance of this study in hindered by the lack of comprehensiveness in the analysis.

**3. How much time do you estimate the authors will need to complete the suggested revisions:**

**Estimated time to Complete Revisions (Required)**

**(Decision Recommendation)**

Between 3 and 6 months

**4. *Review Commons* values the work of reviewers and encourages them to get credit for their work. Select 'Yes' below to register your reviewing activity at Web of Science Reviewer Recognition Service (formerly Publons); note that the content of your review will not be visible on Web of Science.**

**Web of Science Reviewer Recognition**

No

**Review #3**

**1. Evidence, reproducibility and clarity:**

**Evidence, reproducibility and clarity (Required)**

Costagliola et al. have demonstrated that Foxe1, a transcription factor, plays a key role in the proper differentiation of Nkx2-1 (+) cells into thyroid follicles. They have also revealed that some Foxe1-null/Nkx2-1 (+) cells differentiate into the lung, including airway and alveolar epithelia, in their ES cell-derived organoid system. Although it has already been appreciated that Foxe1 contributes to the thyroid development in mice and humans, this excellent study has clarified that its absence, as a result, enhances the differentiation of Nkx2-1 (+) cells into the lung. I have no serious criticisms regarding methodology, results, and interpretation of results. I' d like you to elucidate whether similar findings are obtained even from human ES cell lines in the future.

**Minor comment:**

1. Fig 3C-E, Fig 6B, D, and F: These figures are so small that the words are almost illegible.

**2. Significance:**

**Significance (Required)**

I'm a pathologist who specialize in lung cancer and the stem cells in the distal airway. This paper will probably attract those who are interested in the development of the thyroid or the lung, because the authors have revealed that 1) Foxe1 contributes to the proper thyroid development, and 2) its absence consequently enhances the differentiation of Nkx2-1 (+) cells into the lung.

**3. How much time do you estimate the authors will need to complete the suggested revisions:**

**Estimated time to Complete Revisions (Required)**

**(Decision Recommendation)**

Less than 1 month

**4. *Review Commons* values the work of reviewers and encourages them to get credit for their work. Select 'Yes' below to register your reviewing activity at [Web of Science Reviewer Recognition Service] (formerly Publons); note that the content of your review will not be visible on Web of Science.**

**Web of Science Reviewer Recognition**

No

# Full Revision

**Manuscript number:** RC-2022-01578R
**Corresponding author(s):** Sabine Costagliola

## 1. General Statements

We are pleased to submit the revised version of our manuscript entitled **"Foxe1 deficiency impairs thyroid fate while supporting a lung differentiation program"** (Review Commons Refereed Preprint #RC-2022-01578R).

We are grateful for the careful and constructive evaluation provided by the reviewers. Their insightful comments have significantly strengthened the manuscript, both conceptually and experimentally.

We sincerely apologize for the delay in submitting this revision. Addressing the reviewers' comments required additional experimental work, and during this period, the postdoctoral researcher who initiated and led the project completed her training and left the laboratory, requiring a reorganization of responsibilities within the team to ensure rigorous completion of the requested studies. We appreciate your patience and believe that the manuscript has been considerably strengthened as a result.

Collectively, these modifications move the manuscript beyond a descriptive study and provide new mechanistic insight into the role of Foxe1 in thyroid specification, late chromatin regulation of Pax8 expression, and the permissive state originated in the Foxe1 absence leading to Nkx2.1 differentiation into lung.

In addition, we would like to inform you that the author order has been modified in this revised version to accurately reflect contributions made during the revision process. As a result, Mírian Romitti has been moved to co–last author. All authors have reviewed and approved this change as well as the final version of the manuscript.

We are excited to resubmit this substantially improved version and believe it now provides a clearer and more mechanistically grounded contribution to the field.

## 2. Point-by-point description of the revisions

*This section is mandatory. Please insert a point-by-point reply describing the revisions that were <u>already carried out and included</u> in the transferred manuscript.*

We would like to thank all reviewers for their constructive comments and valuable suggestions, which have helped us improve the quality and clarity of our manuscript. Below, we provide a point-by-point response to all comments. The corresponding revisions have been incorporated into the transferred manuscript, and our responses are highlighted in blue.

# Full Revision

Review COMMONS

***Reviewer #1 (Evidence, reproducibility and clarity (Required)):***

*Summary*

*The authors investigate the effect of Foxe1KO primarily on thyroid differentiation of mouse ES cells following a previously established protocol based on sequential endoderm induction, Nkx2-1/Pax8 overexpression and stimulation of the TSHR/cyclicAMP pathway. Silencing of Foxe1 expression significantly suppresses the generation of functional thyroid follicles. By single cell profiling a great number of Foxe1 targeted genes are identified, some confirmed from previous studies and some are new candidates. Embryonic bodies lacking Foxe1 instead accumulate various lung lineage cells characterized by known cell type markers, which appear to organize in lung tissue-like structures. Based on these findings, it is suggested that Foxe1 might be involved in endoderm cell fate decisions.*

*Major comments*

*The title and abstract hold promise that Foxe1 is also a regulator of lung development, and that Foxe1 transcriptional activity might be decisive for thyroid versus lung fate decisions. However, there are no experimental support suggesting that one and the same ES cells at a certain critical time point may switch fate from thyroid to lung (or vice versa). Since lung markers are induced in Nkx2-1/Pax8/cAMP+ ESC it is likely that "control" organoids with maintained Foxe1 expression already contain lung lineage cells, which might expand simply by clonal selection as the thyroid lineage is suppressed by subsequent Foxe1 deletion. Although authors discuss some in this direction, it is not obvious to readers without very careful reading that this possibility and explanation is feasible and should be considered and problematized.*

We thank the reviewer for this important and thoughtful comment. We agree that our data do not demonstrate a direct fate switch of individual ES cells from thyroid to lung identity at a defined developmental time point. We have revised the title, abstract, and discussion to clarify that our findings support a model of lineage stabilization and transcriptional competition rather than active binary fate conversion.

Our chromatin accessibility data argue against induction of a *de novo* lung program upon Foxe1 loss. In Foxe1 KO cells, we observe:

- A marked reduction in chromatin accessibility at the Pax8 locus (see Figure 6B)
- No significant gain in accessibility at canonical lung program loci (see Figure 6F)

Thus, lung gene activation does not require establishment of new accessible chromatin regions. Instead, lung-associated loci appear to be in a permissive chromatin configuration in Nkx2-1+ foregut progenitors.

Importantly, quantitative lineage analysis further supports destabilization of thyroid commitment rather than emergence of a new lineage. In wild-type organoids, approximately 80% of Nkx2-1+ cells co-express Pax8, indicating strong thyroid commitment. In contrast, in Foxe1 KO organoids, only ~20% of Nkx2-1+ cells retain Pax8 expression (see data below). This substantial reduction in Nkx2-1$^+$/Pax8$^+$ double-positive cells indicates collapse of thyroid lineage reinforcement, leaving a larger fraction of Nkx2-1-positive cells transcriptionally permissive and capable of engaging alternative Nkx2-1-dependent programs such as lung.

Mechanistically, our data support the following model:

1. Early during differentiation, Pax8 induces Foxe1 expression.
2. Foxe1 subsequently becomes required to sustain chromatin accessibility at the Pax8 locus (supported by Figure 6B and predicted biding site, Foxe1 motif, Table S1).
3. In Foxe1 KO cells, accessibility at the Pax8 locus collapses, reducing Pax8 expression and weakening thyroid super-enhancer activity.
4. As the thyroid transcriptional network destabilizes, Nkx2-1, still expressed, can cooperate with lung-associated cofactors at already accessible lung loci.
5. Lung transcription increases without requiring *de novo* chromatin opening, consistent with redistribution of limiting transcriptional machinery.

Supporting a more direct regulatory role, motif analysis revealed a predicted Foxe1 binding site within regulatory regions of the Pax8 locus (Table S1). This is consistent with the possibility that Foxe1 directly binds Pax8-associated enhancers, potentially recruiting chromatin remodelers and/or stabilizing enhancer-promoter interactions required to maintain high Pax8 expression. While functional validation of this binding will require future studies, this observation further supports a model in which Foxe1 actively maintains Pax8 chromatin accessibility rather than indirectly affecting thyroid identity.

Interestingly, our newly added data (Figure S8A-C) show that complete absence of Pax8 (Pax8KO mESCs) does not result in the same phenotype, displaying a complete absence of thyroid or lung organoids. This finding reinforces the hypothesis that Foxe1 is not regulating Pax8 expression at early stages of thyroid specification.

Furthermore, our previous single-cell RNA-seq analysis of mouse thyroid organoids (Romitti et al., *Frontiers in Endocrinology*, 2021) did not reveal substantial lung cell population under wild-type conditions, with only a small Nkx2.1-Krt5 cluster, called non-thyroid epithelial cells being identified. This suggests that high Pax8 levels in the presence of Foxe1 effectively commit most Nkx2.1+ progenitors toward thyroid fate.

Despite this, we agree that expansion of rare lung-competent cells, even if unlikely, cannot be formally excluded. Definitive resolution of whether a bipotent Nkx2.1+ progenitor with dual thyroid and lung potential exists would require dedicated lineage tracing at single-cell resolution. Such experiments would be necessary to distinguish between fate conversion and expansion of lineage-competent progenitors and lie beyond the scope of the current study.

Ultimately, we have extensively revised the manuscript to clarify these points and to avoid implying direct fate switching. Our data instead support a model in which Foxe1 stabilizes thyroid commitment by maintaining Pax8 enhancer accessibility, thereby functionally restricting Nkx2.1 from engaging alternative foregut programs.

All the above-mentioned information and discussion have been incorporated to the new version of the manuscript

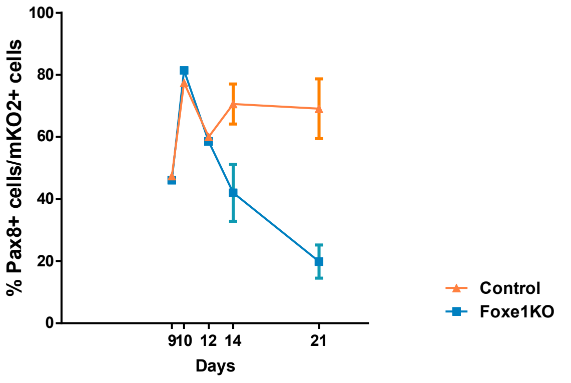


**Percentage of Pax8+ cells among Nkx2-1-mKO2 cells at different time points of control/Foxe1KO mESCs culture.** Immunostaining followed by flow cytometry quantification. Mean and error (SEM).

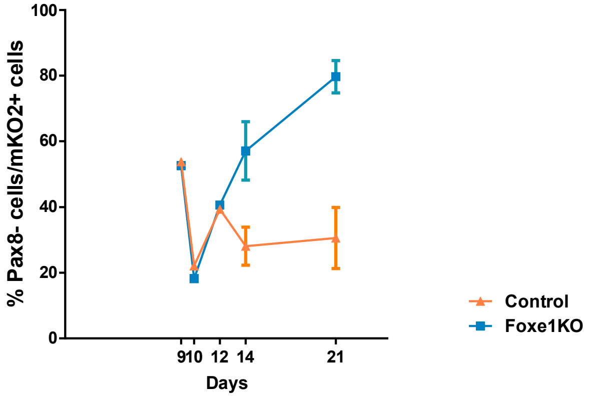


**Percentage of Pax8- cells among Nkx2-1-mKO2 cells at different time points of control/Foxe1KO mESCs culture.** Immunostaining followed by flow cytometry quantification. Mean and error (SEM).

*Observations that Foxe1KO did not at all influence gene expression in expanding lung-like cells are consistent with the idea that lung and thyroid specification in the model are independent phenomena, and argue against the existence of a common bipotent progenitor. If authors disagree, this issue and question should be more thoroughly discussed and argued for with more supporting experimental data than found in the current manuscript version*

We thank the reviewer for this important comment. As stated above, we agree that our current data do not formally demonstrate the existence of a common bipotent progenitor, and we have revised the manuscript to avoid overinterpretation in this regard.
Regarding lung genes expression, we observe significant differences between WT and Foxe1 KO organoids at day 22, as assessed by qPCR (see Figure S3). In addition, single-cell RNA sequencing reveals the presence of distinct lung cell populations in the Foxe1 KO condition, characterized by high expression of specific lung lineage markers (see Figure 4). Importantly, these lung populations were not detected in our previous single-cell RNA-seq analysis of WT thyroid organoids (Romitti et al., *Frontiers in Endocrinology*, 2021), except for a small population of Nkx2-1+Krt5+ cluster, indicating that their emergence is specifically associated with Foxe1 loss.
Despite the appearance of these lung-like cell types in Foxe1 KO organoids, ATAC-seq analysis does not reveal increased chromatin accessibility at canonical lung regulatory loci compared to

WT (see Figure 6). This suggests that Foxe1 does not act as a direct negative regulator of the lung program. Rather, our data support a model in which Foxe1 primarily maintains thyroid lineage stability by sustaining chromatin accessibility at the Pax8 locus. In its absence, Pax8 expression is reduced, thyroid enhancer activity collapses, and thyroid differentiation is compromised.

Consequently, Nkx2-1+ cells remain in a transcriptionally permissive state in which lung-associated loci, already epigenetically accessible in foregut-derived progenitors, can be engaged. Thus, lung differentiation appears to arise not through active induction by Foxe1 loss, but through destabilization of the thyroid program, allowing Nkx2-1 to cooperate with alternative cofactors within an already permissive chromatin landscape.

To prevent misunderstanding, we have modified the title and substantially clarified the results and discussion sections to better reflect this model and to avoid implying direct lineage instruction or proven bipotency.

*Minor comments*

*What is the fraction of. Nkx2-1+ cells that organize into follicles vs lung structures? Based on provided overview images (e.g. Figs. S1, S4) the general impression is that most cells do not form 3D-structures (i.e. do not differentiate). Please explain this and provide information in paper.*

We thank the reviewer for this helpful comment and for the opportunity to clarify this point. First, the images shown in Figs. S1B–C correspond to day 7 and Fig. S4E to day 10 of the differentiation protocol. As indicated in the figure legends, these represent early stages of the culture during which cells are still a pool of progenitor-like cells. At these time points, organized 3D thyroid follicles or lung-like epithelial structures are not yet formed. We have revised the figure legends to ensure this is clearly stated and to avoid the impression that full differentiation has already occurred at these stages.

Regarding the fraction of Nkx2-1$^+$ cells that organize into follicles *vs.* lung structures at later stages, we acknowledge that we are not able to provide an exact quantitative proportion. Due to the 3D nature of the culture system and the size heterogeneity of the structures, precise counting of all Nkx2-1$^+$ cells within organoids are technically challenging. However, based on representative images (e.g., Fig. 1C) and repeated observations across independent experiments, a subset of Nkx2-1$^+$ cells clearly organize into epithelial 3D structures, while others remain unorganized or in less structured aggregates.

In the Foxe1 KO condition, the larger size and morphology of the epithelial structures suggest that a substantial proportion of Nkx2-1$^+$ cells contribute to lung-like structures. Morphologically, these structures are typically larger (approximately 70–600 μm) compared to thyroid follicles (approximately 30–50 μm), supporting the impression that lung-like structures represent a significant fraction of organized epithelia in the KO condition.

Importantly, our single-cell RNA-seq data provide additional support for epithelial organization within defined clusters. The Nkx2-1/lung clusters express high levels of epithelial markers such as Epcam and Cdh1 (E-cadherin), consistent with structured epithelial identity. In contrast, only the Thyroid 1 cluster expresses these epithelial markers robustly, whereas the Thyroid 2 and

#

Nkx2-1⁺/Pax8⁻ clusters show low or absent expression, suggesting that not all Nkx2-1⁺ cells acquire a fully organized epithelial state.

*Fig. 1C: Supposed follicles are not shown in this graph.*

We thank the reviewer for pointing this out. We agree that, due to the low magnification, individual follicular structures are not clearly discernible in Fig. 1C. The purpose of these images was not to illustrate fully formed thyroid follicles, but rather to highlight the relative proportion of Nkx2-1⁺/Pax8⁺ double-positive cells in control versus Foxe1 KO conditions.
To avoid confusion, we have revised the figure legend and the text and replaced the term “thyroid follicles” with “thyrocytes,” which more accurately reflects what is shown at this magnification. We believe this clarification better aligns the description with the intent of the figure.

*Why does not thyroglobulin accumulate in lumen (which if present would be a good means for quantification by counting follicles)?*

We thank the reviewer for this valuable suggestion and agree that luminal thyroglobulin (Tg) accumulation would, in principle, represent an informative readout for follicle quantification. However, our organoids display a fetal-like developmental state and exhibit heterogeneity in maturation and functional competence (as expected *in vivo* at early development). As we have previously demonstrated (Carvalho et al., *Advanced Healthcare Materials*, 2023), even in highly mature thyroid organoid systems, not all morphologically defined follicles are functionally active. Thus, the absence or variability of luminal Tg or iodinated Tg (Tg-I) accumulation does not necessarily indicate absence of follicle formation at this developmental stage. In other words, Tg accumulation is not a fully reliable surrogate marker for follicle presence in this context. Here we included an example of Tg staining in mouse thyroid organoids, where we can observe some regions with Tg accumulated in the lumen, while most of the cells also show (or exclusively) cytoplasmatic staining. This image further confirms the variability in Tg accumulation among derived organoids.
To more accurately identify follicular structures, we relied on epithelial polarity and architectural markers. Specifically, we used E-cadherin and ZO-1 staining in combination with Pax8 to define organized epithelial thyroid structures. In addition, we employed an iodinated-thyroglobulin antibody (mouse anti–Tg-I, gift from C. Ris-Stalpers) and improved the quality of the Tg-I staining in Fig. 1E. This was further complemented by the Tg-EGFP reporter signal to better visualize thyroid follicular organization.
Nevertheless, due to the intrinsic 3D nature of the culture system and structural heterogeneity of the organoids, precise quantitative assessment remains technically challenging.

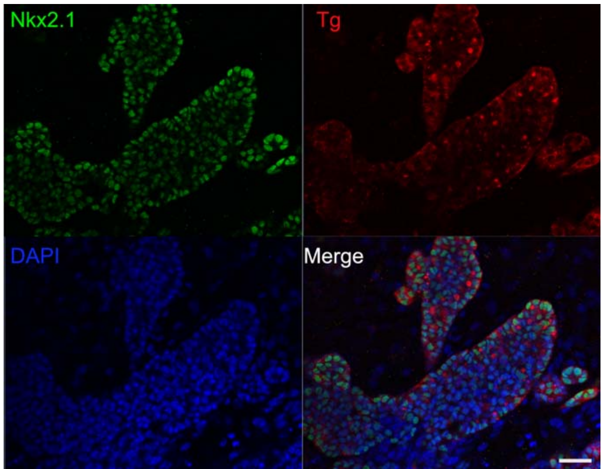


**Immunostaining of WT control-derived organoids (D21) showing thyroid structures (Nkx2.1) with Tg localization heterogeneity. Signal is observed both in the cytoplasm and in the lumen.** Scale bar 100um.

*Indeed, follicles should be quantified to estimate induction success. Please also explain rounded structures in Foxe1KO image (are they distal lung buds?). Or are Control and Foxe1KO images confused in this panel?!?*

We thank the reviewer for this important comment and for raising the need for quantitative assessment.

To estimate induction efficiency and directly compare control and Foxe1 KO conditions, we quantified Nkx2-1$^+$ and Nkx2-1$^+$/Pax8$^+$ populations by flow cytometry (Fig. S6A-B), using the Nkx2-1_mKO2 reporter in combination with Pax8 antibody staining. We observed a marked reduction in the total number of Nkx2-1$^+$ cells in Foxe1 KO organoids compared to controls, beginning at day 11 and becoming progressively more pronounced over time. By day 21, approximately 40-50% of cells in the control condition are Nkx2-1$^+$, whereas only ~10-15% are Nkx2-1$^+$ in the Foxe1 KO.

Importantly, co-staining with Pax8 further revealed that in control organoids, the majority of Nkx2-1$^+$ cells are also Pax8$^+$ (41.9% of total cells), consistent with efficient thyroid commitment. In contrast, in Foxe1 KO organoids, only 3.1% of total cells are double positive, indicating a profound reduction in thyroid lineage. These quantitative data provide a robust measure of induction success and lineage specification efficiency.

Regarding the rounded structures shown in Fig. 1D in the Foxe1 KO condition, the images are correctly assigned and not confused. These rounded epithelial structures represent the few thyroid follicles that form in the absence of Foxe1, as confirmed by Pax8 and Tg positivity. Although markedly reduced in frequency, follicle formation is not completely abolished in the KO condition. However, as highlighted in Fig. 1D, these self-organized follicles are not functionally mature, as evidenced by the absence of Nis/Slc5a5 expression. An additional example of a follicle derived in the Foxe1 KO condition is shown in Fig. S5B.

*Fig. 1E: text on Fig. legend is erroneously given under (F), whereas a dedicated and relevant text for (F) is missing.*

We thank the reviewer for this careful observation. The figure legend has been corrected to properly assign the text to panel (E), and a dedicated legend describing panel (F) has now been added. In addition, we have ensured that the corresponding figure panels are appropriately referenced in the main text.

*Fig. 1F. Immunostaining of iodinated thyroglobulin (Tg-I) is very poor. Is it due to a bad antibody (does it work well in in vivo thyroid stainings?) or is organification simply inefficient? Again, poor content of Tg in lumen (as also suggested by Fig. S5A), it is puzzling. Or are in vitro-generated follicles leaky (i.e. do not behave as natural thyroid follicles)?*

We thank the reviewer for this helpful comment. Following this suggestion, we have improved the quality of the iodinated thyroglobulin (Tg-I) immunostaining and included new images at higher quality and different magnifications in Fig. 1E. These revised images more clearly show the accumulation of Tg-I within the luminal compartment, particularly in the WT control condition.

Regarding the apparent variability in Tg accumulation, we believe this reflects the fetal-like developmental state of the organoids and the heterogeneity in their maturation and functional competence. As discussed above, not all follicles generated in vitro reach the same level of functional maturity, which may influence the degree of Tg accumulation within the lumen.

Importantly, we do not believe that the in vitro–derived follicles are structurally leaky. First, the luminal localization of iodinated Tg is clearly detectable in Fig. 1E, indicating that Tg can accumulate within the follicular lumen. Second, functional assays presented in Fig. 1F demonstrate robust iodide uptake and organification, supporting the presence of an active thyroid hormone biosynthetic machinery in these organoids.

*Figs. 2A-E: Comments on lung cell markers. A: E-cad is unspecific, Sox9 would better label branching morphogenesis*

We thank the reviewer for this helpful comment. The purpose of the first panel in Fig. 2 (A) was to highlight the presence of Nkx2-1$^{+}$ cells organizing into epithelial structures, as indicated by E-cadherin staining. In this context, E-cadherin was used to visualize epithelial organization rather than to specifically identify lung lineage cells. This also allowed us to emphasize the clear morphological differences between thyroid follicles, which are typically smaller, and the larger epithelial structures observed in the Foxe1 KO condition that are consistent with lung-like structures.

The presence and identity of specific lung cell populations are further addressed in the subsequent panels of Fig. 2 (B-H) and more comprehensively in the single-cell RNA-seq dataset presented in Fig. 4.

While we agree that Sox9 staining would provide an additional marker for bud tip progenitors and branching morphogenesis, our single-cell RNA-seq analysis shows Sox9 expression within the Nkx2-1$^{+}$/Epcam$^{+}$/Pax8$^{-}$/Tg$^{-}$ population in Foxe1 KO organoids (Fig. 4B), supporting the presence of this lung progenitor population in our system.

Finally, it is important to note that our culture system (media) is not designed to promote lung development in vitro, which probably impairs the proper physiological lung tissue formation and differentiation progress observed in optimal systems and in vivo. In addition, we believe that we have fetal-like lung organoids in vitro, as comparison to scRNAseq of E17.5 suggests. These aspects were also discussed in the new version of the manuscript.

*C: co-staining for E-cad would help differentiate cell types. D: Goblet cells seem Nkx2-1 negative, please explain.*

We thank the reviewer for these helpful comments.

Regarding the suggestion to include E-cadherin co-staining to better distinguish cell types, we agree that this would provide additional spatial information. However, due to technical limitations related to the species of the primary antibodies used for several lung lineage markers, we were unable to include E-cadherin co-staining in many of the panels. To address epithelial identity at the transcriptomic level, in our single-cell RNA-seq analysis we specifically filtered for $Nkx2\text{-}1^{+}$ cells that were also $Epcam^{+}$, thereby focusing the analysis on epithelial populations present in the organoids (Fig. 4A). Consistent with this approach, the lung-related clusters identified in the dataset (Fig. 4B) show clear expression of epithelial markers, including Epcam and Cdh1 (E-cadherin) (Fig. 3E), supporting their epithelial nature.

Regarding the observation that goblet cells appear Nkx2-1 negative, we note that the Muc5ac staining shown in Fig. 2D primarily reflects secreted mucin that accumulates within the lumen of the lung-like epithelial structures rather than intracellular staining confined to individual goblet cells. As a result, the signal is predominantly detected in the luminal space, which may give the impression that it is not associated with Nkx2.1-expressing cells. To clarify this point, we provide images highlighting Muc5ac accumulation within epithelial structures that express Nkx2.1 (Fig. 2D) and Sox2 (Fig. 2F). In addition, Fig. S5C shows a large $Nkx2\text{-}1\_mKO2^{+}/Sox2^{+}$ epithelial structure with clear Muc5ac accumulation in the lumen, supporting the presence of goblet-like secretory activity within these Nkx2.1–derived lung structures.

*E: Diffuse pattern. Are assumed club cells really Nkx2-1 pos? CC10 immunostaining might help.*

We thank the reviewer for this helpful comment. The diffuse pattern observed in Fig. 2E is largely due to the 3D reconstruction of the image, which can reduce the apparent sharpness of individual cellular boundaries. Nevertheless, the image indicates that Scgb3a2+ cells are located within epithelial structures containing Nkx2.1–expressing cells.
Following the reviewer's suggestion, we have now included additional immunostaining for Cc10/Scgb1a1 in the revised manuscript (Fig. 2G), which further supports the presence of club-like cells in the organoids. Although we were unable to show direct co-staining with Nkx2-1, our single-cell RNA-seq analysis confirms that all $Scgb3a2^{+}$ and $Scgb1a1/Cc10^{+}$ cells identified in the organoids belong to a $Nkx2\text{-}1^{+}/Epcam^{+}$ epithelial population (Fig. 4A–B and Fig. S7A). This is further illustrated in the corresponding UMAP plots shown below.
Together, these data support the interpretation that the $Scgb3a2^{+}$ and $Cc10^{+}$ cells detected in the organoids correspond to Nkx2.1-derived epithelial club-like cells.

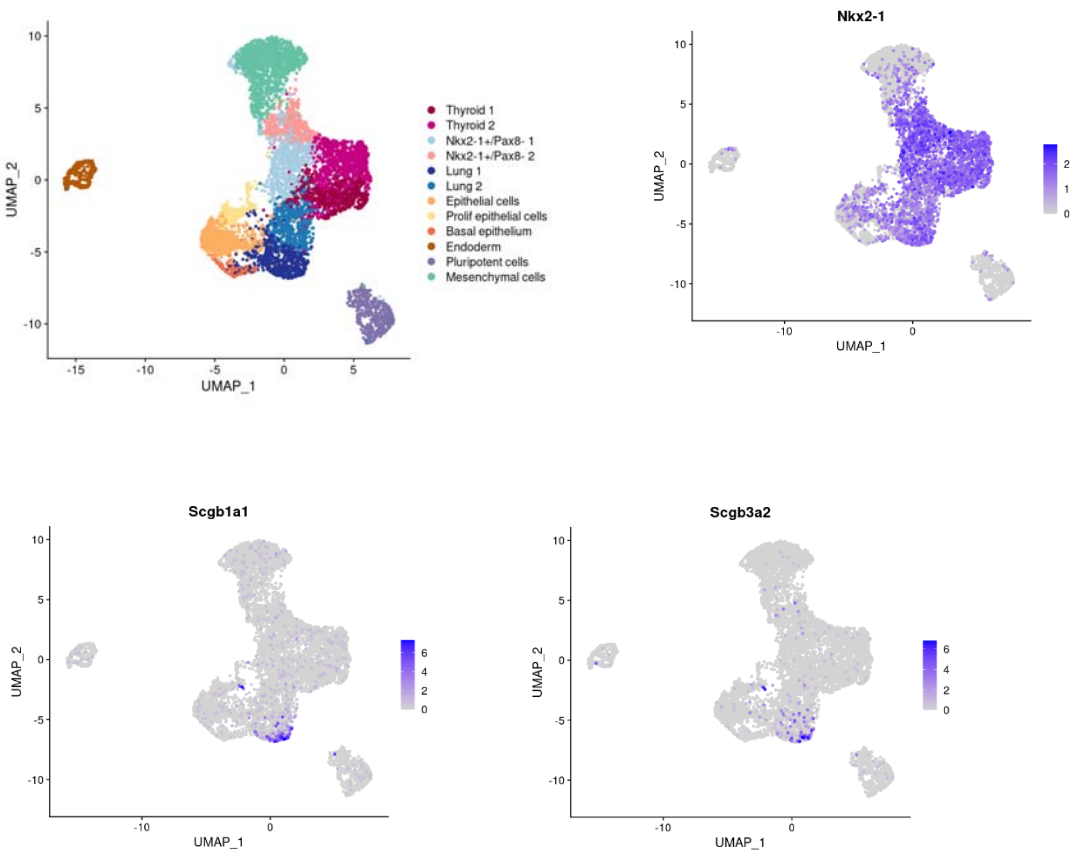


*F: I doubt that SEM is conclusive for identification of specific (lung) cell types unless tissue architecture (e.g. proximal-distal positions) is considered for comparison to the natural branching process of the developing lung.*

We agree with the reviewer that SEM alone is not sufficient for the definitive identification of specific lung cell types. In this study, SEM was used to visualize ultrastructural features and morphological characteristics suggestive of differentiated epithelial cell types, based on comparisons with SEM images from human/mouse lungs. Importantly, our organoids do not represent adult lung tissue, but most likely fetal stages of lung development, this is an important aspect since cells might not display full features of adult lungs; e.g. ciliated cells show rather short cilia, compatible with early development. Similar aspect is observed with alveolar structures, that are most likely developing-alveolar sacs. This important aspect of developmental stage is now described in the figure legend (Fig. 2H).

To improve the clarity of our SEM images, we modified the figure and replaced images that had not very clear features by new ones. We included a new image showing mucus accumulation in the luminal compartment, a larger view of developing-alveolar sacs and alveolar cells, with a zoomed image of AT2 cell. In addition, epithelium containing secretory cells and mucus blobs was included.

Importantly, cell identity in our study was not inferred from SEM alone. We used several complementary approaches, including immunostaining, qPCR analysis, and single-cell RNA

sequencing, to support the identification of the different lung epithelial populations present in the organoids.
Nevertheless, we have decided to retain instead improving the SEM images in Fig. 2H, as they provide valuable ultrastructural characterization of the organoids and illustrate morphological features consistent with differentiated lung epithelial cells.

*Line 161: Is it really "spontaneous" generation? Please rephrase.*
We thank the reviewer for this suggestion. The term “spontaneous” has been replaced with “unexpected” to more accurately describe the generation of these structures.

*Fig. S3A. According to Major Comment above, please explain in more detail why and how lung marker expression is evident in induced "Controls" (i.e. organoids without Foxe1KO). Is it due to parallel/independent lung and thyroid differentiation? Is phenotype of rather Foxa1KO a matter of clonal selection?*

Back to our previous response, the low lung marker expression observed in control organoids likely reflects the presence of Nkx2-1$^{+}$ foregut progenitors that remain transcriptionally permissive to alternative Nkx2.1–dependent programs. In wild-type conditions, the majority of Nkx2.1$^{+}$ cells co-express Pax8 (~80%), indicating robust thyroid commitment, still with around 20% of the cells not committing to thyroid, what could explain an “inefficient” parallel lung differentiation in presence of Foxe1. In contrast, in Foxe1 KO organoids this proportion drops to ~20%, reflecting destabilization of the thyroid transcriptional network rather than induction of a new lineage. Consistent with this, chromatin accessibility analyses show reduced accessibility at the Pax8 locus in Foxe1 KO cells without significant gain at canonical lung loci. Together, this process could allow the expansion of the non-thyroid committed progenitors and acquisition of lung cell fate due to the permissive state of the chromatin. While expansion of rare lung-competent progenitors cannot be formally excluded, distinguishing between lineage plasticity and clonal expansion would require dedicated lineage-tracing experiments beyond the scope of this study.

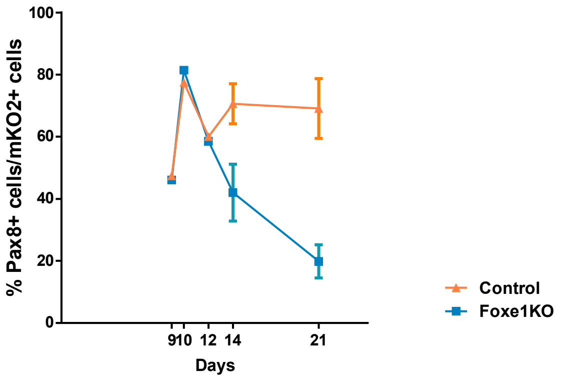


**Percentage of Pax8+ cells among Nkx2-1-mKO2 cells at different time points of control/Foxe1KO mESCs culture.** Mean and error (SEM).

*Figs. S3B-M. Scanning electron micrographs. Are these from one single (lung-like) structure imaged at different angles and magnitude or selected from multiple/different structures? If the*

*latter, there a bias of selection that raises concern about cell identity. See similar SEM comment above.*

We thank the reviewer for this important point. The SEM images in the old Figures S3B–M did represent distinct lung-like structures rather than multiple angles of a single organoid, as we could not obtain representative images of all cell types from the same structure. However, the SEM data presented in Figure 2 already sufficiently highlight the distinct cell types and structures. To avoid redundancy, we have therefore removed panels S3B-M in the revised version of the figure.

*Line 181: Text states that cells additionally were visualized by microscopy, but this is not shown in Fig. 4.*

We thank the reviewer for pointing this out. The sentence has been revised to clarify that the reporter fluorescence can be used to track differentiation by microscopy, while the efficiency of Nkx2.1$^{+}$ cell generation is quantified by flow cytometry, as shown in Figure S4D–E rather than Figure 4. The updated sentence reads:

*"The reporter fluorescence allowed tracking the Nkx2-1+ cells appearance by microscopy and quantification of the differentiation efficiency by flow cytometry (Figure S4D-E)."*

*Fig. 4. Data based/biased on computationally Pax8-negative selected Foxe1KO cells. Are Pax8 negative cells present in "Control" (Foxe1+) organoids and a potential source of enrichment independent of the thyroid lineage?*

We thank the reviewer for raising this important point, which prompted us to further examine the Nkx2.1$^{+}$/Pax8$^{-}$ cell populations in both control and Foxe1 KO samples. Flow cytometry analysis (shown below) indicates that the proportion of Pax8$^{+}$ and Pax8$^{-}$ cells among mKO2$^{+}$ (Nkx2-1$^{+}$) cells was comparable between control and Foxe1 KO organoids at day 9, two days after completion of doxycycline induction. This suggests that both thyroid and lung lineages were initially induced at similar levels in the two cell lines.

This trend persists until day 12, when a clearer divergence between thyroid and lung fates begins to emerge in control versus Foxe1 KO organoids. Overall, these results indicate that Foxe1 expression reinforces thyroid lineage specification, whereas Foxe1 knockout results in an expansion of Nkx2.1$^{+}$/Pax8$^{-}$ cells. Importantly, the PCA analysis of ATAC-seq data presented in Fig. 5G supports this conclusion.

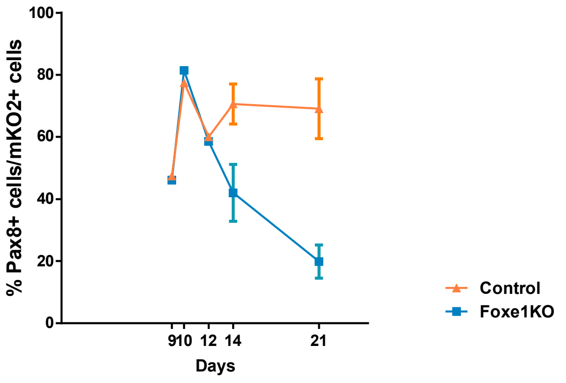


**Percentage of Pax8 positive cells among Nkx2-1-mKO2 cells at different time points of control/Foxe1KO mESCs culture.** Mean and error (SEM).

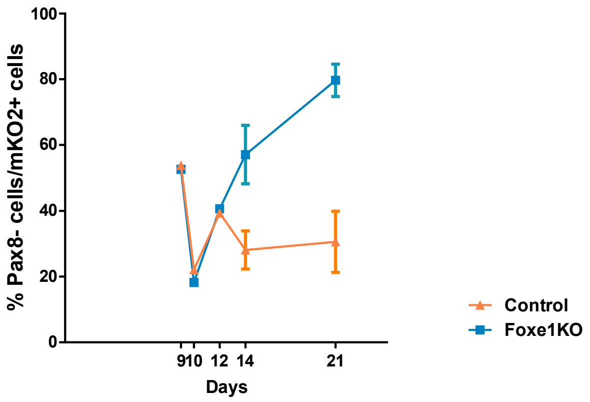


**Percentage of Pax8 negative cells among Nkx2-1-mKO2 cells at different time points of control/Foxe1KO mESCs culture.** Mean and error (SEM).

*The paper by Fagman et al. (Am J. Pathol, 2004), which shows aberrant/ectopic thyroid differentiation in airway respiratory epithelium in ShhKO mouse embryos, may by cited and discussed with reference to the possible existence of bipotent lung/thyroid progenitors/stem-like cells in vivo.*

We thank the reviewer for this valuable suggestion and apologize for not citing this highly relevant study in the previous version of the manuscript. We have now incorporated a discussion of this work in the final paragraph of the revised manuscript.

Added text in the manuscript:
*"In conclusion, the present work advances our understanding of the critical role of Foxe1 in initiating and sustaining proper thyroid tissue formation and function, while also highlighting novel molecular players for future investigation in thyroid biology. Beyond the thyroid, our findings underscore the intricate relationships among endodermal lineages during differentiation, particularly between thyroid and lung. Supporting this concept, in vivo studies by Fagman and collaborators (2004) showed that loss of Shh signaling during early organogenesis leads to thyroid dysgenesis and the appearance of aberrant thyrocytes expressing Nkx2-1, Foxe1, and Tg in the presumptive trachea, emphasizing the need to repress inappropriate thyroid programs in non-thyroid anterior foregut endoderm (Fagman et al., 2004). Building on*

*this, it is intriguing to speculate that transient thyroid/lung bipotent progenitors may exist in vivo, analogous to the transient bipotent progenitors described during liver and pancreas development (Deutsch et al., 2001; Xu et al., 2011). Future studies using lineage tracing approaches could directly test the existence and fate of such progenitors, providing a deeper understanding of early endodermal plasticity and the mechanisms that safeguard lineage fidelity."*

***Reviewer #1 (Significance (Required)):***

*The results are indeed of great value mainly for developmental biologist interested in regenerative medicine and specifically concerning in vitro systems for lung and thyroid differentiation. The provided single cell data sets of thyroid progenitors undergoing differentiation and the impact of Foxe1KO are a major achievement and resource.*

*This reviewer´s expertise is mainly in vivo thyroid development.*

***Reviewer #2 (Evidence, reproducibility and clarity (Required)):***

*Summary: This study by Fonseca et al investigated how the specification of mouse ESCs towards thyroid lineage was regulated by the presence or absence of Foxe1, a thyroid specific transcriptional factor. Compromised thyroid induction was observed when Foxe1 was knocked out. Interestingly, the author found increased induction of lung cells in the absence of Foxe1, suggesting its role in regulating the balance of thyroid-versus-lung specification. While interesting, the main issue with this study is the lack of quantitative analysis of cellular specification, and the lack of comprehensiveness regarding the markers used to characterize each cell lineage, especially for the lung lineages.*

*Major points:*

*For analyzing the outcome of lineage specification in the comparison of with and without Dox or in the comparison of control versus Foxe1 KO, the only quantitative readout is qPCR. The author should perform additional characterization using flow cytometry for NKX2.1, Pax8, Tg, Tg-I, Ecad, and ZO-1 to reveal more clear mechanism: reduced number/percentage of cellular specification into thyroid lineage, or immature phenotypes in specified thyroid cells.*

We thank the reviewer for raising this important point. We agree that incorporating quantitative analyses is essential to confirm the phenotype driven by the loss of Foxe1 expression. To address this comment, we have added additional flow cytometry analyses at different time points throughout the culture in the revised manuscript (Fig. S6A–B). Specifically, we now include quantification of Nkx2.1/mKO2$^{+}$ cells and Tg/GFP reporter$^{+}$ cells in both control and Foxe1KO organoids from day 7 to day 21 of the differentiation protocol.

These data show that up to day 10 there is no significant difference in the proportion of Nkx2.1$^+$ cells generated under the two conditions. However, from day 11 onwards the trajectories diverge clearly: in control organoids, Nkx2.1$^+$ cells reach approximately 50% of the population, whereas only 10–15% of cells become Nkx2.1$^+$ in the Foxe1KO condition (Fig. 6A and Fig. S6B). These findings are consistent with the reduced proportion of Nkx2.1$^+$Pax8$^+$ cells observed in Foxe1KO organoids (Fig. 6B and Fig. S6B), confirming the impairment in thyroid cell generation caused by the loss of Foxe1 expression. In addition, although not the most precise measure, we also observed a similar reduction in the proportion of Tg-GFP$^+$ cells in the Foxe1KO condition compared with controls (Fig. 6A).
While these new results provide additional quantitative insight, accurately assessing the maturation state of the generated thyroid cells by flow cytometry remains challenging due to several technical limitations:

1. **Tg quantification:** Despite testing several anti-thyroglobulin antibodies for flow cytometry, we were unable to obtain reliable staining. For this reason, we included quantification of the Tg/GFP reporter described above. Despite the clear reduction in Tg+ cells among Foxe1 KO organoids, we previously demonstrated (Romitti and Eski et al., 2021; Fig. 2E) that the GFP reporter captures only a fraction of the Tg$^+$ cell population present in the culture, not being the most accurate method for quantification.
2. **Tg-I and ZO-1 quantification:** Due to their intraluminal and apical localization within thyroid follicles, quantification of Tg-I is not possible by FC and ZO-1 staining has demonstrated to be technically difficult and did not yield reliable results.
3. **Assessment of immature vs. mature thyrocytes:** We believe that the combined datasets presented in Fig. 1 and the scRNA-seq analysis (Fig. 3) provide sufficient evidence to interpret the Foxe1KO phenotype. Together, these results indicate that: (i) Foxe1KO organoids show a reduced efficiency in generating thyrocytes and Nkx2.1+ cells compared with the control line; and (ii) the few thyrocytes that form in the absence of Foxe1 display impaired maturation.

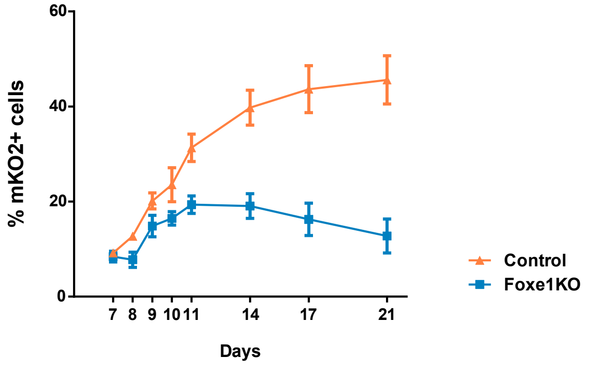


**Figure 4: Percentage of Nkx2-1-mKO2 cells at different time points of control/Foxe1KO mESCs culture.** n= 5; mean and error (SEM).

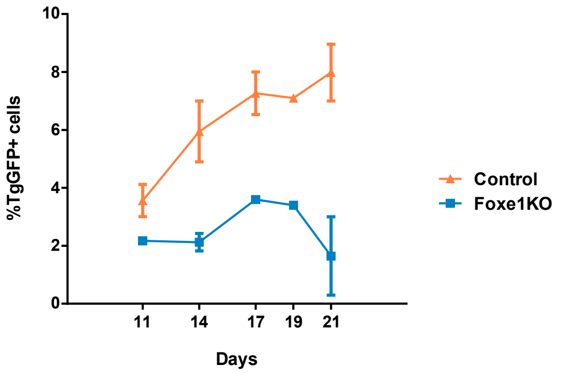


**Figure 5: Percentage of TgGFP positive cells at different time points of control/Foxe1KO mESCs culture.** N=5. Mean and error (SEM).

*The authors claimed that in the absence of Foxe1, lung organoid can be observed. Quantitative analysis, such as organoid count or flow cytometry, should be provided to assess this comparing organoid identities in the presence and absence of Foxe1.*

We thank the reviewer for this important comment and we agree that a precise quantification would reinforce our findings on organoid identities. As described above, we performed flow cytometry analyses to track Nkx2.1/Pax8 cell populations over time in both WT and Foxe1KO conditions. In the WT condition, approximately 80% of Nkx2.1$^{+}$ cells are also Pax8$^{+}$, consistent with thyroid lineage specification. In contrast, in the Foxe1KO condition, only ~20% of Nkx2.1$^{+}$ cells co-express Pax8, indicating a strong reduction in thyroid lineage commitment.
Although this approach does not directly quantify lung organoids, our scRNA-seq data show that the majority of Nkx2.1$^{+}$Pax8$^{-}$ cells in the Foxe1KO condition display an epithelial transcriptional profile, with a substantial proportion exhibiting a lung-like signature.
Regarding a direct quantification of the proportions of each organoid type, we encountered several technical limitations inherent to organoid systems. In particular, variability between wells and differentiations, combined with the three-dimensional complexity of the cultures, makes reliable counting of distinct organoid identities challenging.
With respect to flow cytometry-based quantification of lung identity, the diversity of lung epithelial cell types represents an additional challenge. Available markers often label only specific subpopulations and can overlap with thyroid markers. For example, Sox2 labels airway epithelial cells but not alveolar cells, whereas Sox9, which can mark distal lung progenitors, is also highly expressed in thyrocytes. Similarly, assays with secretory cell markers (Scgb3a2, Scgb1a1, and Muc5ac) did not yield reliable staining in our system. Hopx, an alveolar marker, is also detected in the thyroid population. Although thyroid cells can be specifically identified by Pax8 staining, this overlap further complicates the combination of markers required for reliable flow cytometry quantification of lung lineages.
Taken together, and considering that in our previous work we demonstrated by scRNA-seq that lung differentiation is not clearly observed in the control line, with only a small subset of Nkx2-1+Krt5+ cluster been detected (Romitti and Eski et al., 2021), our quantitative analyses rely primarily on Nkx2.1/Pax8 flow cytometry together with the transcriptional evidence provided by scRNA-seq.

*In Figure 2, the claim of lung cell identities is not well supported. (1) SEM data on alveolar and goblet cells is not conclusive;*

We agree with the reviewer that SEM alone is not sufficient for the definitive identification of specific lung cell types. In this study, SEM was primarily used to visualize ultrastructural features and morphological characteristics suggestive of differentiated epithelial cell types, based on comparisons with previously reported SEM images of human and mouse lung tissue. Importantly, our organoids do not represent adult lung tissue but rather likely correspond to early developmental stages of lung formation. This is an important consideration, as cells at these stages may not display all the morphological hallmarks observed in mature lungs. For example, the ciliated cells observed in our organoids present relatively short cilia, which is consistent with early stages of airway epithelial development. Similarly, the structures resembling alveoli are more consistent with developing alveolar sacs rather than fully mature alveoli. This developmental context is now clarified in the figure legend (Fig. 2H).

To improve the clarity and interpretability of the SEM data, we revised the figure and replaced images in which the features were not sufficiently clear. The updated panel now includes images showing mucus accumulation within the luminal compartment, a broader view of developing alveolar sac–like structures, and a higher-magnification image highlighting cells with morphology consistent with alveolar type II–like cells. In addition, we included images of epithelial regions containing secretory cells and mucus deposits.

Importantly, cell identity in our study was not inferred from SEM alone. Instead, we used several complementary approaches, including immunostaining, qPCR analyses, and single-cell RNA sequencing, to support the identification of the different lung epithelial populations present in the organoids.

For these reasons, we chose to retain the SEM data in Fig. 2H while improving the image selection and annotations, as these images provide valuable ultrastructural information and illustrate morphological features consistent with differentiated lung epithelial structures.

In addition, it's important to note that our system is not designed (culture media composition) for optimal generation of lung organoids and we believe that despite of the indications of fetal-like lung organoids generated they might not follow the expected physiological path observed in vitro optimal models and in vivo. It could impact the maturity and the proportions of the cells derived. This discussion is also now present in the updated version of the manuscript.

*(2) Alveolar type 1 cells should be characterized by AGER and AQP5 besides HOPX*

We thank the reviewer for this valuable suggestion and agree that additional markers such as AGER and AQP5 would further support the identification of alveolar type I (AT1) cells. Following the reviewer's recommendation, we performed additional immunostainings using AQP5 and AGER antibodies. However, we were unfortunately unable to obtain reliable staining that would clearly demonstrate AT1 cells in our organoid system.

Nevertheless, both AQP5 and AGER transcripts are detected in the lung-like populations in our scRNA-seq dataset (Fig. 4 and examples shown below). Interestingly, their expression is not restricted to a single well-defined cluster, which may reflect the transitioning/immature state of the lung-like cells present in the organoids. Additional comparison to *in vivo* dataset suggests an

#
enrichment in AT1 signature in cluster 0, which contains Foxe1KO-derived cells, however it might not reflect fully maturation of this cell type.
Taken together, these observations further reinforce that while lung epithelial populations are present, the organoids likely represent an early developmental stage of lung differentiation rather than fully mature lung tissue, and therefore may not yet exhibit the clear marker segregation characteristic of adult alveolar cell types.

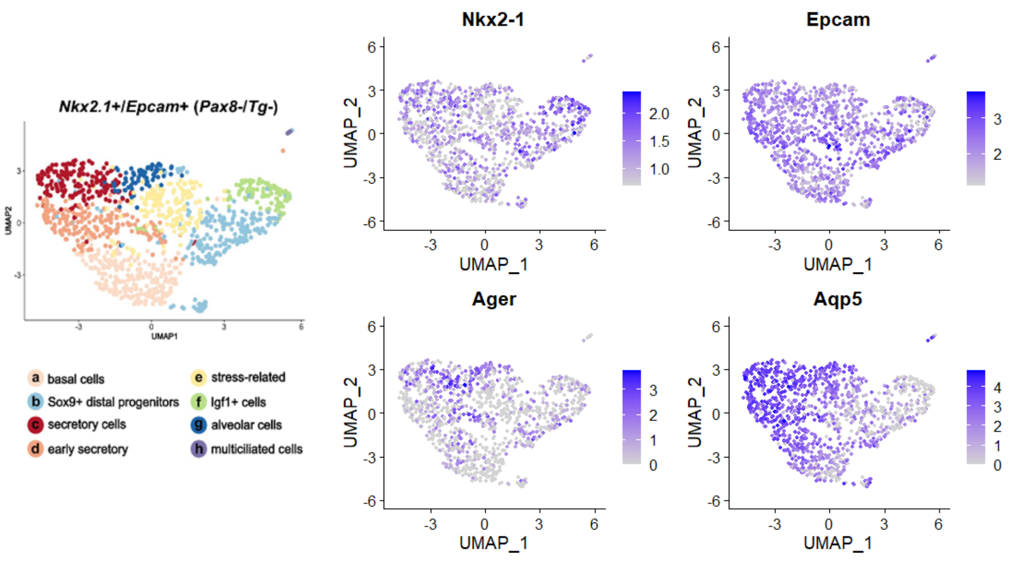


**UMAP expression plots showing Ager and Aqp5 lung markers in the Nkx2-1+/Epcam+(Pax8-/Tg-) cell population.**

*(3)*

*Alveolar types 2 cells should be characterized by NKX2.1 and SFTPC co-staining;*
Dear reviewer, as mentioned in the previous comment, we encountered similar technical difficulties when attempting SFTPC immunostaining, and we were unfortunately unable to obtain reliable staining in our organoid system.
In contrast to Aqp5 and Ager, Sftpc transcripts were not detected in our scRNA-seq dataset. However, several other markers commonly associated with AT2 cells, such as Napsa, Abca3, and Lpcat1, are expressed in the lung-like populations (examples shown below). In addition, comparative analyses with an *in vivo* mouse lung dataset indicate transcriptomic similarities between E17.5 AT2 cells *in vivo* and a subset of cells present in the Foxe1KO organoids (Fig. 4C). This analysis also highlights the possible presence of AT2 precursors, reinforcing the immaturity of the system.
Taken together, these observations suggest the presence of AT2-like cells at an early developmental stage, rather than fully differentiated or functional AT2 cells. This interpretation is consistent with the overall developmental immaturity of the lung-like structures observed in our organoid system.

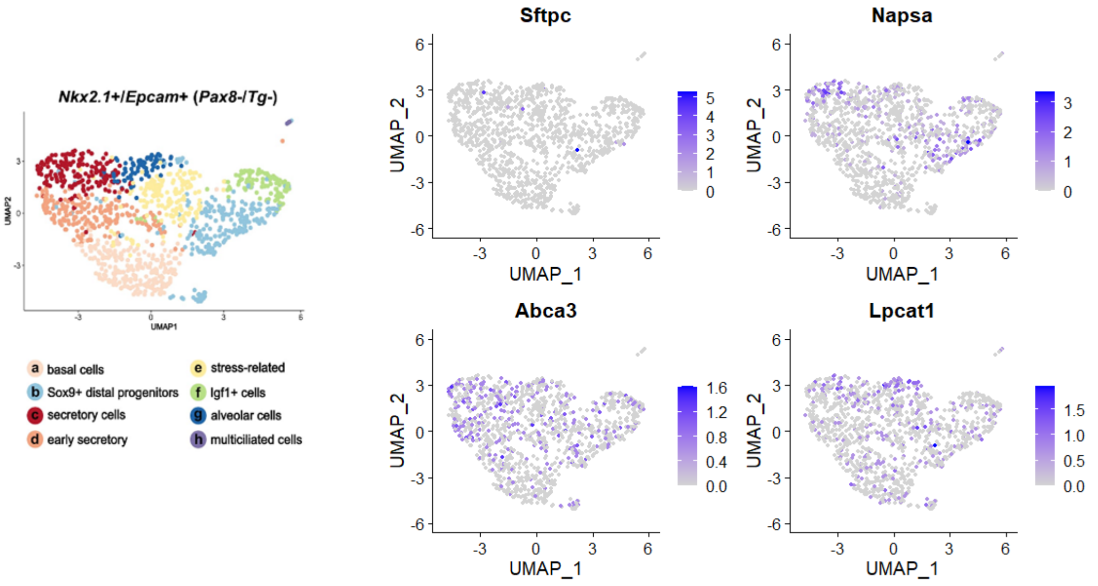


**Figure 7: UMAP expression plots showing Sftpc, Napsa, Abca3 and Lpcat1 lung markers in the Nkx2-1+/Epcam+(Pax8-/Tg-) cell population.**

*(4) For showing proximal lung identities, it would be helpful if the authors can co-stain more than one lineage, such as basal cell together with goblet cell/ciliated cells to reveal potential pseudostratified epithelium.*

We thank the reviewer for this insightful suggestion. Addressing the spatial organization of proximal lung epithelial cell types within the organoids is indeed an interesting aspect. Based on our observations, multiple epithelial cell types do not appear to consistently coexist within the same organoid structure.

Our analyses indicate that many organoids co-express basal cell markers (p63 and Krt5) together with Sox2, but not together with Muc5ac, a marker of goblet cells. This observation may suggest that the *in vitro* system does not fully recapitulate the progressive epithelial maturation and spatial organization seen in vivo, such as the formation of a pseudostratified airway epithelium.

Ideally, this question would be addressed through three-dimensional immunostaining within individual organoid structures to visualize the spatial arrangement of the different epithelial lineages. However, despite several attempts, we were unable to obtain images that would allow reliable interpretation of such co-localization.

Regarding ciliated cells, analysis of the scRNA-seq dataset indicates that they represent a relatively rare population in our cultures, which likely further limits the ability to visualize their spatial organization within organoids.

*Minor points:*

#

*All characterization of in vitro induced thyroid cells should be accompanied by parallel analysis of native thyroid cells (from in vivo mice) that serve as a benchmark for the maturity of the induced cells. Some staining, such as Fig 1F on Tg-I remains quite different from what is reported from in vivo findings.*

We thank the reviewer for this important comment. In our previous work (Antonica et al., *Nature*, 2012), the characterization of thyroid organoids was extensively performed in direct comparison with native mouse thyroid tissue, and all antibodies used in the study were benchmarked using mouse thyroid as a positive control. Regarding the maturity of the thyroid organoids generated *in vitro*, we previously demonstrated both *in vitro* and *in vivo* thyroid hormone (TH) production, confirming the functional capacity of the derived thyroid cells. Although a certain degree of heterogeneity in maturation is observed within WT thyroid organoids, likely reflecting their fetal-like developmental state, these findings support the presence of functionally mature thyrocytes.

To further address the reviewer's concern, we have now included new Tg-I staining images in Fig. 1F, which more clearly illustrate the accumulation of the thyroid hormone precursor within the luminal compartment of follicles derived from WT mESCs.

In addition, we would like to note that the specificity and suitability of the antibodies used to stain native mouse thyroid cells have been validated in several previous studies, including Dathan et al., *Dev Dyn*, 2002; Gérard et al., *Am J Pathol*, 2008; Hartog et al., *Endocrinology*, 1990.

*The labeling of panel E and F in Figure.1 should be switched.*

We thank the reviewer for bringing this to our attention. The labeling of panels E and F in Fig. 1 has been corrected accordingly in the revised manuscript.

***Reviewer #2 (Significance (Required)):***

*This study provided direct in vitro evidence regarding the critical role of Foxe1 for thyroid lineage induction, and suggested its role in balancing thyroid versus lung fate determination. It is thus important to the field of both thyroid and lung developmental and stem cell biology. However, the significance of this study in hindered by the lack of comprehensiveness in the analysis.*

We thank the reviewer for the positive evaluation of our study and for recognizing its relevance to both thyroid and lung developmental biology. To address the concern regarding the comprehensiveness of the analysis, we have carefully revised the manuscript to improve clarity and to better present and discuss the results of our analyses. We believe that these revisions have strengthened the manuscript and improved the overall quality of the study.

***Reviewer #3 (Evidence, reproducibility and clarity (Required)):***

*Costagliola et al. have demonstrated that Foxe1, a transcription factor, plays a key role in the proper differentiation of Nkx2-1 (+) cells into thyroid follicles. They have also revealed that some Foxe1-null/Nkx2-1 (+) cells differentiate into the lung, including airway and alveolar epithelia, in their ES cell-derived organoid system. Although it has already been appreciated that Foxe1*

*contributes to the thyroid development in mice and humans, this excellent study has clarified that its absence, as a result, enhances the differentiation of Nkx2-1 (+) cells into the lung. I have no serious criticisms regarding methodology, results, and interpretation of results. I' d like you to elucidate whether similar findings are obtained even from human ES cell lines in the future.*

We would like to express our sincere gratitude to Reviewer #3 for the positive feedback on our work. We fully agree that it will be important to determine whether similar findings can also be observed using human embryonic stem cell (ESC) systems.

While the mouse model used in this study was first reported in 2012 (Antonica et al., *Nature*, 2012), our group has more recently developed a corresponding system to generate functional thyroid follicular cells from human pluripotent stem cells (Romitti et al., *Nature Communications*, 2022). Using this human platform, we are currently investigating the role of FOXE1, as well as other genes associated with congenital hypothyroidism, in human thyroid development. We anticipate that these studies will provide further insight into the mechanisms controlling thyroid lineage specification and will be the focus of future work.

*Minor comment:*

*1. Fig 3C-E, Fig 6B, D, and F: These figures are so small that the words are almost illegible.*

We thank the reviewer for bringing this to our attention. The figures have been revised to improve readability, and the font sizes have been increased in Fig. 3C–E and Fig. 6B, D, and F in the updated version of the manuscript.

***Reviewer #3 (Significance (Required)):***

*I'm a pathologist who specialize in lung cancer and the stem cells in the distal airway. This paper will probably attract those who are interested in the development of the thyroid or the lung, because the authors have revealed that 1) Foxe1 contributes to the proper thyroid development, and 2) its absence consequently enhances the differentiation of Nkx2-1 (+) cells into the lung.*

We thank the reviewer for this thoughtful comment and for highlighting the potential interest of our study for researchers working in thyroid and lung development. We agree that our findings provide new insight into the role of Foxe1 in thyroid lineage specification and suggest that its absence can shift the differentiation potential of Nkx2.1$^{+}$ progenitors toward a lung epithelial fate. We hope that these results will contribute to a better understanding of the mechanisms regulating cell fate decisions within the anterior foregut endoderm and will be of interest to both the thyroid and lung developmental biology communities.

Dear Sabine,

Thank you for the submission of your revised manuscript to EMBO reports. We have now received the enclosed reports from the referees that were asked to assess it, and I am happy to say that both support the publication of your study now. Referee 2 has one more suggestion that I would like you to address and incorporate before we can proceed with the official acceptance of your manuscript.

A few editorial requests will also need to be addressed:

- Please remove all main and suppl. figures from the ms file. Only the figure legends should be kept at the end of the ms file.

- "Data and code availability" needs to be corrected to "Data Availability Section" (DAS) and needs to be moved to the end of the Methods section. Information on published datasets and the sentence "source data are provided with this ms" need to be removed from this section. Please note that published datasets can be cited and referenced as such (see our guide to authors for more information). Please add the specific URLs for the GSE182337, GSE182480, GSE182676 datasets to the DAS. "Lead contact" and "Materials Availability" need to be removed from the ms file (this information applies to all published papers).

- The conflict of interest subheading needs to be corrected to "Disclosure and Competing Interests Statement"

- The author credits need to be removed from the ms file. All credits need to be entered during online ms submission.

- "data not shown" on page 8 is not allowed per journal policy. Please remove or re-write.

- Please co-submit with your final ms a completed author checklist, which you can download from our author guidelines <https://link.springer.com/journal/44319/submission-guidelines> The completed author checklist will also be part of the transparent peer-review file.

- The FUNDING INFO is not congruent; the following items are listed in the ms, but not in our online system: PDR T.0140.14; PDR T.0230.18, the Fonds d'Encouragement à la Recherche de l'Université Libre de Bruxelles (FER-ULB), Brazilian National Council for Scientific and Technological Development (CNPq; Brazil), MISU-PROL (40005588), please correct; funding info should be part of the Acknowledgments. No separate Funding section heading should be used.

- As figure callout "supplementary" should not be used, please correct. All suppl figures should be called Expanded View (EV) figures instead (Figure EV1, Figure EV2, etc.) and all file names and callouts need to be corrected.

- Table S1 is an Excel file which is missing (but a legend is provided in the ms). If this is a complex Excel file (with lots of rows, columns and sheets) then it should be renamed to Dataset EV1 and uploaded as a Dataset; the legend would then need to be provided in the same Excel file, but in an separate sheet. The other suppl. tables, Table S2-S4 should be part of the Reagents & Tools table that is currently missing.

- The Materials and Methods section should include a separate Reagents and Tools Table file (listing key reagents, experimental models, software and relevant equipment and including their sources and relevant identifiers) and a Methods and Protocols section in which methods should be described using a step-by-step protocol format with bullet points. More information and a downloadable Table template is available in our guide to authors online: https://link.springer.com/journal/44319/submission-guidelines

- Highlights and Lead contact section need to be removed from the ms.

- Summary should be called Abstract.

- METHOD DETAILS should be METHODS.

- The manuscript sections should be in the following order: Title page - Abstract - Introduction - Results - Discussion - Methods - Data Availability - Acknowledgments - Disclosure Statement & Competing Interests - References - Figure Legends - (Main Tables with legends if applicable) - Expanded View Figure Legends.

* Figure Legends - Comments *

- Please note that the exact p values are not provided in the legend of figure 1F. Please provide exact values as reasonable.
- Please note that information related to n is missing in the legends of figures 1F, 3C, E; 4B, D; 5A-D
- Please note that the error bars are not defined in the legend of figure 1F.

I would like to suggest some minor changes to the title and abstract. Please let me know whether you agree with this:

Foxe1 deficiency impairs thyroid fate while supporting lung differentiation

Patterning of mouse (or: mammalian?) endoderm into lung and thyroid lineages depends on the expression of the homeobox domain-containing transcription factor Nkx2-1. However, the gene networks distinguishing the differentiation of those lineages remain unknown. In this work, using mouse (OK?) stem cell lines, scRNA-seq, and transcriptomic and chromatin accessibility profiling, we show that Foxe1 knockout impairs Nkx2-1+ cell differentiation and maturation into thyroid follicular-like cells. Concomitantly, a subset of Foxe1 null/Nkx2-1+ cells follow a lung epithelial differentiation program and form lung-like organoids harboring cells transcriptionally similar to mouse fetal lung types. Chromatin analyses reveal that, while accessibility at the Pax8 locus is reduced, loci associated with lung programs are in an open configuration, indicating that lung fate can be adopted without chromatin remodeling. These findings demonstrate that Foxe1 loss destabilizes thyroid commitment but creates a permissive state in which Nkx2-1+ foregut progenitors can adopt alternative lung fate. Our study illustrates how interplay between transcription factors and chromatin context governs lineage decisions in vitro and provides a platform to investigate mechanisms underlying organ specification and plasticity.

EMBO press papers are accompanied online by A) a short (1-2 sentences) summary of the findings and their significance, B) 2-3 bullet points highlighting key results and C) a synopsis image that is exactly 550 pixels wide and 300 pixels high. The synopsis image should provide a sketch of the major findings, like a graphical abstract. Please note that text needs to be readable at the final size. Please send us this information along with the final manuscript.

I look forward to seeing a final version of your manuscript as soon as possible.

Best regards,
Esther

Esther Schnapp, PhD
Senior Editor
EMBO reports

Referee #1:

I believe the authors have satisfactory answered all issues of concern and revised the manuscript accordingly.

Referee #2:

The reviewer is pleased to see the additional new data and discussion in response to the prior reviewer comments. Given that the authors highlighted the challenges they had with deeper characterization of alveolar type I and II cells and the suggested immaturity of these cells; the authors should add dedicated discussion on this aspect in the manuscript to make sure the readers can properly assess the message conveyed by this study.

Dear Sabine,

Thank you for the submission of your revised manuscript to EMBO reports. We have now received the enclosed reports from the referees that were asked to assess it, and I am happy to say that both support the publication of your study now. Referee 2 has one more suggestion that I would like you to address and incorporate before we can proceed with the official acceptance of your manuscript.

A few editorial requests will also need to be addressed:

- Please remove all main and suppl. figures from the ms file. Only the figure legends should be kept at the end of the ms file. R: The required changes are now included in the new version of the manuscript.

- "Data and code availability" needs to be corrected to "Data Availability Section" (DAS) and needs to be moved to the end of the Methods section. Information on published datasets and the sentence "source data are provided with this ms" need to be removed from this section. Please note that published datasets can be cited and referenced as such (see our guide to authors for more information). Please add the specific URLs for the GSE182337, GSE182480, GSE182676 datasets to the DAS. "Lead contact" and "Materials Availability" need to be removed from the ms file (this information applies to all published papers). R: The required changes are now included in the new version of the manuscript.

- The conflict of interest subheading needs to be corrected to "Disclosure and Competing Interests Statement" R: The new version of the manuscript now contains "Disclosure and Competing Interests Statement".

- The author credits need to be removed from the ms file. All credits need to be entered during online ms submission. R: The information has been removed from the ms.

- "data not shown" on page 8 is not allowed per journal policy. Please remove or re-write. R: “data not shown” has been removed from the ms.

- Please co-submit with your final ms a completed author checklist, which you can download from our author guidelines <https://link.springer.com/journal/44319/submission-guidelines>; The completed author checklist will also be part of the transparent peer-review file. R: The required author checklist file was co-submitted with the ms.

- The FUNDING INFO is not congruent; the following items are listed in the ms, but not in our online system: PDR T.0140.14; PDR T.0230.18, the Fonds d'Encouragement à la Recherche de l'Université Libre de Bruxelles (FER-ULB), Brazilian National Council for Scientific and Technological Development (CNPq; Brazil), MISU-PROL (40005588), please correct; funding info should be part of the Acknowledgments. No separate Funding section heading should be used. R: The funding information is now part of the Acknowledgments.

- As figure callout "supplementary" should not be used, please correct. All suppl figures should be called Expanded View (EV) figures instead (Figure EV1, Figure EV2, etc.) and all file names and callouts need to be corrected. R: The required changes are now included in the new version of the manuscript.

- Table S1 is an Excel file which is missing (but a legend is provided in the ms). If this is a complex Excel file (with lots of rows, columns and sheets) then it should be renamed to Dataset EV1 and uploaded as a Dataset; the legend would then need to be provided in the same Excel file, but in an separate sheet. The other suppl. tables, Table S2-S4 should be part of the Reagents & Tools table that is currently missing. R: The table does not constitute a large and complex Excel file. It is presented as Table EV1.

- The Materials and Methods section should include a separate Reagents and Tools Table file (listing key reagents, experimental models, software and relevant equipment and including their sources and relevant identifiers) and a Methods and Protocols section in which methods should be described using a step-by-step protocol format with bullet points. More information and a downloadable Table template is available in our guide to authors online: https://link.springer.com/journal/44319/submission-guidelines R: The Reagents and Tools table is now found in the Material and Methods section.

- Highlights and Lead contact section need to be removed from the ms. R: The information has been removed from the ms.

- Summary should be called Abstract. R: The change has been incorporated to the ms.

- METHOD DETAILS should be METHODS. R: The change has been incorporated to the ms.

- The manuscript sections should be in the following order: Title page - Abstract - Introduction - Results - Discussion - Methods - Data Availability - Acknowledgments - Disclosure Statement & Competing Interests - References - Figure Legends - (Main Tables with legends if applicable) - Expanded View Figure Legends. R: The sections order have been updated in the ms.

* Figure Legends - Comments *

- Please note that the exact p values are not provided in the legend of figure 1F. Please provide exact values as reasonable. R: The p values were added to the graphs for clarity.

- Please note that information related to n is missing in the legends of figures 1F, 3C, E; 4B, D; 5A-D. R: For figures 1F and 5A-D the n was added to the figures legends. Figures 3C, E; 4B, D; are related to scRNAseq data and the n does not apply in this case.

- Please note that the error bars are not defined in the legend of figure 1F. R: The information is now included in the figure legend.

I would like to suggest some minor changes to the title and abstract. Please let me know whether you agree with this:

Foxe1 deficiency impairs thyroid fate while supporting lung differentiation

Patterning of mouse (or: mammalian?) endoderm into lung and thyroid lineages depends on the expression of the homeobox domain-containing transcription factor Nkx2-1. However, the gene networks distinguishing the differentiation of those lineages remain unknown. In this work, using mouse (OK?) stem cell lines, scRNA-seq, and transcriptomic and chromatin accessibility profiling, we show that Foxe1 knockout impairs Nkx2-1+ cell differentiation and maturation into thyroid follicular-like cells. Concomitantly, a subset of Foxe1 null/Nkx2-1+ cells follow a lung epithelial differentiation program and form lung-like organoids harboring cells transcriptionally similar to mouse fetal lung types. Chromatin analyses reveal that, while accessibility at the Pax8 locus is reduced, loci associated with lung programs are in an open configuration, indicating that lung fate can be adopted without chromatin remodeling. These findings demonstrate that Foxe1 loss destabilizes thyroid commitment but creates a permissive state in which Nkx2-1+ foregut progenitors can adopt alternative lung fate. Our study illustrates how interplay between transcription factors and chromatin context governs lineage decisions in vitro and provides a platform to investigate mechanisms underlying organ specification and plasticity. R: We agree with the modifications and they were incorporated into the ms.

EMBO press papers are accompanied online by A) a short (1-2 sentences) summary of the findings and their significance, B) 2-3 bullet points highlighting key results and C) a synopsis image that is exactly 550 pixels wide and 300 pixels high. The synopsis image should provide a sketch of the major findings, like a graphical abstract. Please note that text needs to be readable at the final size. Please send us this information along with the final manuscript.

**Short summary**

Foxe1 loss disrupts thyroid differentiation and instead promotes lung-like cell specification from Nkx2-1+ foregut progenitors. These findings reinforce the key role of Foxe1 in thyroid lineage commitment and cell fate plasticity.

**Key findings**

- Foxe1 deficiency impairs thyroid follicular cell differentiation and maturation.
- Foxe1-null Nkx2-1+ cells can adopt a lung-like epithelial fate and form lung-like organoids.
- Lung-associated chromatin regions remain accessible even in the presence of Foxe1, and upon Foxe1 loss this permissive chromatin state facilitates activation of the lung differentiation program.

I look forward to seeing a final version of your manuscript as soon as possible.

Best regards,
Esther

Referee #1:

I believe the authors have satisfactory answered all issues of concern and revised the manuscript accordingly.
R: We thank the reviewer 1 for the positive feedback.

Referee #2:

The reviewer is pleased to see the additional new data and discussion in response to the prior reviewer comments. Given that the authors highlighted the challenges they had with deeper characterization of alveolar type I and II cells and the suggested immaturity of these cells; the authors should add dedicated discussion on this aspect in the manuscript to make sure the readers can properly assess the message conveyed by this study.
R: R: We thank the reviewer 1 for the positive feedback and following the reviewer’s comment, we better describe the alveolar cells immature state both in the results section and discussion.

Prof. Sabine Costagliola
Institut de Recherche Interdisciplinaire en Biologie Humaine et Moléculaire (IRIBHM),Université Libre de Bruxelles (ULB)
IRIBHM
808 Lennik street
brussels 1070
Belgium

Dear Sabine,

I am very pleased to accept your manuscript for publication in the next available issue of EMBO reports. Thank you for your contribution to our journal.

Your manuscript will be processed for publication by EMBO Press. It will be copy edited and you will receive page proofs prior to publication. Please note that you will be contacted by Springer Nature Author Services to complete licensing and payment information.

You may qualify for financial assistance for your publication charges - either via a Springer Nature fully open access agreement or an EMBO initiative. Check your eligibility: https://link.springer.com/journal/44319/how-to-publish-with-us

Should you be planning a Press Release on your article, please notify the production team via this form in order to coordinate publication and release dates: https://forms.cloud.microsoft/e/eH4A8JVP6g

If you have any questions, please do not hesitate to contact the Editorial Office. Thank you for your contribution to EMBO Reports.

Best regards,
Esther

Esther Schnapp, PhD
Senior Editor
EMBO reports

------------------------------------------------

>>> Please note that it is EMBO Reports policy for the transcript of the editorial process (containing referee reports and your response letter) to be published as an online supplement to each paper. If you do NOT want this, you will need to inform the Editorial Office via email immediately. More information is available here: https://link.springer.com/partners/embo-press/editorial-policies#Peer%20review

## EMBO Press Author Checklist

| | |
|---|---|
| Corresponding Author Name: Sabine Costagliola | |
| Journal Submitted to: EMBO Reports | |
| Manuscript Number: EMBOR-2026-64085V1-T | |

**USEFUL LINKS FOR COMPLETING THIS FORM**
The EMBO Journal - Author Guidelines
EMBO Reports - Author Guidelines
Molecular Systems Biology - Author Guidelines
EMBO Molecular Medicine - Author Guidelines

**Reporting Checklist for Life Science Articles (updated January**
This checklist is adapted from Materials Design Analysis Reporting (MDAR) Checklist for Authors. MDAR establishes a minimum set of requirements in transparent reporting in the life sciences (see Statement of Task: 10.31222/osf.io/9sm4x). Please follow the journal's guidelines in preparing your
**Please note that a copy of this checklist will be published alongside your article.**

**Abridged guidelines for figures**
**1. Data**
The data shown in figures should satisfy the following conditions:
- the data were obtained and processed according to the field's best practice and are presented to reflect the results of the experiments in an accurate and unbiased manner.
- ideally, figure panels should include only measurements that are directly comparable to each other and obtained with the same assay.
- plots include clearly labeled error bars for independent experiments and sample sizes. Unless justified, error bars should not be shown for technical
- if n<5, the individual data points from each experiment should be plotted. Any statistical test employed should be justified.
- Source Data should be included to report the data underlying figures according to the guidelines set out in the authorship guidelines on Data

**2. Captions**
Each figure caption should contain the following information, for each panel where they are relevant:
- a specification of the experimental system investigated (eg cell line, species name).
- the assay(s) and method(s) used to carry out the reported observations and measurements.
- an explicit mention of the biological and chemical entity(ies) that are being measured.
- an explicit mention of the biological and chemical entity(ies) that are altered/varied/perturbed in a controlled manner.
- the exact sample size (n) for each experimental group/condition, given as a number, not a range;
- a description of the sample collection allowing the reader to understand whether the samples represent technical or biological replicates (including how many animals, litters, cultures, etc.).
- a statement of how many times the experiment shown was independently replicated in the laboratory.
- definitions of statistical methods and measures:
  - common tests, such as t-test (please specify whether paired vs. unpaired), simple χ2 tests, Wilcoxon and Mann-Whitney tests, can be unambiguously identified by name only, but more complex techniques should be described in the methods section;
  - are tests one-sided or two-sided?
  - are there adjustments for multiple comparisons?
  - exact statistical test results, e.g., P values = x but not P values < x;
  - definition of 'center values' as median or average;
  - definition of error bars as s.d. or s.e.m.

**Please complete ALL of the questions below.**
**Select "Not Applicable" only when the requested information is not relevant for your study.**

**Materials**

| Newly Created Materials | Information included in the manuscript? | In which section is the information available? (Reagents and Tools Table, Materials and Methods, Figures, Data Availability Section) |
|---|---|---|
| New materials and reagents need to be available; do any restrictions apply? | Not Applicable | |

| Antibodies | Information included in the manuscript? | In which section is the information available? (Reagents and Tools Table, Materials and Methods, Figures, Data Availability Section) |
|---|---|---|
| For **antibodies** provide the following information:<br>- Commercial antibodies: RRID (if possible) or supplier name, catalogue number and or/clone number<br>- Non-commercial: RRID or citation | Yes | Reagents and Tools Table |

| DNA and RNA sequences | Information included in the manuscript? | In which section is the information available? (Reagents and Tools Table, Materials and Methods, Figures, Data Availability Section) |
|---|---|---|
| **Short novel DNA or RNA including primers, probes**: provide the sequences. | Yes | Reagents and Tools Table |

| Cell materials | Information included in the manuscript? | In which section is the information available? (Reagents and Tools Table, Materials and Methods, Figures, Data Availability Section) |
|---|---|---|
| **Cell lines:** Provide species information, strain. Provide accession number in repository **OR** supplier name, catalog number, clone number, and/**OR** RRID. | Yes | Materials and Methods |
| **Primary cultures:** Provide species, strain, sex of origin, genetic modification status. | Not Applicable | |
| Report if the cell lines were recently **authenticated** (e.g., by STR profiling) and tested for mycoplasma contamination. | Not Applicable | |

| Experimental animals | Information included in the manuscript? | In which section is the information available? (Reagents and Tools Table, Materials and Methods, Figures, Data Availability Section) |
|---|---|---|
| **Laboratory animals or Model organisms:** Provide species, strain, sex, age, genetic modification status. Provide accession number in repository **OR** supplier name, catalog number, clone number, **OR** RRID. | Not Applicable | |
| **Animal observed in or captured from the field:** Provide species, sex, and age where possible. | Not Applicable | |
| Please detail **housing and husbandry conditions**. | Not Applicable | |

| Plants and microbes | Information included in the manuscript? | In which section is the information available? (Reagents and Tools Table, Materials and Methods, Figures, Data Availability Section) |
|---|---|---|
| **Plants:** provide species and strain, ecotype and cultivar where relevant, unique accession number if available, and source (including location for collected wild specimens). | Not Applicable | |
| **Microbes:** provide species and strain, unique accession number if available, and source. | Not Applicable | |

| Human research participants | Information included in the manuscript? | In which section is the information available? (Reagents and Tools Table, Materials and Methods, Figures, Data Availability Section) |
|---|---|---|
| If collected and within the bounds of privacy constraints report on age, sex and gender or ethnicity for all study participants. | Not Applicable | |

| Core facilities | Information included in the manuscript? | In which section is the information available? (Reagents and Tools Table, Materials and Methods, Figures, Data Availability Section) |
|---|---|---|
| If your work benefited from core facilities, was their service mentioned in the acknowledgments section? | Yes | Acknowledgments |

**Design**

| Study protocol | Information included in the manuscript? | In which section is the information available? (Reagents and Tools Table, Materials and Methods, Figures, Data Availability Section) |
|---|---|---|
| If study protocol has been **pre-registered, provide DOI in the manuscript**. For clinical trials, provide the trial registration number **OR** cite DOI. | Not Applicable | |
| Report the **clinical trial registration number** (at ClinicalTrials.gov or equivalent), where applicable. | Not Applicable | |

| Laboratory protocol | Information included in the manuscript? | In which section is the information available? (Reagents and Tools Table, Materials and Methods, Figures, Data Availability Section) |
|---|---|---|
| Provide DOI OR other citation details if **external detailed step-by-step protocols** are available. | Not Applicable | |

| Experimental study design and statistics | Information included in the manuscript? | In which section is the information available? (Reagents and Tools Table, Materials and Methods, Figures, Data Availability Section) |
|---|---|---|
| Include a statement about **sample size** estimate even if no statistical methods were used. | Yes | Figure Legends |
| Were any steps taken to minimize the effects of subjective bias when allocating animals/samples to treatment (e.g. **randomization procedure**)? If yes, have they been described? | Not Applicable | |
| Include a statement about **blinding** even if no blinding was done. | Not Applicable | |
| Describe **inclusion/exclusion criteria** if samples or animals were excluded from the analysis. Were the criteria pre-established?<br><br>If sample or data points were omitted from analysis, report if this was due to attrition or intentional exclusion and provide justification. | Not Applicable | |
| For every figure, are **statistical tests** justified as appropriate? Do the data meet the assumptions of the tests (e.g., normal distribution)? Describe any methods used to assess it. Is there an estimate of variation within each group of data? Is the variance similar between the groups that are being statistically compared? | Yes | Statistical analysis section |

| Sample definition and in-laboratory replication | Information included in the manuscript? | In which section is the information available? (Reagents and Tools Table, Materials and Methods, Figures, Data Availability Section) |
|---|---|---|
| In the figure legends: state number of times the experiment was **replicated** in laboratory. | Yes | Figure Legends |
| In the figure legends: define whether data describe **technical or biological replicates**. | Yes | Figure Legends |

## Ethics

| Ethics | Information included in the manuscript? | In which section is the information available? (Reagents and Tools Table, Materials and Methods, Figures, Data Availability Section) |
|---|---|---|
| Studies involving **human participants**: State details of **authority granting ethics approval** (IRB or equivalent committee(s), provide reference number for approval. | Not Applicable | |
| Studies involving **human participants**: Include a statement confirming that **informed consent** was obtained from all subjects and that the experiments conformed to the principles set out in the WMA Declaration of Helsinki and the Department of Health and Human Services Belmont Report. | Not Applicable | |
| Studies involving **human participants:** For publication of **patient photos**, include a statement confirming that consent to publish was obtained. | Not Applicable | |
| Studies involving experimental **animals**: State details of **authority granting ethics approval** (IRB or equivalent committee(s), provide reference number for approval. Include a statement of compliance with ethical regulations. | Not Applicable | |
| Studies involving **specimen and field samples:** State if relevant **permits** obtained, provide details of authority approving study; if none were required, explain why. | Not Applicable | |

| Dual Use Research of Concern (DURC) | Information included in the manuscript? | In which section is the information available? (Reagents and Tools Table, Materials and Methods, Figures, Data Availability Section) |
|---|---|---|
| Could your study fall under dual use research restrictions? Please check biosecurity documents and list of **select agents and toxins** (CDC): https://www.selectagents.gov/sat/list.htm | Not Applicable | |
| If you used a select agent, is the security level of the lab appropriate and reported in the manuscript? | Not Applicable | |
| If a study is subject to dual use research of concern regulations, is the name of the **authority granting approval and reference number** for the regulatory approval provided in the manuscript? | Not Applicable | |

## Reporting

The MDAR framework recommends adoption of discipline-specific guidelines, established and endorsed through community initiatives. Journals have their own policy about requiring specific guidelines and recommendations to complement MDAR.

| Adherence to community standards | Information included in the manuscript? | In which section is the information available? (Reagents and Tools Table, Materials and Methods, Figures, Data Availability Section) |
|---|---|---|
| State if relevant guidelines or checklists (e.g., **ICMJE, MIBBI, ARRIVE, PRISMA**) have been followed or provided. | Not Applicable | |
| For **tumor marker prognostic studies,** we recommend that you follow the **REMARK** reporting guidelines (see link list at top right). See author guidelines, under 'Reporting Guidelines'. Please confirm you have followed these guidelines. | Not Applicable | |
| For **phase II and III randomized controlled trials**, please refer to the **CONSORT** flow diagram (see link list at top right) and submit the CONSORT checklist (see link list at top right) with your submission. See author guidelines, under 'Reporting Guidelines'. Please confirm you have submitted this list. | Not Applicable | |

## Data Availability

| Data availability | Information included in the manuscript? | In which section is the information available? (Reagents and Tools Table, Materials and Methods, Figures, Data Availability Section) |
|---|---|---|
| Have **primary datasets** been deposited according to the journal's guidelines (see 'Data Deposition' section) and the respective accession numbers provided in the Data Availability Section? | Yes | Data Availability |
| Were **human clinical and genomic datasets** deposited in a public access-controlled repository in accordance to ethical obligations to the patients and to the applicable consent agreement? | Not Applicable | |
| Are **computational models** that are central and integral to a study available without restrictions in a machine-readable form? Were the relevant accession numbers or links provided? | Not Applicable | |
| If publicly available data were reused, provide the respective **data citations in the reference list**. | Yes | Reference list |
