# Supplementary material for: Foxe1 deficiency impairs thyroid fate while supporting lung differentiation
Source: EMBO Rep. 2026 Jun 21;27(15):4462–92. doi: 10.1038/s44319-026-00841-1 (PMC13458771; doi:10.1038/s44319-026-00841-1)
Supplement: Supplementary file 2 [file 44319_2026_841_MOESM6_ESM.pdf]

Expanded View Figures

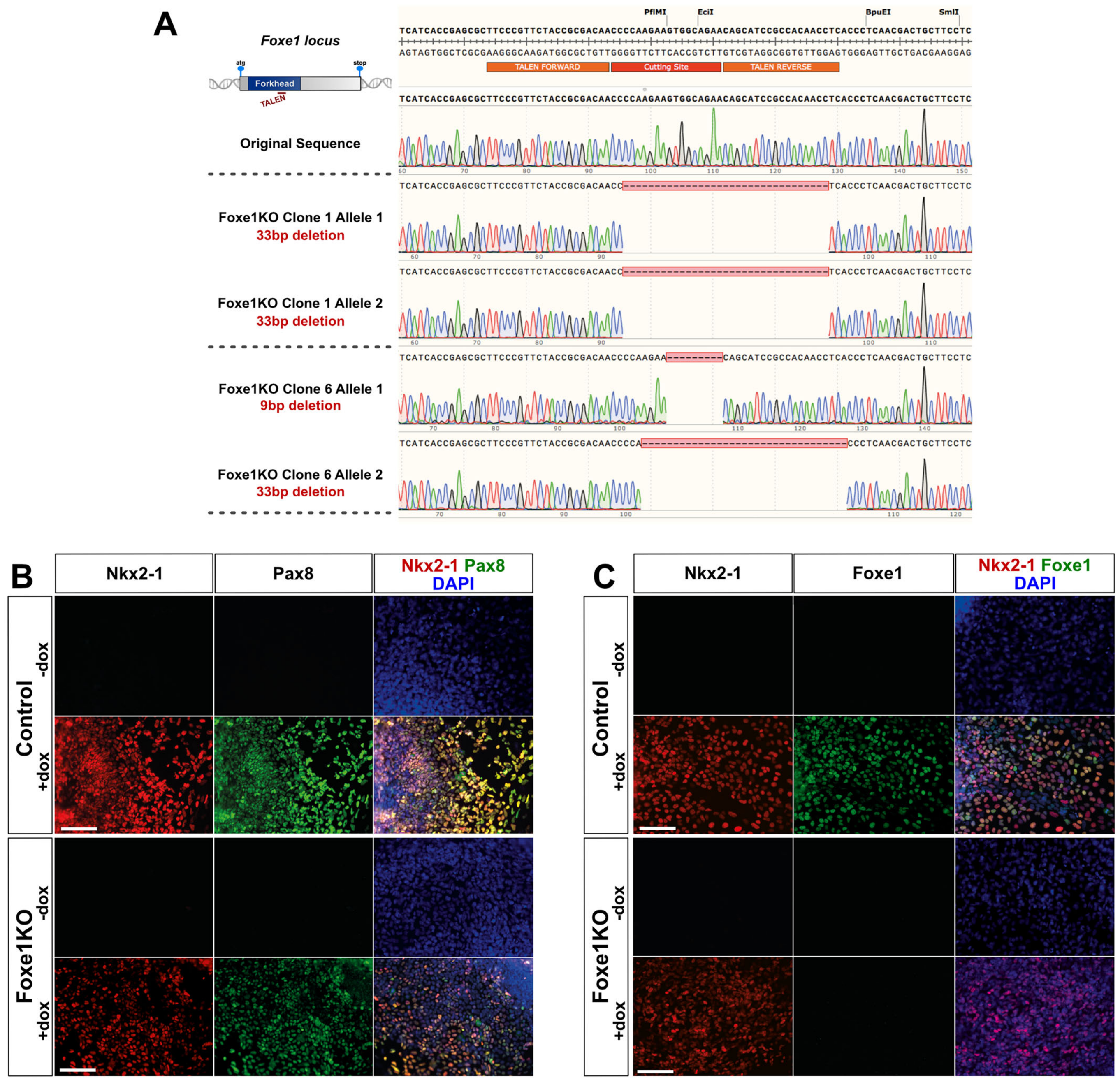

Figure EV1. Generation and validation of Foxe1 KO mESC lines.

(**A**) Genomic profiling of two Foxe1 KO mESC clones. One clone presents a homologous deletion of 33 bp, and the second one possesses a heterozygous deletion of 9 and 33 bp at the Foxe1 locus, respectively. (**B, C**) Immunostaining of control and Foxe1KO mESCs at day 7, after Dox-mediated induction of Nkx2.1-Pax8 during 3 days (day 4–day 7). (**B**) The Dox-inducible co-expression of NKX2.1 and PAX8 is not altered by genome-editing manipulations in mutated clones. (**C**) Foxe1KO mESCs express NKX2.1, while Foxe1 protein expression is abolished (day 7). Scale bars: 150 µm.

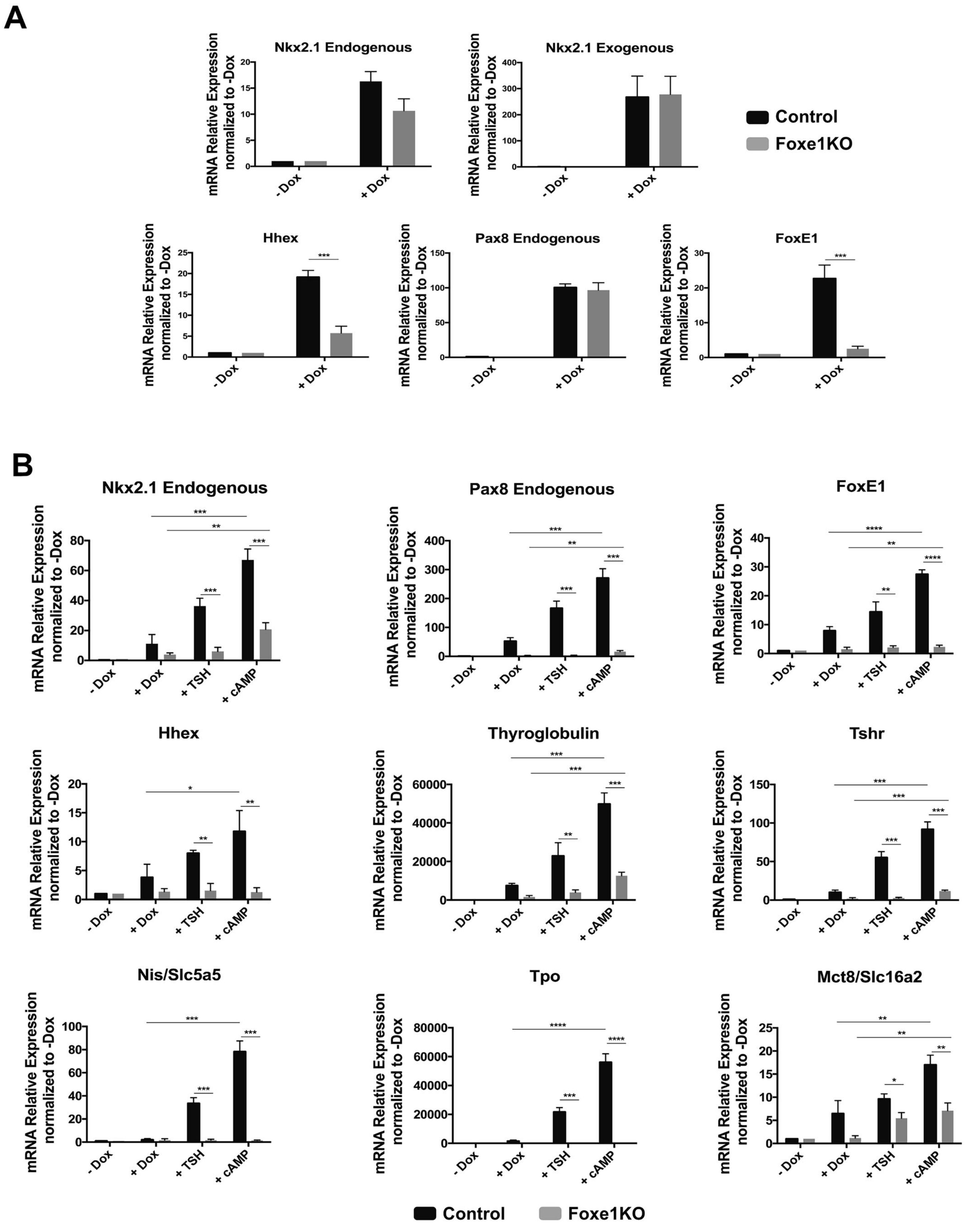
A
Nkx2.1 Endogenous
Nkx2.1 Exogenous
Hhex
Pax8 Endogenous
FoxE1
mRNA Relative Expression normalized to -Dox
-Dox
+ Dox
Control
Foxe1KO
B
Nkx2.1 Endogenous
Pax8 Endogenous
FoxE1
Hhex
Thyroglobulin
Tshr
Nis/Slc5a5
Tpo
Mct8/Slc16a2
- Dox
+ Dox
+ TSH
+ cAMP
Control
Foxe1KO

© The Author(s)

Figure EV2. Foxe1 depletion in mESCs abolishes thyroid follicle differentiation.

RT-qPCR analyses of thyroid-expressed genes in control and Foxe1KO cells at day 7 (**A**) and day 22 (**B**) after Dox-mediated induction (day 4–day 7) followed by TSH (+TSH) or 8-br-cAMP (+cAMP) treatment until day 22. (**A**) At day 7 of differentiation, downregulation of some thyroid genes such as Hhex and Foxe1 is already observed in Foxe1KO cells, whereas Nkx2-1 and Pax8 endogenous expression is not affected. Observe that Dox-mediated induction of Nkx2-1/Pax8 was successful since exogenous expression of Nkx2-1 is not affected in both lineages. (**B**) Expression of endogenous thyroid genes at day 22 is drastically reduced in Foxe1KO cells (+TSH and +cAMP conditions) in comparison with the control line. Relative expression of each transcript is presented as fold change compared to untreated cells (−Dox) (mean +/−SEM; $n = 3$ biological replicates). Unpaired t test was used for statistical analysis. *$P < 0.05$, **$P < 0.01$, ***$P < 0.001$. Dox doxycycline.

© The Author(s)

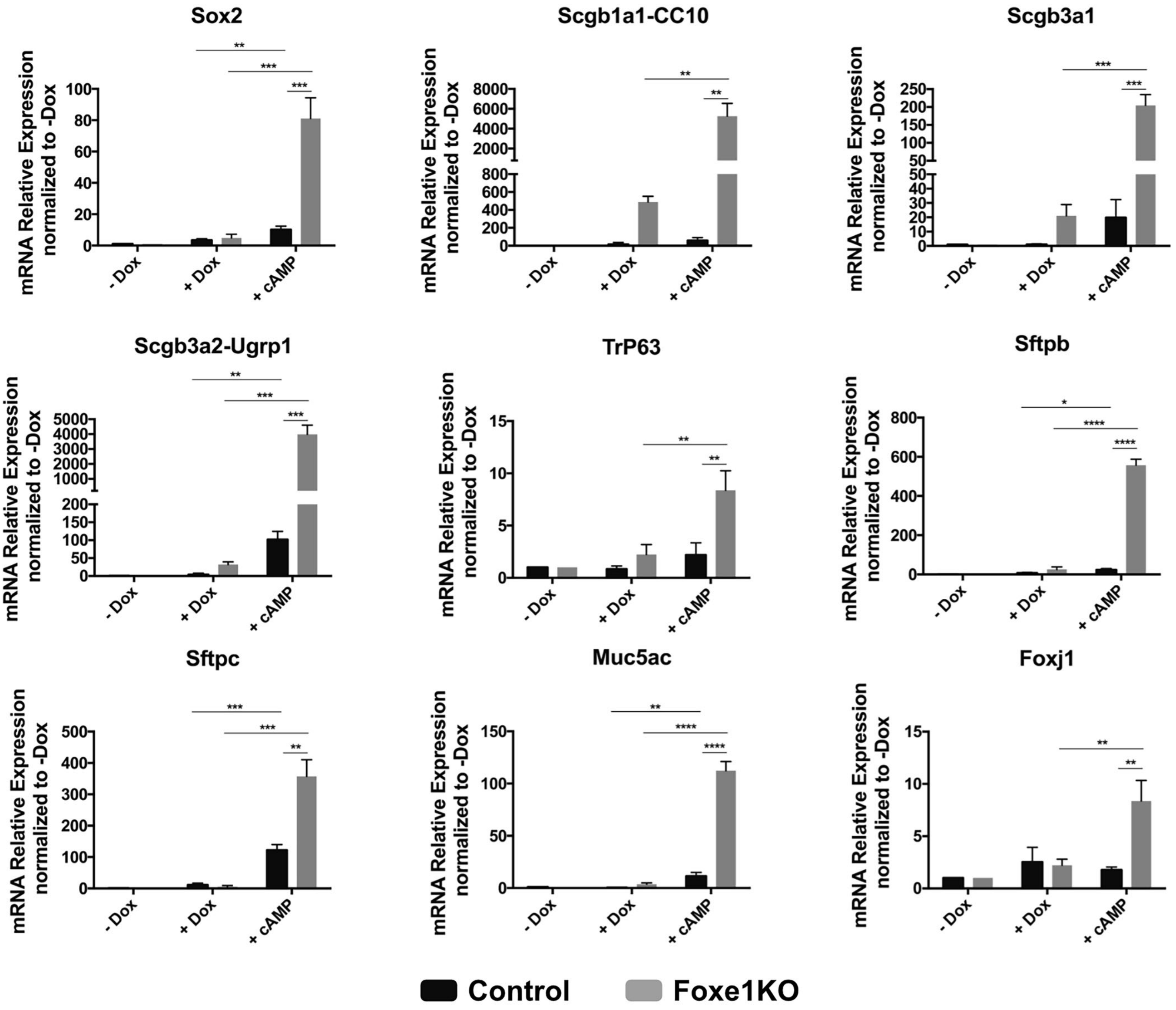

Figure EV3. In vitro generation of pulmonary structures.

RT-qPCR of in vitro-generated Foxe1KO structures after Dox-mediated induction of Nkx2.1-Pax8 followed by 8-br-cAMP treatment until day 22. mRNA expression of endogenous relevant pulmonary genes at day 22. Observe upregulation of lung-related markers in the +cAMP condition compared with untreated cells (-Dox) and dox-induced only condition (+Dox). Relative expression of each transcript is presented as fold change compared to untreated cells (−Dox) (mean +/−SEM; $n = 3$ biological replicates). Unpaired *t* test was used for statistical analysis. $^{*}P < 0.05$, $^{**}P < 0.01$, $^{***}P < 0.001$.

 © The Author(s)

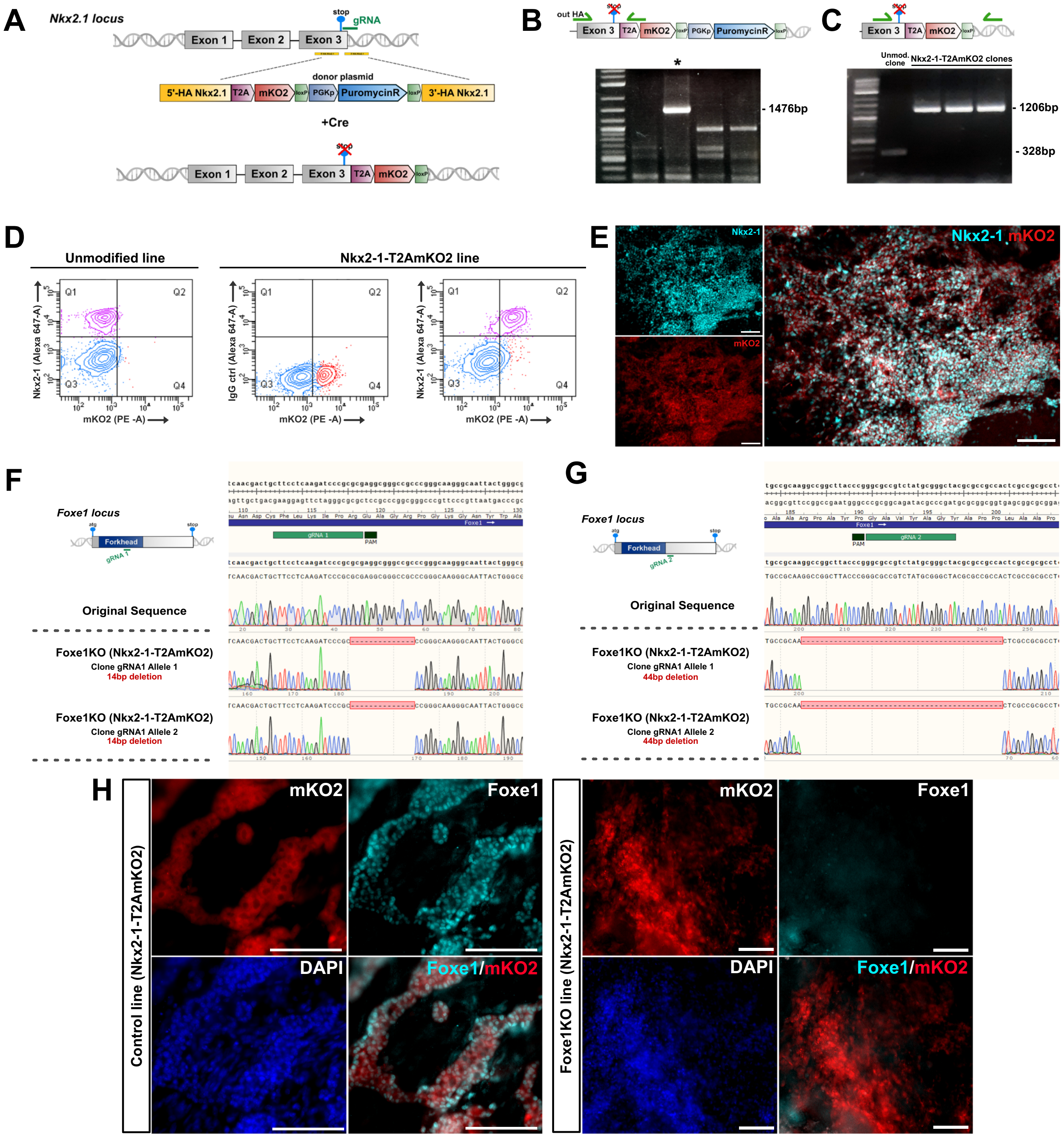

Figure EV4. Generation of endogenous Nkx2-1 reporter lines.

(**A**) Schematic representation of the CRISPR-mediated knock-in strategy to insert T2AmKO2 cassette in 3′-UTR region of Nkx2-1 loci. (**B**, **C**) PCR screening of knock-in generated clones before (**B**) and after (**C**) PuroR gene excision. Asterisk (*) on the image in (**B**) indicates a correctly integrated clone. (**D**, **E**) Validation of the Nkx2-1-T2AmKO2 line. (**D**) Cells from day 14 of the thyroid differentiation protocol were subjected to Nkx2-1 immunostaining followed by flow cytometry. Observe a double-positive stained population in Q2, only in the cAMP-treated cells derived from the Nkx2-1-T2AmKO2 line. (**E**) Images showing Nkx2-1 and mKO2 co-staining at day 10 2D culture. (**F**–**H**) Generation of Foxe1KO/Nkx2-1 reporter line. (**F**, **G**) Genomic profiling of a Foxe1KO/Nkx2-1 reporter clones obtained with guide RNAs targeting inside (**F**) or outside (**G**) the Forkhead domain. (**H**) Foxe1 immunostaining of control and Foxe1KO/Nkx2-1 reporter cells subjected to thyroid differentiation (day 22). Absence of Foxe1 protein expression is observed in Foxe1KO cells. Scale bars: 100 μm.

© The Author(s)

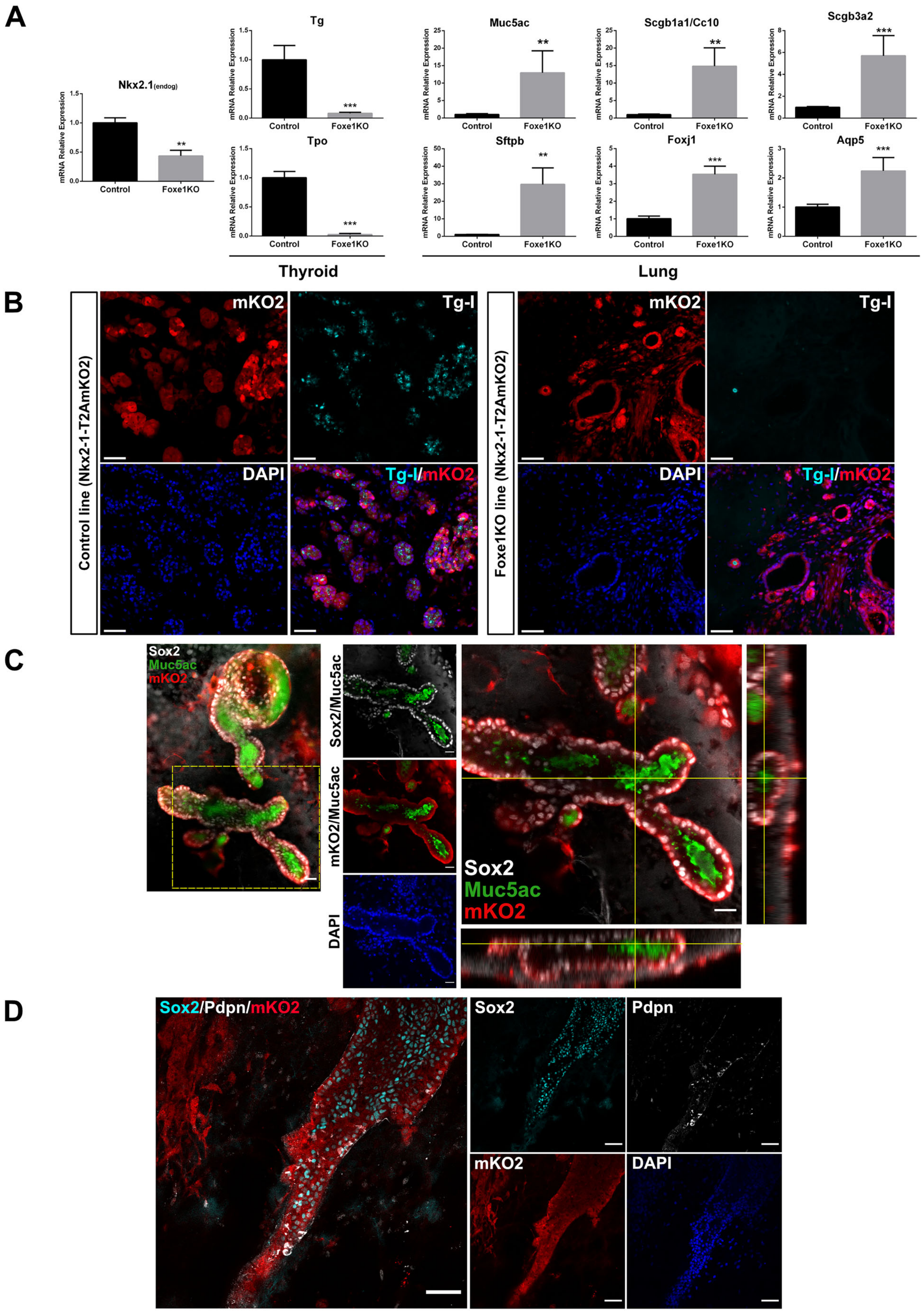
A
Nkx2.1(endog)
Tg
Tpo
Muc5ac
Sftpb
Scgb1a1/Cc10
Foxj1
Scgb3a2
Aqp5
mRNA Relative Expression
Control
Foxe1KO
Thyroid
Lung
B
Control line (Nkx2-1-T2AmKO2)
Foxe1KO line (Nkx2-1-T2AmKO2)
mKO2
Tg-I
DAPI
Tg-I/mKO2
C
Sox2
Muc5ac
mKO2
Sox2/Muc5ac
mKO2/Muc5ac
DAPI
D
Sox2/Pdpn/mKO2
Sox2
Pdpn
mKO2
DAPI

 © The Author(s)

Figure EV5. Thyroid and lung differentiation in control and Foxe1KO Nkx2-1 reporter lines.

(**A**) RT-qPCR analyses of thyroid and lung expressed genes in control and Foxe1KO Nkx2-1 reporter lines. Observe the reduction of Tg and Tpo thyroid genes, whereas lung markers are upregulated in Foxe1KO cells. (**B**) Control Nkx2-1 reporter cells differentiated into thyroid follicles, containing iodinated-Tg (Tg-I) in the lumen. mKO2 immunostaining corresponds to the Nkx2-1 endogenous expression. (**C**, **D**) Foxe1KO Nkx2-1 reporter cells differentiate into airway organoids. (**C**) Immunostaining for Sox2, mKO2 and Muc5ac. Higher magnification and orthogonal views by confocal microscopy of the lung organoid depicted in the image on the right. (**D**) Immunostaining for Sox2, Pdpn, and mKO2. Scale bars: 50 µm (**B**, **D**), 25 µm (**C**).

© The Author(s)

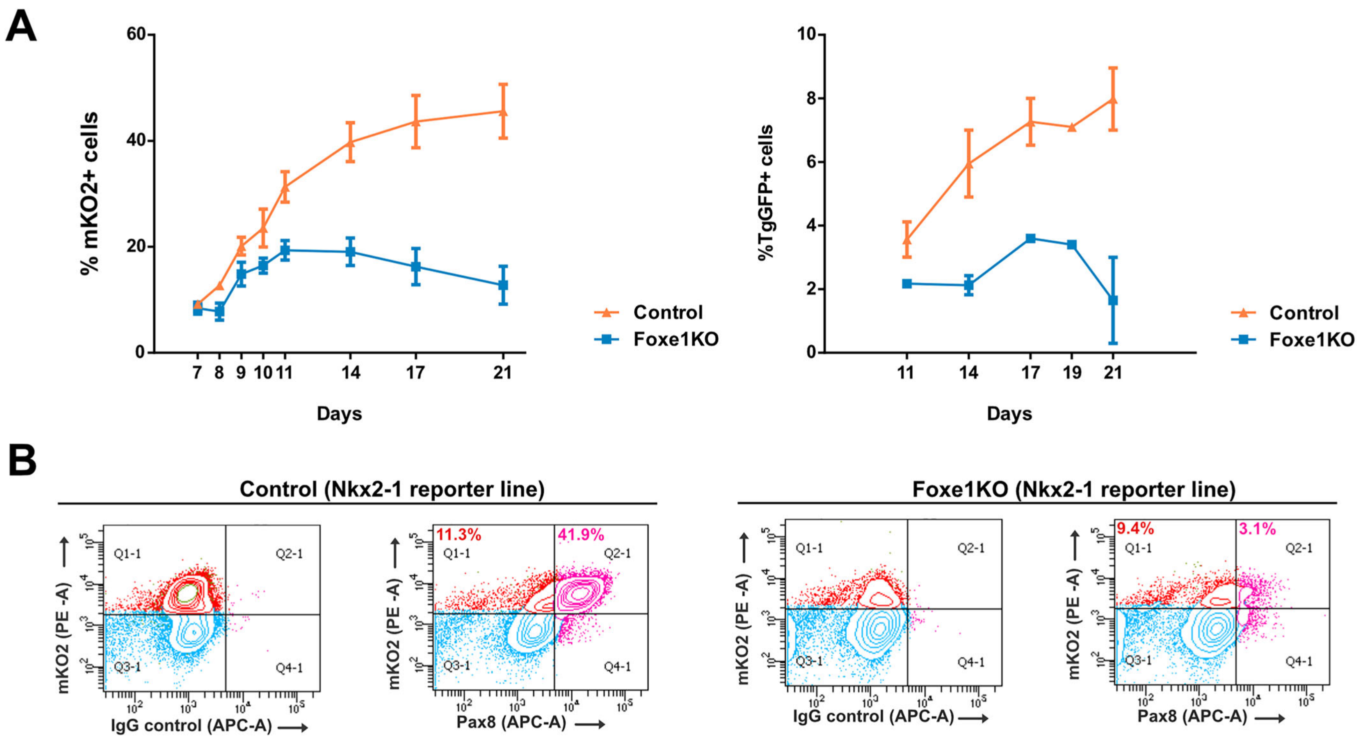

Figure EV6. Quantification of Nkx2-1-expressing cells in control and Foxe1KO/Nkx2-1 reporter lines.

(**A**) Flow cytometry quantification of cells expressing Nkx2-1 reporter (mKO2 +, left) and bovineTg reporter (TgGFP +, right) in control and Foxe1KO lines. (**B**) Pax8 immunostaining followed by flow cytometry analyses at day 22. Numbers shown in Q2-1 quadrant correspond to the percentage of double-positive Pax8 (APC) and mKO2 (PE) in control and Foxe1KO cells.

 © The Author(s)

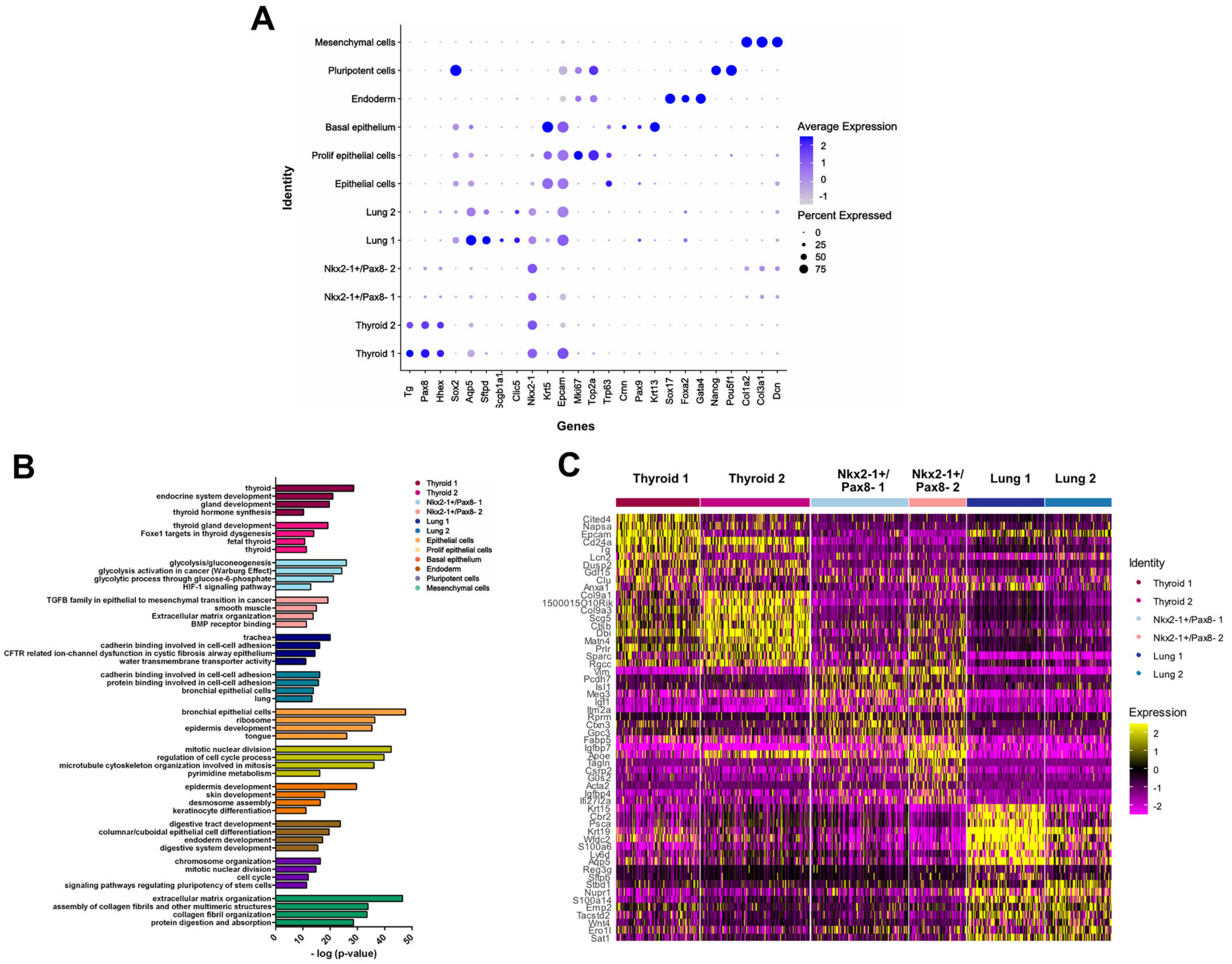

Figure EV7. scRNAseq profiling of Foxe1KO cells.

(**A**) Average expression per cluster of selected markers used to define cluster identity. (**B**) Gene Ontology (GO) and pathway analysis performed with the top 50 differentially expressed genes in each cluster. (**C**) Heatmap of the top 50 differentially expressed genes in each cluster expressing Nkx2-1.

© The Author(s)

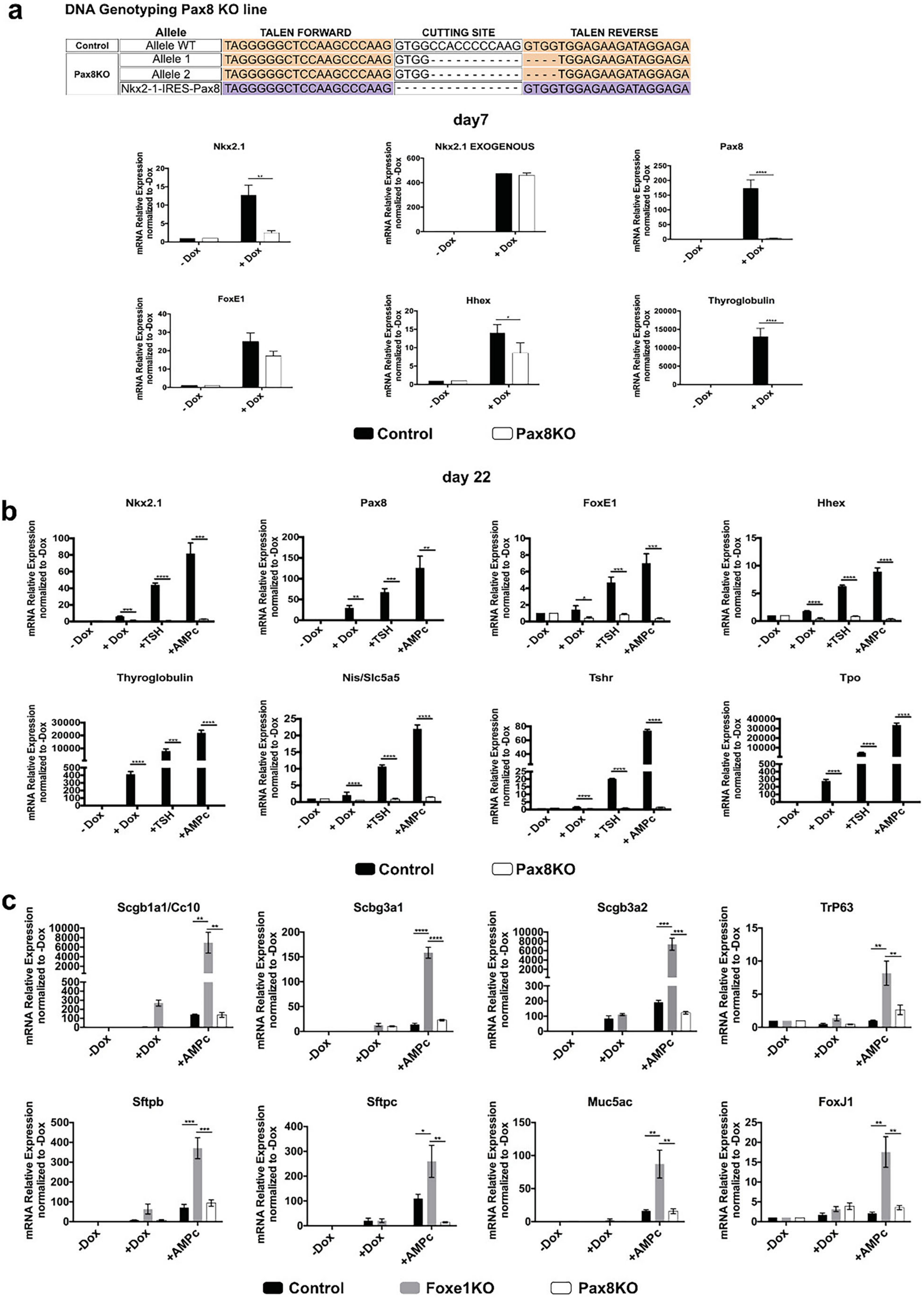
a
DNA Genotyping Pax8 KO line
Allele
TALEN FORWARD
CUTTING SITE
TALEN REVERSE
Control
Allele WT
TAGGGGGCTCCAAGCCCAAG
GTGGCCACCCCCAAG
GTGGTGGAGAAGATAGGAGA
Pax8KO
Allele 1
TAGGGGGCTCCAAGCCCAAG
GTGG- - - - - - - - - - -
- - - - TGGAGAAGATAGGAGA
Allele 2
TAGGGGGCTCCAAGCCCAAG
GTGG- - - - - - - - - - -
- - - - TGGAGAAGATAGGAGA
Nkx2-1-IRES-Pax8
TAGGGGGCTCCAAGCCCAAG
GTGGTGGAGAAGATAGGAGA
day7
Nkx2.1
Nkx2.1 EXOGENOUS
Pax8
FoxE1
Hhex
Thyroglobulin
mRNA Relative Expression normalized to -Dox
- Dox
+ Dox
Control
Pax8KO
day 22
b
Nkx2.1
Pax8
FoxE1
Hhex
Thyroglobulin
Nis/Slc5a5
Tshr
Tpo
- Dox
+ Dox
+TSH
+AMPc
Control
Pax8KO
c
Scgb1a1/Cc10
Scbg3a1
Scgb3a2
TrP63
Sftpb
Sftpc
Muc5ac
FoxJ1
-Dox
+Dox
+AMPc
Control
Foxe1KO
Pax8KO

 © The Author(s)

Figure EV8. Invalidation of Pax8 impairs thyroid formation but is not sufficient to drive lung differentiation.

(**A**) Genomic profiling of Pax8KO mESCs obtained by TALEN technology. (**B**) Expression of endogenous thyroid markers for control and Pax8KO differentiated cells after Dox-mediated induction of Nkx2.1-Pax8 during 3 days (day 4–day 7) followed by 8-br-cAMP treatment until day 22. A robust downregulation of all thyroid genes is observed in Pax8KO differentiated cells compared to controls. (**C**) Expression of endogenous lung markers in control, Foxe1KO, and Pax8KO differentiated cells following the same differentiation protocol. Expression of lung-related genes are not induced in cells of Pax8KO line. Relative expression of each transcript is presented as fold change compared to untreated cells (-Dox) at day 7 (mean +/−SEM; $n = 3$ biological replicates). Unpaired *t* test was used for statistical analysis. *$P < 0.05$, **$P < 0.01$, ***$P < 0.001$.
